# Heterodinuclear Zn(II), Mg(II) or Co(III) with Na(I) Catalysts for Carbon Dioxide and Cyclohexene Oxide Ring Opening Copolymerizations

**DOI:** 10.1002/chem.202101140

**Published:** 2021-07-09

**Authors:** Wouter Lindeboom, Duncan A. X. Fraser, Christopher B. Durr, Charlotte K. Williams

**Affiliations:** ^1^ Department of Chemistry University of Oxford Chemistry Research Laboratory Oxford UK

**Keywords:** carbon dioxide, heterodinuclear complexes, ROCOP, sodium, catalysis

## Abstract

A series heterodinuclear catalysts, operating without co‐catalyst, show good performances for the ring opening copolymerization (ROCOP) of cyclohexene oxide and carbon dioxide. The complexes feature a macrocyclic ligand designed to coordinate metals such as Zn(II), Mg(II) or Co(III), in a Schiff base ‘pocket’, and Na(I) in a modified crown‐ether binding ‘pocket’. The 11 new catalysts are used to explore the influences of the metal combinations and ligand backbones over catalytic activity and selectivity. The highest performance catalyst features the Co(III)Na(I) combination, [*N*,*N*′‐bis(3,3’‐triethylene glycol salicylidene)‐1,2‐ethylenediamino cobalt(III) di(acetate)]sodium (**7**), and it shows both excellent activity and selectivity at 1 bar carbon dioxide pressure (TOF=1590 h^−1^, >99 % polymer selectivity, 1 : 10: 4000, 100 °C), as well as high activity at higher carbon dioxide pressure (TOF=4343 h^−1^, 20 bar, 1 : 10 : 25000). Its rate law shows a first order dependence on both catalyst and cyclohexene oxide concentrations and a zeroth order for carbon dioxide pressure, over the range 10–40 bar. These new catalysts eliminate any need for ionic or Lewis base co‐catalyst and instead exploit the coordination of earth‐abundant and inexpensive Na(I) adjacent to a second metal to deliver efficient catalysis. They highlight the potential for well‐designed ancillary ligands and inexpensive Group 1 metals to deliver high performance heterodinuclear catalysts for carbon dioxide copolymerizations and, in future, these catalysts may also show promise in other alternating copolymerization and carbon dioxide utilizations.

## Graphical abstract

**A series of heterodinuclear M/Na catalysts** for cyclohexene oxide and carbon dioxide copolymerization are reported. The best catalyst comprises Co(III)/Na(I), coordinated by the macrocyclic ligand, and showing activity and selectivity values at the upper end of reported values for 1 bar carbon dioxide pressure copolymerizations. These catalysts operated without co‐catalyst and maintain high activity at low loadings.

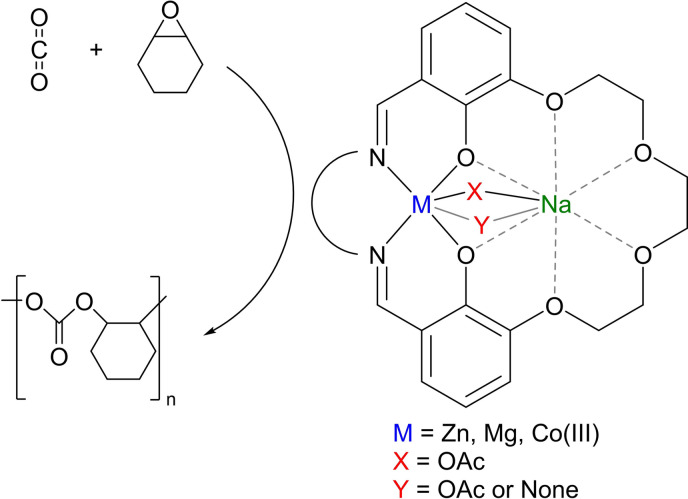

## Introduction

The efficient conversion of carbon dioxide to useful products is a lynchpin of sustainable chemistry.^[1]^ It is driven by the large quantities of carbon dioxide emitted by a range of industries and the need to both valorize and (re)cycle it into new products.[^1^, ^2^] One promising carbon dioxide utilization is its copolymerization with epoxides to form polycarbonates or polyether carbonates.^[3]^ These materials are useful either as low molecular weight polyols for polyurethane synthesis, replacing petrochemical polyols in the manufacture of household goods, home insulation and footwear, or as high molar mass polymers and networks which are elastomers or ductile plastics.^[4]^ Life cycle assessments indicate such utilizations save carbon dioxide emissions both directly and by avoiding the use of the petrochemical raw materials.^[4b]^ The materials evolution and expansion to new applications requires highly selective, active and controllable polymerization catalysts.[^3a^, ^3b^, ^5^] Heterogeneous catalysts can show high activities but with the trade‐off of much lower carbon dioxide uptake, the requirement for high carbon dioxide pressures and limited polymerization control.[^3a^, ^6^] Homogeneous catalysts combine high carbon dioxide uptake, outstanding activity and impressive polymerization control facilitating production of sophisticated and precise polymers and copolymers.^[3a]^ Leading homogeneous catalysts include β‐diiminate di‐Zn(II) complexes, [Co(III)/Cr(III)(salen)X/PPNX, where X=halide/arylalkoxide/carboxylate] catalyst systems which are either bicomponent or feature ‘tethered’ ionic groups and organoboron/PPNX, where X is defined as above, catalyst systems again either bicomponent or tethered.[^3a^, ^3b^, ^7^] We have investigated dinuclear complexes of Zn(II), Mg(II), Co(II/III) and Fe(III), all coordinated by a diphenolate tetra(amine) macrocyclic ligand, the best of which combine high activity at low carbon dioxide pressure (including at 1 bar CO_2_ pressure) and high polymerization control.^[8]^ Recently, we have discovered that some heterodinuclear catalysts, notably those of Zn(II)Mg(II) or Co(II)Mg(II), show intermetallic synergy resulting in higher activity than the homodinuclear catalyst analogues.[^8^, ^9^] Nonetheless, this catalytic synergy remains restricted to specific metal combinations, since catalysts featuring Zn(II) combined with Li(I), Na(I), K(I), Ca(II), Al(III), Ga(III) or In(III) show lower activity than the analogous di‐Zn(II) catalyst.^[10]^ The most active heterodinuclear catalyst, Co(II)Mg(II), showed polymerization kinetic data that indicates synergy arises from differentiated ‘roles’ for each metal in the catalysis; Mg(II) minimizes the transition state entropy and Co(II) lowers the transition state enthalpy.^[11]^ This catalyst showed unprecedented low pressure activity of 1205 h^−1^ (120 °C, 1 bar, 0.05 mol%) rising to a field‐leading value of 12,500 h^−1^ at higher pressure (140 °C, 20 bar, 0.05 mol%).

Okuda and Mashima also investigated hetero‐multimetallic catalysts, in particular a trizinc‐cerium catalyst showed high activity for CHO/CO_2_ ROCOP.^[12]^ In 2020, Mashima and Nozaki reported a Co(II)_3_Nd(III) catalyst that showed an activity of 1625 h^−1^ (130 °C, 20 bar, 0.005 mol%).^[13]^ Although these heteronuclear polymerization catalysts show impressive performances, better understanding of how to design heterometallic catalysts is needed and, in particular, the influences of ancillary ligands and metal combinations must be investigated.

This work targets new ancillary ligands which are dinucleating macrocycles featuring two different coordination ‘pockets’ each targeting different metals. The ligands combine a Schiff base site, featuring diphenolate‐di(imine/amine) donors, for the coordination of first row transition metals, M(II) or M(III), or Mg(II) and a second binding pocket featuring a tetra‐ether moiety to coordinate Group 1 metals. These ligands, and derivatives, have been widely explored for coordination of UO_2_, lanthanides(III) or M(II) ions (M=Ni, Cu, Zn, Co) with Group 1 (Li, Na, K) or 2 (Ba) metals; the transition metal redox potentials depended upon the second metal selection.^[14]^ Various transition metal (II) or Group 1 or 2 metals with lanthanides (III) were used to investigate intermetallic magnetic/electronic interactions.[^14e^, ^15^] Recently, Yang and co‐workers investigated heterodinuclear complexes of Co(II)/M(IV)/Ni(II)/Fe(II) with alkali/ne earth metals [Na(I), K(I), Ca(II), Sr(II) and Ba(II)] with particular focus on moderation of redox potential values.^[16]^ In 2020, our team reported new Co(III)/M(I) complexes (M=Na, K, Rb, Cs), coordinated by one of these macrocyclic ligands, as high activity PO/CO_2_ ROCOP catalysts (800 h^−1^, 70 °C, 30 bar, 0.025 mol%).^[17]^ The best catalyst shows excellent tolerance to chain transfer agents (diols) allowing for the preparation of either high molecular weight PPC or low molecular weight polyols.

Here, heterodinuclear catalysts featuring either M(II) or M(III) centres with Na(I) are investigated capitalizing on the low cost, light‐weight and earth‐abundance of sodium. So far, in carbon dioxide/epoxide ring‐opening polymerization catalysis there is scant research into the use of Group 1 metals either on their own or in heterodinuclear combinations.^[17]^ Yet, the commonly accepted dinuclear polymerization mechanisms propose one metal should be sufficiently Lewis acidity to coordinate and active the epoxide and, in this regard, sodium(I) complexes should be explored since the metal has a good precedent for epoxide coordination within crown‐ether moieties.^[18]^ This work therefore targets these new complexes as a means to prepare high activity and selectivity cyclohexene oxide/carbon dioxide ROCOP catalysts.

## Results and Discussion

**Scheme 1:**
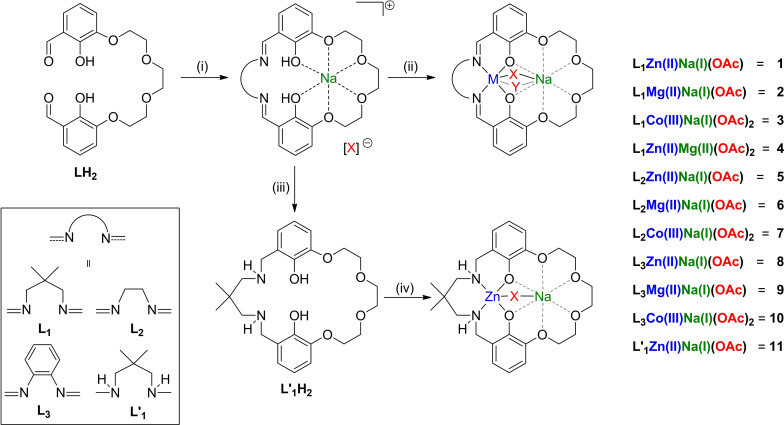
Synthesis of heterodinuclear complexes **1**–**11** (i) Na(OAc), diamine, 60 °C, MeOH, 3 h. (ii) M(OAc)_n_, MeOH, 25 °C, 1 h, 15–79 %. (iii) 20 equiv. NaBH_4_, MeOH, 25 °C, 2 h. (iv) Na(OAc)_,_ Zn(OAc)_2_, 25 °C, 3 h, 65 %.

The macrocyclic pro‐ligand, **LH_2_**, was synthesized, according to literature procedures, and isolated in 43 % yield.[^14a^, ^14g^] The complexes were synthesized using a new approach where LH_2_ was first reacted with sodium acetate, the relevant diamine and, subsequently, the second metal acetate to form the new heterodinuclear complexes in good/excellent yields (see Scheme 1 and Supporting Information for further information on the syntheses). Two systematic series of complexes were targeted, either featuring the same backbone diamine linker and coordinated to Zn(II), Mg(II) or Co(III) (**1**–**3**; **5**–**7**; **8**–**10**) or featuring a particular metal combination with different backbone linkers (e. g. Zn(II)Na(I) with **L_1_** =**1**, **L_2_** =**5**, **L_3_** =**8** or **L’_1_** =**11**). When preparing the Co(III)/Na(I) complexes (**3**, **7**, **10**), the ligand was reacted with Co(II)(OAc)_2_ and after oxidation, in air, the desired Co(III)/Na(I) complexes were isolated (occasionally residual Co(II) complexes were removed during purification). Complex **11** features a macrocyclic ligand with a diamine linker and was prepared by reduction of the free Schiff base macrocycle (NaBH_4_) with the new ligand being reacted with sodium and zinc acetate, at room temperature in methanol, to yield **11** in 65 % yield (see Supporting Information for experimental details).

**Figure 1:**
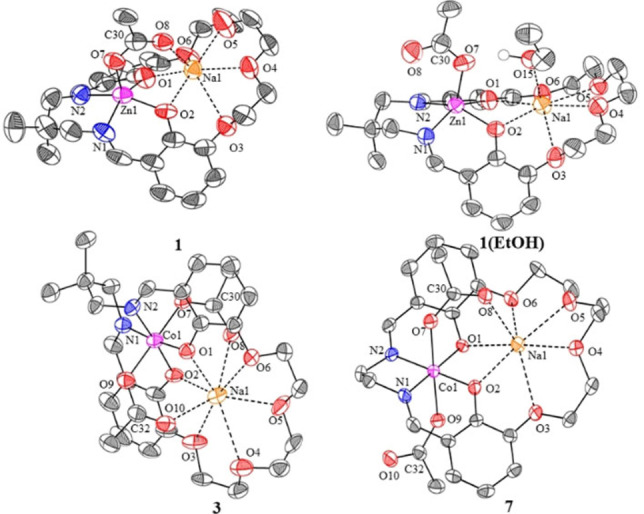
Molecular structures, determined by X‐ray diffraction experiments, presented as thermal displacement ellipsoid plots (50 % probability) for **1**, **1** (EtOH), **3** and **7**. H‐atoms are removed for clarity and color coding used is M (pink), Na (orange), O (red), N (blue), C (grey).

These macrocyclic ligands are particularly useful for isolating pure heterodinuclear complexes since the different donors (Schiff base/ether) allow for selective and specific metal coordination in the two ‘pockets’. It is also worth emphasis that all the heterodinuclear complexes were isolated pure, i. e. without any detectable contamination by homodinuclear counterparts. Complex formation was confirmed using ^1^H NMR spectroscopy, which were characterized by the disappearance of the pro‐ligand's aldehyde resonance and the formation of the desired imine resonance (8.67–8.29 ppm) and by the formation of new acetate and linker resonances (Figures S1‐S10). Complexes were fully characterized by ^1^H, ^13^C, COSY, HSQC and HMBC NMR spectroscopy (Figures S1–50). All complexes show ^1^H NMR spectra with only a single imine resonance and most show three crown‐ether resonances indicating they have symmetrical, time‐averaged structures. Complexes **4**, **6**, **9** and **11** all display additional ether methylene resonances, presenting as a large multiplet in the corresponding region, consistent with lower symmetry complexes likely due to particular conformations of the ether moieties being favored (Figures S4, S6, S9 and S11). Complexes **2** and **4** show fluxional NMR spectra at room temperature (CDCl_3_ or C_2_D_2_Cl_4_), due to the flexible propene linker groups (Figures S2 and S4). These complexes were characterized at higher temperatures (328–398 K) where averaged resonances were observed. In contrast, **3**, produces well‐defined NMR spectra, at room temperature, due to the two acetate ligands resulting in a coordinatively saturated ‘locked’ structure, consistent with that observed in the solid state (Figures 1 and S3). ^13^C{^1^H} NMR spectra for all complexes were fully assigned with the use of HSQC and HMBC. Each shows a distinct acetate resonance, at around 180 ppm, and imine peaks, from 160–170 ppm (Figures S12–S21). The complexes featuring **L_3_** (**8**–**10**) displayed very low solubility, between 298 K and 398 K, and so were characterization by CP‐MAS ^13^C NMR spectroscopy. For **10**, this solid state ^13^C NMR spectrum shows two different acetate peaks in excellent agreement with the two distinct acetate environments observed in the solid‐state structure characterized by X‐ray diffraction of **7** (Figure 1 and S51). The complexes show molecular ions in the ESI mass spectra consistent with ionization resulting in loss of an acetate ligand (Figure S52 and S53). The cobalt catalysts also undergo reduction under the mass spectrometry conditions to form cobalt(II) complexes; this phenomenon was observed previously for the Co(III)/K(I) catalyst.^[17]^ All complexes also have IR spectra showing symmetric and asymmetric acetate stretches (Figure S54–S56).

Single crystals, suitable for X‐ray diffraction experiments, were obtained for **1**, **3**, and **11** by the slow evaporation of chloroform solutions; **1** (EtOH) by the slow evaporation of an ethanol solution and **7** by the slow diffusion of pentane into a methylene chloride solution of catalyst. All structures confirm the formation of metallate complexes at Zn(II) or Co(III), respectively (Figure 1). Both phenolate groups are anionically coordinated to the transition metal (resulting in shorter M−O1 and M−O2 bond lengths than the Na1−O1 and Na1−O2 bond lengths). Also, the acetate ligands are also anionically coordinated by the transition metal, as evidenced by the unsymmetrical acetate ligand C−O bond distances (C30−O7 vs. C30−O8 or C32‐O9 vs. C32‐O10) and shorter M−O7 (M−O9) bond distances compared with Na−O8 (or Na−O10) bond lengths (Figure S57–S60, Table S2–S8). This effect is also observed in the structure of **1** (EtOH), where the acetate is anionically coordinated at the zinc centre and the ethanol molecule is coordinated at the sodium. This complex shows a greater distance between the Zn(II) and Na(I) atoms compared to **1** which features a bridging acetate ligand. The formation of metallate complexes is significant for the catalysis because the sodium ion is expected to show higher Lewis acidity and may, therefore, be pre‐disposed towards epoxide coordination. Support for this notion comes from the coordination of the ethanol molecule at the sodium, rather than zinc site in complex **1** (EtOH). It is also relevant to note that cobaltate catalysts were long‐proposed in carbon dioxide, as well as anhydride, epoxide ring opening copolymerizations using catalysts/co‐catalyst combinations but so‐far these species eluded structural characterization.[^7a^, ^7h^, ^7q^, ^19^] Hence, the structures isolated in this work are likely to be relevant to the active site species present using M(III)(salen)X/PPNX co‐catalyst systems.

Almost all the complexes show dinuclear, but monomeric, structures in the solid state, except **11** which forms acetate bridged polymeric structure (Figures S60). All the zinc complexes (**1**, **1** (EtOH), and **11**) show square pyramidal geometries at the Zn(II) site and heptacoordinate Na(I). The Co(III), which has two acetate ligands, show different binding modes depending on the imine linker. Catalyst **3** features two bridging κ_2_ acetates and a short Co(III)‐Na(I) separation of 3.194 Å. In contrast, **7** shows both a κ_2_ and κ_1_ acetate ligand and has a greater Co(III)−Na(I) separation of 3.388 Å. Both complexes feature octahedral cobaltate centres, but show distinctly different coordination geometries at sodium due to the different acetate binding modes. **11** forms a coordination polymer with bridging acetate ligands, either causing, or resulting from, significant distortion of the solid‐state structure, with highly unsymmetrical binding of sodium to the crown‐ether moiety (Figure S60).

The new complexes were tested as catalysts for the copolymerization of cyclohexene oxide (CHO) and CO_2_, with experiments conducted at 1 bar pressure and 100 °C using 10 equivalents of *trans*‐1,2‐cyclohexanediol as chain transfer agent and neat epoxide (Table 1). For most complexes, the catalyst loading was 0.1 mol% (i. e. 1 : 1000, catalyst:CHO), but for the more active Co(III)Na(I) catalysts lower loadings were applied to avoid entering viscosity limited kinetic regimes over fixed time period experiments. For the series of complexes, a range of activity values were observed with turnover frequencies (TOFs) varying from 0–1590 h^−1^ and selectivity for poly(cyclohexene carbonate) (PCHC) formation from 43–>99 %. In general, the onset of *trans*‐cyclic carbonate formation only becomes significant above 100 °C, although the barrier was somewhat lower for selected catalysts (Table S8). The series of heterodinuclear zinc catalysts were all moderately active, with TOF=23–42 h^−1^, and all showed high selectivity. For these zinc complexes, the flexible C_3_ backbone linker, for example in **1**, results in a higher polymerization rate (Table 1, entries 1–3), while use of the diamine variant **11** led to a slight decrease in activity alongside a concomitant decrease in selectivity (Table 1, entries 1 and 4). All the magnesium catalysts show very low activity, with TOF=6–7 h^−1^, and low selectivity for PCHC formation, 43–73 %, the latter driven by competitive *trans*‐cyclic carbonate formation (Table 1, entries 5–7).

**Table 1:** Polymerization data for CHO/CO_2_ ROCOP.^[a]^

Entry	Cat.	t [h]	PCHC Selec. [%]^[b]^	TON^[c]^	TOF [h^−1^]^[d]^	*M*_n_ [g.mol^−1^] [*Ð*]^[e]^
1	**1**	8	96	335 (±17)	42 (±2)	2500 [1.16]
2	**5**	8	94	235 (±12)	29 (±1)	1700 [1.13]
3	**8**	8	97	265 (±13)	33 (±2)	2000 [1.17]
4	**11**	14	93	318 (±16)	23 (±1)	2500 [1.13]
5	**2**	24	58	168 (±8)	7 (±1)	900 [1.14]
6	**6**	24	48	177 (±9)	7 (±1)	400 [1.43]
7	**9**	24	43	140 (±7)	6 (±1)	400 [1.33]
8	**4**	24	–	0	0	n.d.
9	**3**	14	95	581 (±29)	42 (±2)	3000 [1.17]
10^[f]^	**7**	0.5	>99	795 (±40)	1590 (±80)	5300 [1.07] 2200 [1.05]
11^[g]^	**7**	1	>99	4343 (±434)	4343 (±434)	15700 [1.03] 6700 [1.17]
12^[h]^	**10**	2	94	176 (±9)	88 (±4)	900 [1.19]
13^[i]^	L_a_MgZn	6	>99	438	98	12700 [1.04] 5100 [1.16]
14^[j]^	L_a_MgCo^II^	0.67	>99	465	699	1600 [1.15]
15^[k]^	L_b_Zn_3_Ce	3	>99	900	300	15000 [1.20]
16^[l]^	L_c_Co^III^(X)/^*n*^Bu_4_NX	6	>99	522	87	19100 [1.17]

[a] Catalysis conditions: catalyst : CHD : CHO 1 : 10 : 1000, 100 °C, 1 bar pressure CO_2_ and in neat epoxide; [b] Selectivity for PCHC against *trans‐*cyclohexene carbonate (no ether observed). Measured by integration of ^1^H NMR resonances for cyclic carbonate (*δ* 4.00 ppm) and ether linkages (*δ* 3.45 ppm) against PCHC (*δ* 4.65 ppm); [c] Turnover number (TON)=moles of CHO consumed/moles catalyst, moles of CHO consumed determined by, determined by the addition of integrals of ^1^H NMR resonances of cyclic carbonate (*δ* 4.00 ppm) and PCHC (*δ* 4.65 ppm) over addition of CHO (*δ* 3.05 ppm), cyclic carbonate (*δ* 4.00 ppm) and PCHC (*δ* 4.65 ppm), multiplied by initial moles of CHO; [d] Turnover frequency (TOF)=TON/time; [e] Determined by SEC, in THF, calibrated against narrow *M*_n_ polystyrene standards; dispersity given in square brackets; [f] 1 : 10 : 4000; [g] 1 : 10 : 25000, 120 °C, 20 bar CO_2_; [h] 0.033 mol% catalyst loading; [i] This literature catalyst was tested at 0.1 mol% catalyst, 80 °C and 1 bar CO_2_;^[10a]^ [j] 0.05 mol% catalyst loading, 20 equiv. CHD, 100 °C and 1 bar CO_2_;^[11]^ [k] This literature catalyst was tested at 0.05 mol% catalyst, 100 °C and 3 bar CO_2_;^[12]^ [l] This literature catalyst was tested at 0.02 mol% catalyst, 50 °C and 1 bar CO_2_.^[7e]^ For the chemical structures of all the literature catalysts, see Figure S61. GPC traces can be found Figures S62–S64.

When the magnesium is localized in the tetra‐ether coordination ‘pocket’ in complex **4**, there was no activity at all (Table 1, entry 8) which is unexpected given the success of other macrocyclic ZnMg complexes.[^8d^, ^10a^, ^11^] The lack of reactivity could be due to coordinative saturation of the magnesium(II) center, which has a strong preference for octahedral coordination, preventing epoxide coordination.^[20]^ Furthermore, sodium(I) has a much weaker binding association than Mg(II), which has among the strongest binding associations to oxygens, which may be relevant to the metal's abilities to accelerate epoxide coordination and insertion steps in the catalytic cycle.[^20^, ^21^] Thus, the ZnMg complex is inert using this ligand but the same ligand yields an active catalyst for the ZnNa combination.

The variation in activities for the cobalt series of complexes shows a greater than 30‐fold rate acceleration for the catalysts with ethene linkers, and to a lesser extent higher rates for phenylene linkers compared to propene. The most active catalyst, **7**, shows a TOF of 1523 h^−1^ and >99 % selectivity for carbon dioxide uptake (Table 1, entry 10). To optimize its performance, polymerizations were conducted in a stainless‐steel reactor, with improved stirring efficiency and 20 bar carbon dioxide, and this allowed the catalyst to reach a TOF of 4343 h^−1^ at 120 °C (Table 1, entry 11). Indeed, it's amongst the most highly active catalysts yet reported in this field using 1 bar pressure of carbon dioxide.[^11^, ^12^, ^13^] Comparisons with literature catalysts are more complex since studies are not run under identical conditions, but selected data taken using conditions closest to those used in this study reveals the excellent performance of **7** (Table 1). For example, compared with the previously reported heterodinuclear L_a_MgZn catalyst, **7** shows >10 times greater activity and it shows approximately twice the activity of the recently reported heterodinuclear L_a_MgCo^II^ catalyst.^[11]^ Although at higher temperatures (120 °C) L_a_MgCo^II^ shows a higher activity of 1205 h^−1^ and outperforms catalyst **7** (Table S8). Compared with literature Co(III)(salen)(X)/co‐catalyst system, such as L_c_Co^III^(X)/^*n*^Bu_4_NX, **7** remains stable at higher temperatures resulting in higher activities and these are achieved without any co‐catalyst.^[7b]^ Catalyst **7** also shows five times greater activity than the tetranuclear Zn(II)Ce(III) catalyst, L_b_Zn_3_Ce.^[12]^

In comparison to other catalysts operating at CO_2_ pressures of >10 bar, **7** shows more average performances. For example, L_a_MgCo^II^ achieved a TOF of 12,462 h^−1^, at 140 °C and 20 bar CO_2_ pressure, and it maintained >99 % carbonate selectivity and 0.05 mol% catalyst loading.^[11]^ This performance equates with a three‐fold higher rate than **7** at >10 times lower loading. Under equivalent pressure, catalyst loading and at 120 °C, a Co(III)Co(II) catalyst showed a similar activity to **7** of 4200 h^−1^.[^9b^, ^11^] Rieger and co‐workers reported a di‐zinc catalyst showing a record‐breaking activity of 155,000 h^−1^, at 30 bar CO_2_ pressure and 100 °C, although this value was achieved in the initial phases of polymerization (low conversions) and for conditions where carbonate selectivity was slightly lower (88 %).^[22]^ Notably, subsequent reports from the same group using similar di‐zinc catalysts showed more consistent TOFs of around 6000 h^−1^.^[23]^

**Figure 2:**
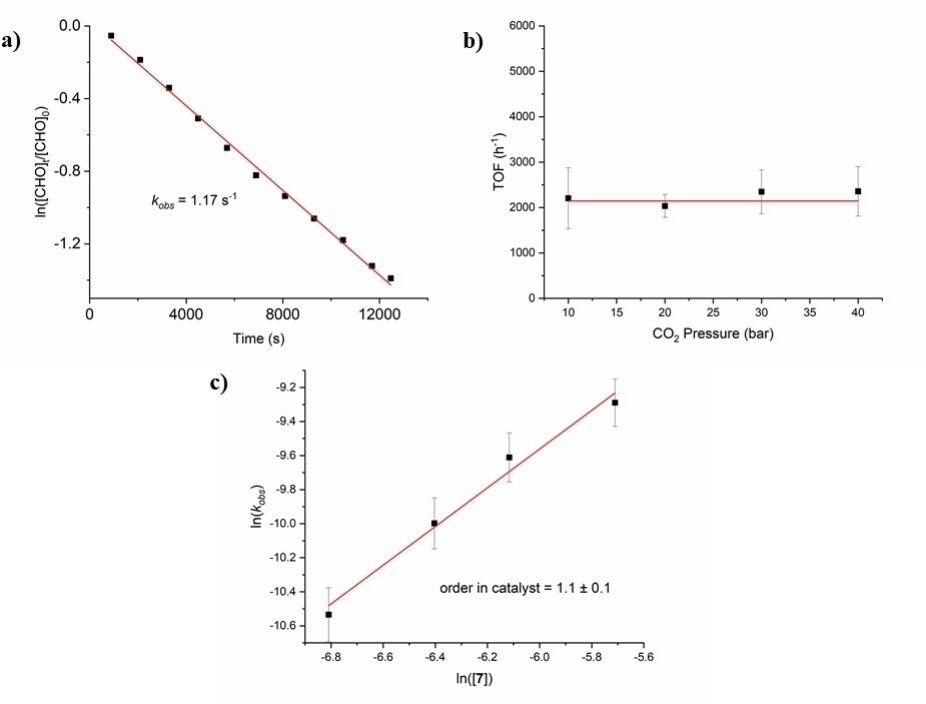
Plots used to analyse the polymerization kinetics and determine the reaction orders in various monomers. (A) Semilogarithmic plot of cyclohexene oxide concentration vs. time with a linear fit to data indicative of a first order dependence on cyclohexene oxide concentration. (B) Plot of activity (TOF) vs. pressure of carbon dioxide, over the range 10–40 bar with a constant value consistent with zero order in CO_2_ pressure. (C) Logarithmic plot of pseudo first order rate coefficient, *k_obs_* vs. concentration of 7 and the linear fit to the data, used to determine a first order dependence on catalyst concentration.

To better understand the enhanced activity of **7**, its polymerization kinetics were studied using in situ ATR‐IR spectroscopy allowing the collection of polymerization versus time data (Figure S65). The order in epoxide was determined, using a 3.33 M CHO solution, 1.66 mM of catalyst, in diethyl carbonate (total volume 10 mL) at 100 °C, by monitoring the increase in concentration of an absorption due to polycarbonate formed (1330, 1160 and 988 cm^−1^). The concentration data was plotted using a semi‐logarithmic integrated rate law treatment (5–70 % conversion) and the linear fit indicates a first order dependence of the rate on the concentration of cyclohexene oxide. The dependence of the rate on carbon dioxide pressure was determined, using neat epoxide and 0.395 mM catalyst concentration at 100 °C, over the pressure range 10–40 bar CO_2_. This range was chosen as it is the standard used for many other catalysts for CO_2_/epoxide ROCOP.[^11^, ^23^, ^24^] All reactions were performed in steel reactors which utilize mechanical stirring, unfortunately it is not feasible to apply this set‐up with a constant pressure below 10 bar. Over the pressure range studied, there was no statistically significant correlation between CO_2_ pressure and rate, indicating a zero‐order dependence; it should be noted that because the reactor set‐up is static after charging with gas there is some error in data points particularly at lower pressures where gas consumption occurs rapidly (Figure 2B). A range of different catalyst concentrations, from 1.10 to 3.31 mM, using 3.33 M CHO in diethyl carbonate (DEC), at 1 bar pressure and 100 °C, were tested. In each case, the pseudo first order rate coefficient, *k_obs_*, was determined from semi‐logarithmic plots of conversion vs. time. The double logarithmic plot of rate coefficient vs. concentration shows a linear fit, consistent with a first order dependence on catalyst concentration. Overall, the data is consistent with a second order rate law (Eq. (1)) and is similar to that observed for the Co(III)K(I) catalyst for PO/CO_2_ ROCOP.^[17]^

(1) $$Rate=k{\left[Cat\right]}^{1}{\left[CHO\right]}^{1}{\left[{CO}_{2}\right]}^{0}$$

The polymerization kinetics and rate law suggests the rate determining step likely involves epoxide ring‐opening by a metal carbonate intermediate.[^3c^, ^8c^] Such a rate law and mechanistic interpretation was previously observed for other heterodinuclear catalysts and is consistent with differentiated roles for the metals in the catalysis.[^11^, ^17^] On the basis of the solid state structure and the formation of cobaltate (or zincate) complexes in all cases, it is proposed that the Lewis acidic sodium serves as the site for epoxide coordination (Figure 1) and the carbonate nucleophile is provided by the second metal with reactivity/activity following the trend Mg(II)<Zn(II)<Co(III).

Comparing the most active Co(III)Na(I) catalyst with well‐known Co(III)(salen)(X)/PPNX catalyst systems, which were very successfully optimized in the literature, reveals a new catalyst design: replacing the ionic co‐catalyst with a Group 1 metal centre coordinated close to the cobaltate by exploiting a dinucleating ancillary ligand.[^7a^, ^7m^, ^25^] Catalyst **7** also benefits from the use of inexpensive, light‐weight and abundant Na(I). Though the ‘18‐crown‐6’‐like tetra‐ether cavity size is more commonly applied to potassium, it is known that strong coordination to sodium is also possible.^[26]^ By correctly employing an ancillary ligand to coordinate Na(I) adjacent to the Co(III) active site, addition or tethering of ionic co‐catalysts is obviated. The ability to replace and remove co‐catalysts is important since the best catalysts feature rather expensive and esoteric salts, most notably PPNX species. By incorporating the'18‐crown‐6’‐like moiety into the macrocycle the catalyst shows increased rate and metal cooperativity as compared to use of Co(III)salen complexes applied with crown ether coordinated Group 1 metals, as shown by Li and co‐workers, where the major product was cyclic carbonate.^[27]^ In future, other di‐ or polynucleating ligands should be explored making use of the Group 1 metal concept to replace co‐catalysts and enhance activity and selectivity values. A further benefit of catalyst **7** is that it maintains high activity and Co(III) speciation even at elevated temperatures. In contrast, bicomponent Co(III)(salen)(X)/PPNX catalyst systems were shown to undergo irreversible thermally activated Co(III) reduction at temperatures above 60 °C.[^7e^, ^28^]

This investigation of new heterodinuclear catalysts resulted in the isolation and structural characterization of new complexes and in all cases the transition metal is speciated as a metallate species. This finding is significant since metallates were previously proposed as the active sites for M(III)(salen)X/PPNX catalyst systems.[^7b^, ^29^] For example, a [Cr(III)(salen)(azide)]^−^/PPN^+^ complex was isolated, and structurally characterized, by reaction of the Cr(III) complex with the PPN(azide) salt.^[30]^ Lee and co‐workers reported a Co(III)(salen)(X) catalyst system featuring four specially tethered quaternary ammonium salts and proposed that the complex operated via a cobaltate intermediate, although such a species was not isolated or structurally characterized.[^7a^, ^7q^] Both in the previous literature and in the case of these heterodinuclear catalysts, it is proposed that the formation of a cobaltate(III) complex enhances the nucleophilicity of the Co(III)‐carbonate intermediate and accelerates epoxide ring‐opening. It is proposed that the high activity resulting from catalyst **7** arises from the different roles for the two metals and is enhanced by a short Co(III)‐Na(I) (3.388 Å). In future, this new class of catalyst should be explored using other combinations of metals, for example replacing Co(III) with Fe(III), Al(III), Ti(III) or Cr(III) centres, which all have some precedent in this carbon dioxide copolymerization catalysis, as well as investigating the optimum Group 1 metal combination.[^8a^, ^30^, ^31^] These new heterodinuclear catalysts are also expected to show high activity for related heterocycle/heterocumulene ring‐opening copolymerizations, including epoxide/anhydride, epoxide/COS and/or heavier congener combinations to access other oxygenated and heteroatom containing polymers. It has also been observed that many of the successful ROCOP catalysts may also be used to copolymerize mixtures of epoxide, anhydride, carbon dioxide and lactone, using switch catalysis, to access multi‐block polymers.^[32]^ Thus, these new catalysts should be explored using a range of other monomers to diversify the product scope and range for carbon dioxide containing polymers.

## Conclusions

A series of high activity heterodinuclear catalysts, combining Na(I) with Zn(II), Mg(II) or Co(III), for the ring‐opening copolymerization of carbon dioxide and cyclohexene oxide were investigated. The use of a macrocyclic ligand featuring two binding pockets allowed for selective synthesis of only the heterodinuclear complexes: the Na(I) is coordinated by a tetra‐ether moiety, while the second metal is coordinated by the schiff base portion of the macrocyle. The most active catalyst, Co(III)/Na(I), showed field‐leading rates and selectivity values particularly when applied under low carbon dioxide pressures. The rate law and solid‐state structural data support a rate determining step in which sodium coordinates the cyclohexene oxide and is attacked by the Co(III)‐carbonate intermediate. The new catalyst types are beneficial compared to other Co(III) catalysts since they replace ionic salts, such as PPNCl, and obviate complex salt ‘tethering’ processes which complicate catalyst preparations. In contrast, these catalysts feature the Co(III) and Na(I) centres ligated by a common macrocyclic ligand and positioned close to one another so as to modify and moderate reactivity appropriately. Future investigations should target a broad range of other metallate catalysts, combining transition metals/main group elements with alkali or alkaline earth metals to understand the potential for heterodinuclear catalysts to deliver high carbon dioxide uptakes, high rates and polymerization control. These new carbon dioxide/epoxide copolymerization catalysts are also likely to show good activity in other copolymerizations, using heterocumulenes, heterocycles and cyclic anhydrides, as well as to lactone or lactide ring‐opening polymerizations allowing access to new materials and structures derived from carbon dioxide.

## Experimental Section

Experimental and characterisation details can be found in the Supporting Information.

Deposition Numbers 2073147 (for **1**), 2073148 (for **1**(EtOH)), 2073149 (for **3**), and 2073150 (for **11**) contain the supplementary crystallographic data for this paper. These data are provided free of charge by the joint Cambridge Crystallographic Data Centre and Fachinformationszentrum Karlsruhe Access Structures service.

## Supporting information

As a service to our authors and readers, this journal provides supporting information supplied by the authors. Such materials are peer reviewed and may be re‐organized for online delivery, but are not copy‐edited or typeset. Technical support issues arising from supporting information (other than missing files) should be addressed to the authors.

Supporting Information
