# Supplementary material for: Heterodinuclear Zn(II), Mg(II) or Co(III) with Na(I) Catalysts for Carbon Dioxide and Cyclohexene Oxide Ring Opening Copolymerizations
Source: Chemistry. 2021 Jul 9;27(47):12224–31. doi: 10.1002/chem.202101140 (PMC8456860; doi:10.1002/chem.202101140)
Supplement: Supplementary file 1 — Supporting Information [file CHEM-27-12224-s001.pdf]

Chemistry–A European Journal

Supporting Information

Heterodinuclear Zn(II), Mg(II) or Co(III) with Na(I) Catalysts for Carbon Dioxide and Cyclohexene Oxide Ring Opening Copolymerizations

Wouter Lindeboom, Duncan A. X. Fraser, Christopher B. Durr, and Charlotte K. Williams*

Contents

Table S1. ^{1}H Chemical Shifts for the reactant and products of CHO/CO_2 copolymerization in $CDCl_3$ ... 3
Figure S1. ^{1}H NMR spectrum of **1** (d_2-TCE, 298K). ... 7
Figure S2. ^{1}H NMR spectrum of **2** (d_2-TCE, 398K). ... 7
Figure S3. ^{1}H NMR spectrum of **3** (d_2-TCE, 298K). ... 8
Figure S4. ^{1}H NMR spectrum of **4** (d_2-TCE, 328K). ... 8
Figure S5. ^{1}H NMR spectrum of **5** ($CDCl_3$, 298K). ... 9
Figure S6. ^{1}H NMR spectrum of **6** ($CDCl_3$, 298K). ... 9
Figure S7. ^{1}H NMR spectrum of **7** ($CDCl_3$, 298K). ... 10
Figure S8. ^{1}H NMR spectrum of **8** ($CDCl_3$, 298K). ... 10
Figure S9. ^{1}H NMR spectrum of **9** ($CDCl_3$, 298K). ... 11
Figure S10. ^{1}H NMR spectrum of **10** ($CDCl_3$, 298K). ... 11
Figure S11. ^{1}H NMR spectrum of **11** (d_2-TCE, 298K). ... 12
Figure S12. ^{13}C NMR spectrum of **1** (d_2-TCE, 298K). ... 12
Figure S13. ^{13}C NMR spectrum of **3** (d_2-TCE, 298K). ... 13
Figure S14. ^{13}C NMR spectrum of **4** (d_2-TCE, 328K). ... 13
Figure S15. ^{13}C NMR of **5** ($CDCl_3$, 298K). ... 14
Figure S16. ^{13}C NMR of **6** ($CDCl_3$, 298K). ... 14
Figure S17. ^{13}C NMR of **7** ($CDCl_3$, 298K). ... 15
Figure S18. ^{13}C NMR of **8** ($CDCl_3$, 298K) (solid state). ... 15
Figure S19. ^{13}C NMR of **9** ($CDCl_3$, 298K) (solid state). ... 16
Figure S20. ^{13}C NMR of **10** ($CDCl_3$, 298K) (solid state). ... 16
Figure S21. ^{13}C NMR of **11** (d_2-TCE, 298K). ... 17
Figure S22. 2D HSQC NMR of **1** (d_2-TCE, 298K). ... 17
Figure S23. 2D HMBC NMR of **1** (d_2-TCE, 298K). ... 18
Figure S24. 2D HSQC NMR of **2** (d_2-TCE, 398K). ... 18
Figure S25. 2D HMBC NMR of **2** (d_2-TCE, 398K). ... 19
Figure S26. 2D COSY NMR of **3** (d_2-TCE, 298K). ... 19
Figure S27. 2D HSQC NMR of **3** (d_2-TCE, 298K). ... 20
Figure S28. 2D HMBC NMR of **3** (d_2-TCE, 298K). ... 20
Figure S29. 2D HSQC NMR of **4** (d_2-TCE, 328K). ... 21
Figure S30. 2D HMBC NMR of **4** (d_2-TCE, 328K). ... 21
Figure S31. 2D COSY NMR of **5** ($CDCl_3$, 298K). ... 22
Figure S32. HSQC NMR of **5** ($CDCl_3$, 298K). ... 22
Figure S33. HMBC NMR of **5** ($CDCl_3$, 298K). ... 23
Figure S34. 2D COSY NMR of **6** ($CDCl_3$, 298K). ... 23
Figure S35. 2D HSQC NMR of **6** ($CDCl_3$, 298K). ... 24
Figure S36. 2D HMBC NMR of **6** ($CDCl_3$, 298K). ... 24
Figure S37. 2D COSY NMR of **7** ($CDCl_3$, 298K). ... 25
Figure S38. 2D HSQC NMR of **7** ($CDCl_3$, 298K). ... 25
Figure S39. 2D HMBC NMR of **7** ($CDCl_3$, 298K). ... 26
Figure S40. 2D COSY NMR of **8** ($CDCl_3$, 298K). ... 26
Figure S41. 2D HSQC NMR of **8** ($CDCl_3$, 298K). ... 27
Figure S42. 2D HMBC NMR of **8** ($CDCl_3$, 298K). ... 27
Figure S43. 2D COSY NMR of **9** ($CDCl_3$, 298K). ... 28
Figure S44. 2D HSQC NMR of **9** ($CDCl_3$, 298K). ... 28
Figure S45. 2D HMBC NMR of **9** ($CDCl_3$, 298K). ... 29
Figure S46. 2D COSY NMR of **10** ($CDCl_3$, 298K). ... 29
Figure S47. 2D HSQC NMR of **10** ($CDCl_3$, 298K). ... 30
Figure S48. 2D HMBC NMR of **10** ($CDCl_3$, 298K). ... 30
Figure S49. 2D HSQC NMR of **11** (d_2-TCE, 298K). ... 31
Figure S50. 2D HMBC NMR of **11** (d_2-TCE, 298K). ... 31
Figure S51. Molecular structures for **7**, fully labeled. ... 32
Figure S52. ESI-Q Mass spectrum for **10**, with assignment of principal fragmentation ions. ... 32
Figure S53. ESI-Q Mass spectrum for **7**, with assignment of principal fragmentation ions. ... 33
Figure S54. IR spectra of complexes a) **1**, b) **2**, c) **3** and d) **4**. ... 33
Figure S55. IR spectra of complexes a) **5**, b) **6** and c) **7**. ... 34
Figure S56. IR spectra of complexes a) **8**, b) **9**, c) **10** and d) **11**. ... 34
Figure S57. Molecular structures for **1**, fully labeled. ... 35
Figure S58. Molecular structures **1**(EtOH), fully labeled. ... 35
Figure S59. Molecular structures for **3**, fully labeled. ... 36
Figure S60. Molecular structures for **11**, fully labeled. ... 36
Table S2. Selected bond lengths from **1**. ... 37
Table S3. Selected bond lengths from **1**(EtOH). ... 37
Table S4. Selected bond lengths from **3**. ... 37
Table S5. Selected bond lengths from **7**. ... 37
Table S6. Selected bond lengths from **11**. ... 38
Table S7. Selected Crystallographic details. ... 38

Figure S61. Chemical structure of literature catalysts $L_\mathbf{a}MgZn$, $L_\mathbf{a}MgCo$, $L_\mathbf{b}Zn_3Ce$ and $L_\mathbf{c}Co^{III}(X)/^{n}Bu_4NX$. **39**
Figure S62. GPC data for entries 1 – 4 of Table 1. ....**39**
Figure S63. GPC data for entries 6 – 7 of Table 1. ....**40**
Figure S64. GPC data for entries 9 – 12 of Table 1. ....**40**
Figure S65. Copolymerization kinetic data (a) Absorbance vs. time plot for the three PCHC wavenumbers (every 30th data point displayed). (b) Normalised conversion vs. time plot for the three PCHC wavenumbers (every 30th data point displayed). (c) ReactIR spectra recorded at the start and finish of the copolymerization. Three peaks associated with PCHC were analysed: 988, 1160 and 1330 cm^{-1}. (d) Logarithm of epoxide concentration vs. time for the three PCHC wavenumbers. k_{obs} (s^{-1}) values are reported and the average value used in subsequent analysis. **41**
Table S8. Variable Temperature Polymerisation Data for Selected Catalysts. **41**
Figure S66. GPC data for entries 1 – 3 of Table S8. ....**42**
Figure S67. GPC data for entries 4 – 6 of Table S8. ....**43**
Figure S68. GPC data for entries 7– 9 of Table S8. ....**43**
References **44**

Experimental Section

Low pressure copolymerizations: For 1 bar CO_2 polymerzations, an ampoule was charged with catalyst, trans-1,2-cyclohexanediol (10 equiv.) and cyclohexene oxide (1.5 mL, 14.8 mmol) in a nitrogen glovebox. The headspace was purged with five partial vacuum/CO_2 cycles performed on a triple manifold Young tap Schlenk line (N_2, CO_2, and vacuum) before the solution was heated to the desired temperature. The reaction mixture was left under a dynamic CO_2 atmosphere of 1 bar and stirred at 1000 rpm for the reaction duration. The reaction was quenched by exposure to air and analysed by ^{1}H NMR spectroscopy and GPC. Research-grade carbon dioxide (BOC, 99.99 %) was used and dried through a Drierite column and two additional drying columns (Micro Torr, Model number: MC1-804FV) in series before use.

High pressure polymerizations: High pressure polymerizations (10-40 bar) were performed in a 25 mL Parr 5500 HP Compact Reactor. The reactor was charged with catalyst, trans-1,2-cyclohexanediol (10 equiv.) and cyclohexene oxide (6.1 mL, 60.3 mmol) in a nitrogen glovebox. The reactor was heated to the desired temperature before being pressurized to the desired pressure with carbon dioxide. The reaction was stirred, under a static pressure of CO_2, for the reaction duration and was quenched by cooling and placing the reactor in an ice bath prior to the pressure being released. The crude reaction mixture was analysed by ^{1}H NMR spectroscopy and GPC. For polymerizations above 1 bar, research grade carbon dioxide (BOC, 99.99 %) was used and dried through two MicroTorr purifier columns at point of use.

Materials: Unless otherwise specified, all chemicals were purchased from commercial suppliers (Sigma Aldrich, Acros, Alfa Aesar and Fischer) and used as received. Anhydrous solvents were obtained from a solvent purification system, degassed by several freeze-pump-thaw cycles and subsequently dried over 3 Å molecular sieves. Cyclohexene oxide (CHO; 98%, Sigma Aldrich) was first stirred over calcium hydride for 48 h, then fractionally distilled and stored under nitrogen.

Nuclear Magnetic Resonance Spectroscopy: For ^{1}H NMR, solution state ^{13}C{^{1}H} NMR and all 2D NMR measurements a Bruker Avance III HD nanobay NMR equipped with a 9.4T magnet (^{1}H 400 MHz, ^{13}C 100 MHz) NMR spectrometer was used. For all solid state ^{13}C{^{1}H} NMR measurements a Bruker Avance III HD Solid state NMR spectrometer equipped with a 9.4T magnet (^{1}H 400 MHz, ^{13}C 100 MHz) was used.

Polymer Characterization: ^{1}H-NMR spectroscopy was used to determine the conversion and selectivity of the polymerization reactions from the relative integrals of the polymer, monomer and side-products signals. The chemical shifts used in the copolymerization of CO_2 and CHO are shown in Table S1.

Table S1. ^{1}H Chemical Shifts for the reactant and products of CHO/CO_2 copolymerization in $CDCl_3$.

**Component**	CHO	PCHC Backbone	PCHC End Groups	Cyclic Carbonate	Polyether
**Chemical shift (ppm)**	3.05	4.65	4.35, 3.50	4.00, 4.68	3.45

The polymerization conversion was calculated from the relative integrals of the products against monomers (1). The selectivity for CO_2 was calculated by comparison of the integrals of polycarbonate and cyclic carbonate against polyether resonances (2). Polycarbonate selectivity was calculated through relative integrals of all polycarbonate polymer peaks against cyclic by-products and polyether (3).

$$Conv.(\%) = 100 \times \frac{\int H_{polycarbonate} + \int H_{cyclic\ carbonate} + \int H_{polyether}}{\int H_{polycarbonate} + \int H_{cyclic\ carbonate} + \int H_{polyether} + \int H_{CHO}} \quad (1)$$

$$CO_2\ selec.(\%) = 100 \times \frac{\int H_{polycarbonate} + \int H_{cyclic\ carbonate}}{\int H_{polycarbonate} + \int H_{cyclic\ carbonate} + \int H_{polyether}} \quad (2)$$

$$Polycarb.selec.(\%) = 100 \times \frac{\int H_{polycarbonate}}{\int H_{polycarbonate} + \int H_{cyclic\ carbonate} + \int H_{polyether}} \quad (3)$$

Mass spectrometry: All the mass spectrometry was conducted by the National Mass Spectrometry Facility at Swansea University.

Gel Permeation Chromatography: Gel Permeation Chromatography (GPC) was carried out on a two mixed bed PSS SDV linear S column in series, with THF as the eluent at a flow rate of 1mL/min on a Shimadzu LC20AD instrument at 40 °C. Polymer Molar Mass (M_n) was determined by comparison against polystyrene standards. The polymer samples were dissolved in HPLC-grade THF (10 mg/mL) and filtered through a 0.20 µm porous filter frit prior to analysis.

Single Crystal X-ray Diffraction Experiments: The crystal structures were collected and solved by Dr. Chris Durr. The general procedure for these X-ray diffraction experiments has been described previously.[1] Samples were isolated and immersed in fluorinated oil (Fomblin ® Y). Single crystals suitable for diffraction were mounted on MiTeGen MicroMounts and cooled to 150 K, under a stream of nitrogen, with an Oxford Cryosystems Cryostream. Data was collected with an Oxford Diffraction Supernova Diffractometer using Cu Kα (λ = 1.5417 Å) radiation. Data was processed using CrysAlisPRO[2] and structural solution was performed with SHELXT[3] followed by refinement with SHELXL-14,[3] which was incorporated within the WinGX graphical user interface.[4]

All structures have been registered with the Cambridge Structural Database. CCDC 2073147-2073150 contains the supplementary crystallographic data for this paper. The data can be obtained free of charge from The Cambridge Crystallographic Data Centre via www.ccdc.cam.ac.uk/structures.

1 contained highly disordered lattice solvent. After attempting to model the solvent, the SQUEEZE program, as incorporated into PLATON, was utilized.[5] ,6 A twin law of (1 1 0) (0 -1 0) (0 0 -1) was also applied to the structure.

1(EtOH) and **3** showed ligand disorder that was successfully modelled. In each case part of the crown ether group was modelled in two positions using distance and thermal restraints to ensure a stable refinement. The relative occupancies of each part were refined using a free variable.

ATR-IR Spectroscopy Measurements: *In situ* ATR-IR measurements were performed on a Mettler-Toledo ReactIR ic.10 spectrometer equipped with a MCT detector and a silver halide DiComp probe. In most experiments an IR spectrum was measured every minute over the course of the reaction. The change in absorbance intensity over time (from start to finish) was normalised and correlated to conversion, by removal of an aliquot at the end of the experiment to determine the conversion by ^{1}H NMR spectroscopy.

Synthesis of LH_2: To a suspension of NaH (2.19 g, 91.10 mmol), in DMSO (10 mL), a solution of 2,3-dihydroxybenzaldehyde (5.72 g, 41.40 mmol), in DMSO (30 mL), was added, dropwise, over the course of 2 h. Triethylene glycol ditosylate (9.50 g, 21.20 mmol) was then added in one portion and the mixture stirred for 48 h, under N_2. Water (300 mL) was added, the mixture was washed with $CHCl_3$ (100 mL) and the organic layer was discarded. The aqueous layer was then acidified to pH 1 (using 6M HCl) and the product extracted with chloroform (3 x 50 mL). The organic layer was washed with HCl (1M, 100 mL) and then dried ($MgSO_4$). The volatiles were removed under vacuum and the crude product was further purified by column chromatography (silica gel, chloroform: pentane: acetone, 65: 40: 10) to give a pale-yellow solid (3.56 g, 9.12 mmol, 43%). Spectroscopic data is in agreement with literature values.[14a, 14g]

Synthesis of 1: A methanol (350 mL) solution of **LH_2** (300 mg, 0.77 mmol) and sodium acetate (63.2 mg, 0.77 mmol) was refluxed and a methanol (250 mL) solution of 2,2-dimethylpropane-1,3-diamine (78.6 mg, 0.77 mmol) was added, dropwise, over the course of 3 h. The solution was left to cool to room temperature, before the $Zn(OAc)_2·2(H_2O)$ (169 mg, 0.77 mmol) was added and left to stir for 1 h. The solvent was removed, under reduced pressure, to obtain a yellow glassy solid as the crude. This was triturated with ethanol (50 mL) and dichloromethane (50 mL) and dried under vacuum at 40 °C to give a pure product (343 mg, 0.57 mmol, 74%).

^{1}H NMR (400 MHz, $C_2D_2Cl_4$, 298 K). δ (ppm): 7.87 (s, 2H, –**H**C=N–), 6.85 (dd, 2H, Ar-**H**$_{meta}$, $^3J_{H-H}$ = 7.6 Hz, $^4J_{H-H}$ = 1.8 Hz), 6.81 (dd, 2H, Ar-**H**$_{meta}$, $^3J_{H-H}$ = 7.6 Hz, $^4J_{H-H}$ = 1.8 Hz), 6.42 (t, 2H, Ar-**H**$_{para}$,$^3J_{H-H}$ = 7.7 Hz), 4.26-3.64 (m, 14H, –**H$_2$**C–N=, –O–C**H$_2$**–), 3.09 (d, 2H, –**H$_2$**C–N=, $^3J_{H-H}$ = 12.0 Hz), 1.89 (s, 3H, **H$_3$**C–C(O)O), 1.08 (s, 3H, **H$_3$**C–C–), 0.82 (s, 3H, **H$_3$**C–C–).

^{13}C NMR (100 MHz, $C_2D_2Cl_4$, 298 K). δ (ppm): 177.5 (–**C**(O)O), 168.6 (–H**C**=N–), 162.1 (Ar–**C**$_{ortho}$), 150.5 (Ar–**C**$_{ortho}$), 128.3 (Ar–**C**$_{meta)}$, 119.06 (Ar–**C**$_{meta)}$, 117.46 (Ar–**C**$_{ipso}$), 111.6 (Ar–**C**$_{para}$), 74.65 (–H$_2$**C**–N=), 68.93 (–H$_2$**C**–N=, –O–**C**H$_2$–), 68.48 (–H$_2$**C**–N=, –O–**C**H$_2$–), 68.40 (–H$_2$**C**–N=, –O–**C**H$_2$–), 36.13 ((H$_3$C)$_2$–**C**–), 27.28 (H$_3$**C**–C–), 24.73 (H$_3$**C**–C(O)O), 22.29 (H$_3$**C**–C–).

Anal calcd (found) for $C_{27}H_{33}N_2NaO_8Zn$: C, 53.88 (53.81); H, 5.53 (5.62); N 4.65 (4.60).

Synthesis of 2: A methanol (350 mL) solution of **LH_2** (200.0 mg, 0.51 mmol) and sodium acetate (42.1 mg, 0.51 mmol) was refluxed and a methanol (250 mL) solution of 2,2-dimethylpropane-1,3-diamine (52.3 mg, 0.51 mmol) was added, dropwise, over the course of 3 h. The solution was left to cool to room temperature, before the $Mg(OAc)_2·4(H_2O)$ (110 mg, 0.51 mmol) was added and left to stir for 1 h. The solvent was removed under reduced pressure to obtain a yellow glassy solid as the crude. The product was triturated with ethanol (50 mL) and dichloromethane (50 mL). and dried under vacuum at (40 °C) to obtain the product (179 mg, 0.32 mmol, 63%).

^{1}H NMR (400 MHz, $C_2D_2Cl_4$, 398 K). δ (ppm): 8.05 (s, 2H, –**H**C=N–), 6.88 (m, 4H, Ar-**H**$_{meta}$), 6.46 (t, 2H, Ar-**H**$_{para}$,$^3J_{H-H}$ = 7.5 Hz), 4.25-3.61 (m, 16 H, –**H$_2$**C–N=, –O–C**H$_2$**–), 1.99 (s, 6H, **H$_3$**C–C(O)O), 1.01 (s, 6H, **H$_3$**C–C–).

Anal calcd (found) for $C_{27}H_{33}MgN_2NaO_8$: C, 57.82 (55.15); H, 5.93 (6.21); N 4.99 (4.40).

Synthesis of 3: Under a nitrogen atmosphere, to a solution of **LH_2** (400 mg, 1.03 mmol) and sodium acetate (84.5 mg, 1.03 mmol), in methanol (500 mL), a methanol (250 mL) solution of 2,2-dimethylpropane-1,3-diamine (105.25 mg, 1.03 mmol) was added, dropwise, over the course of 3 h, under reflux. The solution was left to cool to room temperature before $Co(OAc)_2·4(H_2O)$ (256.6 mg, 1.03 mmol) was added and the solution left to stir overnight. It was further stirred under ambient conditions for 24 h. The solvent was removed under reduced pressure to obtain a dark brown glassy solid. This was dissolved in acetonitrile (30 mL) and diluted with diethyl ether (300 mL). The precipitate was isolated by filtration and triturated once with chloroform (50 mL) to obtain the product (533.0 mg, 0.81 mmol, 79%).

^{1}H NMR (400 MHz, $C_2D_2Cl_2$, 298 K). δ (ppm): 7.24 (s, 2H, –**H**C=N–), 6.82 (dt, 2H, Ar-**H**$_{meta}$, $^3J_{H-H}$ = 21.4, $^4J_{H-H}$ = 5.3 Hz), 6.55-6.44 (mm, 2H, Ar-**H**$_{meta}$), 4.30-3.69 (m, 12H, –O–C**H$_2$**–), 3.41 (s, 4H, –**H$_2$**C–N=), 1.47 (s, 6H, **H$_3$**C–C(O)O), 1.19 (s, 6H, **H$_3$**C–C–).

^{13}C NMR (100 MHz, $C_2D_2Cl_2$, 298 K). δ(ppm): 180.8 (–**C**(O)O), 166.2 (–H**C**=N–), 157.1 (Ar–**C**$_{ortho}$), 151.9 (Ar–**C**$_{ortho}$), 126.3 (Ar–**C**$_{meta}$), 122.6 (Ar–**C**$_{ipso}$), 117.25 (Ar–**C**$_{meta}$), 114.2 (Ar–**C**$_{para}$), 71.22 (=N–**C**H$_2$–), 68.77 (–O–**C**H$_2$–), 68.08 (–O–**C**H$_2$–), 35.07 ((H$_3$C)$_2$–**C**–(CH$_2$)$_2$), 25.13(H$_3$**C**–C(O)O, H$_3$**C**–C–), 24.62 (H$_3$**C**–C(O)O), H$_3$**C**–C–).

Anal calcd (found) for $C_{27}H_{33}CoN_2NaO_8$: C, 54.46 (53.57); H, 5.59 (5.38); N 4.70 (4.73).

Synthesis of 4: A methanol (350 mL) solution of **LH_2** (300 mg, 0.77 mmol) and $Mg(OAc)_2·4(H_2O)$ (165 mg, 0.77 mmol) was refluxed and a methanol (250 mL) solution of 2,2-dimethylpropane-1,3-diamine (78.5 mg, 0.77 mmol) was added, dropwise, over the course of 3 h. The solution was left to cool to room temperature, before the $Zn(OAc)_2·2(H_2O)$ (169 mg, 0.77 mmol) was added and left to stir for 1 h. The solvent was removed, under reduced pressure, to obtain a yellow glassy solid as the crude. This was

triturated with ethanol (50 mL) and dichloromethane (50 mL) and dried under vacuum at 40 °C to give a pure product (359 mg, 0.554 mmol, 72%).

^{1}H NMR (400 MHz, $C_2D_2Cl_4$, 328 K). δ (ppm): 8.00 (s, 2H, –**H**C=N–), 6.80 (dd, 4H, Ar-**H**$_{meta}$, $^{3}J_{H-H}$ = 18.5 Hz, $^{4}J_{H-H}$ = 7.8 Hz), 6.59 (t, 2H, Ar-**H**$_{para}$,$^{3}J_{H-H}$ = 7.9 Hz), 4.32 (d, 2H, –**H$_2$**C–N=, $^{2}J_{H-H}$ = 11.9 Hz), 4.21 (s, 4H, –O–C**H$_2$**–), 3.98-3.60 (m, 8H, –O–C**H$_2$**–), 3.13 (d, 2H, –**H$_2$**C–N=, $^{2}J_{H-H}$ = 11.9 Hz), 2.00 (s, 6H, **H$_3$**C–C(O)O), 1.13 (s, 3H, **H$_3$**C–C–), 0.85 (s, 3H, **H$_3$**C–C–).

^{13}C NMR (100 MHz, $C_2D_2Cl_4$, 328 K). δ (ppm): 168.2 (–H**C**=N–), 157.9 (Ar–**C**$_{ortho}$), 148.9 (Ar–**C**$_{ortho}$), 126.5 (Ar–**C**$_{meta}$), 118.3 (Ar–**C**$_{ipso}$), 113.4 (Ar–**C**$_{para}$), 112.4 (Ar–**C**$_{meta}$), 74.42 (–H_2**C**–N=), 69.36 (–O–**C**H_2–), 68.10 (–O–**C**H_2–), 66.66 (–O–**C**H_2–), 57.90 (–O–**C**H_2–), 35.56 ((H_3C)$_2$–**C**–), 26.69 (H_3**C**–C–), 21.51 (H_3**C**–C–, H_3**C**–C(O)O).

Anal calcd (found) for $C_{29}H_{36}MgN_2O_{10}Zn$: C, 52.59 (52.48); H, 5.48 (5.29); N 4.23 (4.25).

Synthesis of 5: A methanol (350 mL) solution of **LH$_2$** (300 mg, 0.77 mmol) and sodium acetate (63.1 mg, 0.77 mmol) was refluxed and a methanol (250 mL) solution of ethylene diamine (46.2 mg, 0.77 mmol) was added, dropwise, over the course of 3 h. The solution was left to cool to room temperature, before the $Zn(OAc)_2{\cdot}2(H_2O)$ (169 mg, 0.77 mmol) was added and left to stir for 1 h. The solvent was removed, under reduced pressure, to obtain a pale yellow glassy solid as the crude. This was triturated with dichloromethane (50 mL) and dried under vacuum at 60 °C to give a pure product (298 mg, 0.53 mmol, 69%).

^{1}H NMR (400 MHz, $CDCl_3$, 298 K). δ(ppm): 8.29 (2H, s, –**H**C=N–), 6.81 (4H, m, Ar–**H**$_{meta}$), 6.41 (2H, m, Ar–**H**$_{para}$), 4.22-3.60 (16H, m, , –O–C**H$_2$**–, , =N–C**H$_2$**–), 1.81 (3H, s, **H$_3$**C–C(O)O).

^{13}C NMR (100 MHz, $CDCl_3$, 298 K). δ(ppm): 177.0 (–**C**(O)O), 167.6 (–H**C**=N–), 150.8 (Ar–**C**$_{ortho}$–O–CH_2), 128.0 (Ar–**C**$_{meta}$), 120.0 (Ar–**C**$_{ipso}$), 117.7 (Ar–**C**$_{meta}$), 112.0 (Ar–**C**$_{para}$), 70.1 (–O–**C**H_2–), 69.8 (–O–**C**H_2–), 67.8 (–O–**C**H_2–), 56.1 (=N–**C**H_2–), 23.6 (H_3**C**–C(O)O).

Anal. Calcd for $C_{28}H_{27}N_2NaO_8Zn$: C, 51.49; H, 4.86; N, 5.00. Found: C, 51.80; H, 4.97; N, 5.22.

Synthesis of 6: Followed the general procedure outlined for **2$_{Zn-Na}$**, replacing $Zn(OAc)_2{\cdot}2(H_2O)$ with $Mg(OAc)_2{\cdot}4(H_2O)$ (165 mg, 0.79 mmol). The solvent was removed under reduced pressure to obtain a yellow glassy solid as the crude. The product was recrystallized from methanol/diethyl ether mixture (1:10) at -20 °C. The crystals were washed with pentane (10 mL) and triturated with chloroform (20 mL) and dried under vacuum at (40 °C) to obtain the product (59.1 mg, 0.11 mmol, 15%).

^{1}H NMR (400 MHz, $CDCl_3$, 298 K). δ(ppm): 8.20 (2H, s, –**H**C=N–), 6.79 (4H, m, Ar–**H**$_{meta}$), 6.33 (2H, t, Ar–**H**$_{para}$, $^{3}J_{H-H}$ = 7.7 Hz), 4.38-3.42 (16H, m, –O–C**H$_2$**–, =N–C**H$_2$**–), 1.75 (3H, s, **H$_3$**C–C(O)O).

^{13}C NMR (100 MHz, $CDCl_3$, 298 K). δ(ppm): 179.2 (–**C**(O)O), 167.8 (–H**C**=N–), 161.0 (Ar–**C**$_{ortho}$–HC=N–), 150.6 (Ar–**C**$_{ortho}$–O–CH_2), 128.3 (Ar–**C**$_{meta}$), 121.7 (Ar–**C**$_{ipso}$), 118.5 (Ar–**C**$_{meta}$), 111.8 (Ar–**C**$_{para}$), 70.6 (–O–**C**H_2–), 69.9 (–O–**C**H_2–), 67.9 (–O–**C**H_2–), 57.2 (=N–**C**H_2–), 24.2 (H_3**C**–C(O)O).

Synthesis of 7: Under a nitrogen atmosphere, to a solution of **LH$_2$** (400 mg, 1.03 mmol) and sodium acetate (84.1 mg, 1.03 mmol), in methanol (500 mL), a methanol (250 mL) solution of ethylene diamine (61.6 mg, 1.03 mmol) was added, dropwise, over the course of 3 h, under reflux. The solution was left to cool to room temperature before $Co(OAc)_2{\cdot}4(H_2O)$ (256 mg, 1.03 mmol) was added and the solution left to stir overnight. It was further stirred under ambient conditions for 24 h. The solvent was removed under reduced pressure to obtain a dark brown glassy solid. This was dissolved in acetonitrile (30 mL) and diluted with diethyl ether (300 mL). The precipitate was isolated by filtration and triturated once with chloroform (50 mL) to obtain the product (250 mg, 0.41 mmol, 40%).

^{1}H NMR (400 MHz, $CDCl_3$, 298 K). δ(ppm): 7.76 (2H, s, –**H**C=N–), 6.88 (2H, dd, Ar–**H**$_{meta}$, $^{3}J_{H-H}$ = 7.95 Hz, $^{4}J_{H-H}$ = 1.57 Hz), 6.79 (2H, dd, Ar–**H**$_{meta}$, $^{3}J_{H-H}$ = 7.59 Hz, $^{4}J_{H-H}$ = 1.58 Hz), 6.51 (2H, t, Ar–**H**$_{para}$, $^{3}J_{H-H}$ = 7.76 Hz), 4.35, 4.20, 3.94, 3.85 (16H, m, –O–C**H$_2$**–, =N–C**H$_2$**–), 1.38 (3H, s, **H$_3$**C–C(O)O).

^{13}C NMR (100 MHz, $CDCl_3$, 298 K). δ(ppm): 179.3 (–**C**(O)O), 164.6 (–H**C**=N–), 156.5 (Ar–**C**$_{ortho}$–HC=N–), 151.7 (Ar–**C**$_{ortho}$–O–CH_2), 126.2 (Ar–**C**$_{meta}$), 119.0 (Ar–**C**$_{ipso}$), 114.7 (Ar–**C**$_{meta}$), 112.7 (Ar–**C**$_{para}$), 69.0 (–O–**C**H_2–), 68.8 (–O–**C**H_2–), 67.1 (–O–**C**H_2–), 58.9 (=N–**C**H_2–), 24.7 (H_3**C**–C(O)O).

HRMS (ESI/FTMS) m/z: [**7** - OAc]$^{+}$ Calcd for $C_{24}H_{27}CoN_2NaO_8$ 553.0992; Found 553.0977.

Anal. Calcd for $C_{26}H_{30}CoNaN_2O_{10}$: Calculated; C, 51.0; H, 4.9; N, 4.6 %. Found: C, 50.7; H, 4.8; N, 4.4.

Synthesis of 8: A methanol (350 mL) solution of **LH$_2$** (300 mg, 0.77 mmol) and sodium acetate (63.1 mg, 0.77 mmol) was refluxed and a methanol (250 mL) solution of *O*-phenylenediamine (83.2 mg, 0.77 mmol) was added, dropwise, over the course of 3 h. The solution was left to cool to room temperature, before the $Zn(OAc)_2{\cdot}2(H_2O)$ (169 mg, 0.77 mmol) was added and left to stir for 1 h. The solvent was removed, under reduced pressure, leaving a yellow-orange powder contaminated by acetic acid. The solid crude was triturated with methanol (100 mL) and washed with diethyl ether (50 mL) to remove the acid by-product to give the target compound (0.53 mmol, 320 mg, 68%).

^{1}H NMR (400 MHz, $CDCl_3$, 298 K). δ(ppm): 8.68 (2H, s, –**H**C=N–), 7.57 (2H, m, Ar–**H**$_{ortho'}$), 7.31 (2H, m, Ar–**H**$_{meta'}$), 6.96 (2H, dd, Ar–**H**$_{meta}$, $^{3}J_{H-H}$ = 8.02 Hz, $^{4}J_{H-H}$ = 1.69 Hz), 6.90 (2H, dd, Ar–**H**$_{meta}$, $^{3}J_{H-H}$ = 7.60 Hz, $^{4}J_{H-H}$ = 1.73 Hz), 6.46 (2H, t, Ar–**H**$_{para}$, $^{3}J_{H-H}$ = 7.75 Hz), 4.34-3.76 (12H, m, –O–C**H$_2$**–), 1.79 (3H, s, **H$_3$**C–C(O)O).

$^{13}C\{^1H\}$ CP-MAS (100 MHz, 298 K). δ(ppm): 178.0 (–**C**(O)O), 162.8, 161.5, 159.6, 151.3 (Ar–**C**$_{ortho}$–O–CH$_2$), 139.3 (Ar–**C**$_{ipso}$), 127.7, 125.5 (Ar–**C**$_{meta'}$, Ar–**C**$_{meta}$), 118.6, 115.7, 110.1 (Ar–**C**$_{para}$, Ar–**C**$_{meta,}$ Ar–**C**$_{ortho'}$), 73.3 (–O–**C**H$_2$–), 69.3 (–O–**C**H$_2$–), 67.5 (–O–**C**H$_2$–), 65.8 –O–**C**H$_2$–), 24.5 (H$_3$**C–**C(O)O).

Anal. Calcd for $C_{28}H_{27}N_2NaO_8Zn$: C, 55.32; H, 4.48; N, 4.61. Found: C, 55.19; H, 4.62; N, 4.49.

Synthesis of 9: Followed the general procedure outlined for **3**$_{\mathbf{Zn\text{-}Na}}$, replacing $Zn(OAc)_2{\cdot}2(H_2O)$ with $Mg(OAc)_2{\cdot}4(H_2O)$ (164.9 mg, 0.77 mmol). The crude product was triturated with methanol (50 mL), chloroform (2 x 50 mL) and washed with diethyl ether (20 mL) then stirred in excess ethanol (100 mL) overnight. Ethanol was removed under reduced pressure to give **2A** (0.25 mmol, 144 mg, 33%).

^1H NMR (400 MHz, $CDCl_3$, 298 K). δ(ppm): 8.68 (2H, s, –**H**C=N–), 7.57 (2H, m, Ar–**H**$_{ortho'}$), 7.32 (2H, m, Ar–**H**$_{meta'}$), 6.96 (2H, dd, Ar–**H**$_{meta}$, $^3J_{H\text{-}H}$ = 8.02 Hz, $^4J_{H\text{-}H}$ = 1.69 Hz), 6.91 (2H, dd, Ar–**H**$_{meta}$, $^3J_{H\text{-}H}$ = 7.60 Hz, $^4J_{H\text{-}H}$ = 1.73 Hz), 6.46 (2H, t, Ar–**H**$_{para}$, $^3J_{H\text{-}H}$ = 7.75 Hz), 4.33-3.76 (12H, m, –O–C**H**$_2$–), 1.79 (3H, s, **H**$_3$C–C(O)O).

$^{13}C\{^1H\}$ CP-MAS (100 MHz, 298K). δ(ppm): 177.0 (–**C**(O)O), 161.5, 160.2 (Ar–**C**$_{ortho}$–HC=N–, H**C**=N–), 151.2 (Ar–**C**$_{ortho}$–O–CH$_2$), 141.2 (Ar–**C**$_{ipso'}$), 127.7, 125.6 (Ar–**C**$_{ortho'}$, Ar–**C**$_{meta}$), 120.4 (Ar–**C**$_{ipso}$), 117.9, 115.5, 111.6 (Ar–**C**$_{para}$, Ar–**C**$_{meta}$, Ar–**C**$_{meta'}$), 73.1 (–O–**C**H$_2$–), 69.3 (–O–**C**H$_2$–), 67.4 (–O–**C**H$_2$–), 65.9 (–O–**C**H$_2$–), 24.6 (H$_3$**C**–C(O)O).

Anal. Calcd for $C_{28}H_{27}N_2NaO_8Mg$: C, 59.33; H, 4.80; N, 4.94. Found: C, 59.14; H, 4.76; N, 4.83.

Synthesis of 10: Under a nitrogen atmosphere, to a solution of **LH$_2$** (400 mg, 1.03 mmol) and sodium acetate (84.1 mg, 1.03 mmol), in methanol (500 mL), a methanol (250 mL) solution of *O*-phenylenediamine (111 mg, 1.03 mmol) was added, dropwise, over the course of 3 h, under reflux. The solution was left to cool to room temperature before $Co(OAc)_2{\cdot}4(H_2O)$ (256 mg, 1.03 mmol) was added and the solution left to stir overnight. It was further stirred under ambient conditions for 24 h. The solvent was removed, under reduced pressure, to obtain a dark brown glassy powder. This was dissolved in acetonitrile (50 mL) and diluted with diethyl ether (400 mL), precipitating a black solid which was removed by filtration and triturated once with chloroform (100 mL) obtaining the pure product (291 mg, 0.44 mmol, 43%).

^1H NMR (400 MHz, $CDCl_3$, 298 K). δ(ppm): 8.23 (2H, s, –**H**C=N–), 8.00 (2H, m, Ar–**H**$_{ortho'}$), 7.38 (2H, m, Ar–**H**$_{meta'}$), 7.04 (2H, d, Ar–**H**$_{meta}$, $^3J_{H\text{-}H}$ = 7.96 Hz), 6.82 (2H, d, Ar–**H**$_{meta}$, $^3J_{H\text{-}H}$ = 7.55 Hz), 6.51 (2H, t, Ar–**H**$_{para}$, $^3J_{H\text{-}H}$ = 7.81 Hz), 4.23 (4H, m, –O–C**H**$_2$–), 3.85 (4H, m, –O–C**H**$_2$–), 3.65 (4H, s, –O–C**H**$_2$–), 1.38 (3H, s, **H**$_3$C–C(O)O).

$^{13}C\{^1H\}$ CP-MAS (100MHz, 298K). δ(ppm): 181.7 (–**C**(O)O), 178.1 (–**C**(O)O), 160.7, 157.5, 151.9 (Ar–**C**$_{ortho}$–O–CH$_2$), 146.1 (Ar–**C**$_{ipso'}$), 128.5, 126.5 (Ar–C$_{ortho'}$, Ar–C$_{meta}$), 119.1 (Ar–**C**$_{ipso}$), 118.6, 115.9, 113.4 (Ar–**C**$_{para}$, Ar–**C**$_{meta'}$, Ar–**C**$_{meta}$), 80.9 (–O–**C**H$_2$–), 70.3 (–O–**C**H$_2$–), 68.2 (–O–**C**H$_2$–), 66.9 (–O–**C**H$_2$–), 25.3 (H$_3$**C**–C(O)O), 23.6 (H$_3$**C**–C(O)O).

Anal. Calcd for $C_{30}H_{30}N_2NaO_{10}Zn$: C, 54.55; H, 4.58; N, 4.24. Found: C, 54.35; H, 4.44; N, 4.14.

HRMS (ESI/FTMS) m/z: [**10** - OAc]$^+$ Calcd for $C_{28}H_{27}CoN_2NaO_8$ 601.0992; Found 601.0980.

Synthesis of 11: A methanol (350 mL) solution of **LH$_2$** (400 mg, 1.03 mmol) and sodium acetate (84.1 mg, 1.03 mmol) was refluxed and a methanol (250 mL) solution of 2,2-dimethylpropane-1,3-diamine (105 mg, 1.03 mmol) was added, dropwise, over the course of 3 h. The solution was left to cool to room temperature and the solvent was removed under reduced pressure. The resultant solid was washed with deionized water (3 x 100 mL), then dissolved in MeOH (200 mL). 20 equiv. of $NaBH_4$ (779 mg, 20.6 mmol) was added in one portion and left to stir for 2h. Water was added to quench the excess sodium borohydride and the solvent was subsequently removed under reduced pressure. The resulting solid was washed with distilled water (3 x 100 mL). The solid (300 mg, 0.65 mmol) is then dissolved in MeOH (200 mL) before the $Zn(OAc)_2{\cdot}2(H_2O)$ (143 mg, 0.65 mmol) and sodium acetate (53.2 mg, 0.65 mmol) was added and left to stir for 1 h. The solvent was removed, under reduced pressure, to obtain a yellow glassy solid as the crude. This was triturated with ethanol (50 mL) and dichloromethane (50 mL) and dried under vacuum at 40 °C to give a pure product (256 mg, 0.42 mmol, 65%).

^1H NMR (500 MHz, $C_2D_2Cl_4$, 298 K). δ(ppm): 6.79 (2H, d, Ar–**H**$_{meta}$, $^3J_{H\text{-}H}$ = 7.9 Hz), 6.67 (2H, d, Ar–**H**$_{meta}$, $^3J_{H\text{-}H}$ = 7.4 Hz), 6.50 (2H, t, Ar–**H**$_{para}$, $^3J_{H\text{-}H}$ = 7.7 Hz), 4.22-3.58 (16H, m, , –O–C**H**$_2$–, –NH–C**H**$_2$–Ar), 3.16 (CH$_2$–N**H**–CH$_2$), 2.75 (2H, t, –NH–C**H**$_2$–C(CH$_3$)$_2$–, $^3J_{H\text{-}H}$ = 12.4 Hz), 2.41 (2H, d, –NH–C**H**$_2$–C(CH$_3$)$_2$–, $^3J_{H\text{-}H}$ = 11.4 Hz), 1.83 (6H?, s, **H**$_3$C–C(O)O), 1.23 (3H, s, C–C**H$_3$**), 0.90 (3H, s, C–C**H$_3$**).

^{13}C NMR (125 MHz, $C_2D_2Cl_4$, 298 K). δ(ppm): 177.1 (–**C**(O)O), 154.7 (Ar–**C**$_{ortho}$–O–CH$_2$), 149.6 (Ar–**C**$_{ortho}$–CH$_2$), 124.9 (Ar–**C**$_{ipso}$), 123.7 (Ar–**C**$_{meta}$), 114.33 (Ar–**C**$_{meta}$), 113.6 (Ar–**C**$_{para}$), 69.40 (–O–**C**H$_2$–), 69.12 (–O–**C**H$_2$–), 67.93 (–O–**C**H$_2$–), 61.82 ((CH$_3$)$_2$C–**C**H$_2$–NH$_2$), 54.19 (NH$_2$–**C**H$_2$–Ar), 34.12 (**C**(CH$_3$)$_2$), 31.17 (C–**C**H$_3$), 22.52 (H$_3$**C**–C(O)O, C–**C**H$_3$).

Anal calcd (found) for $C_{27}H_{37}N_2NaO_8Zn$: C, 53.52 (53.67); H, 6.15 (6.06); N 4.62 (4.68).

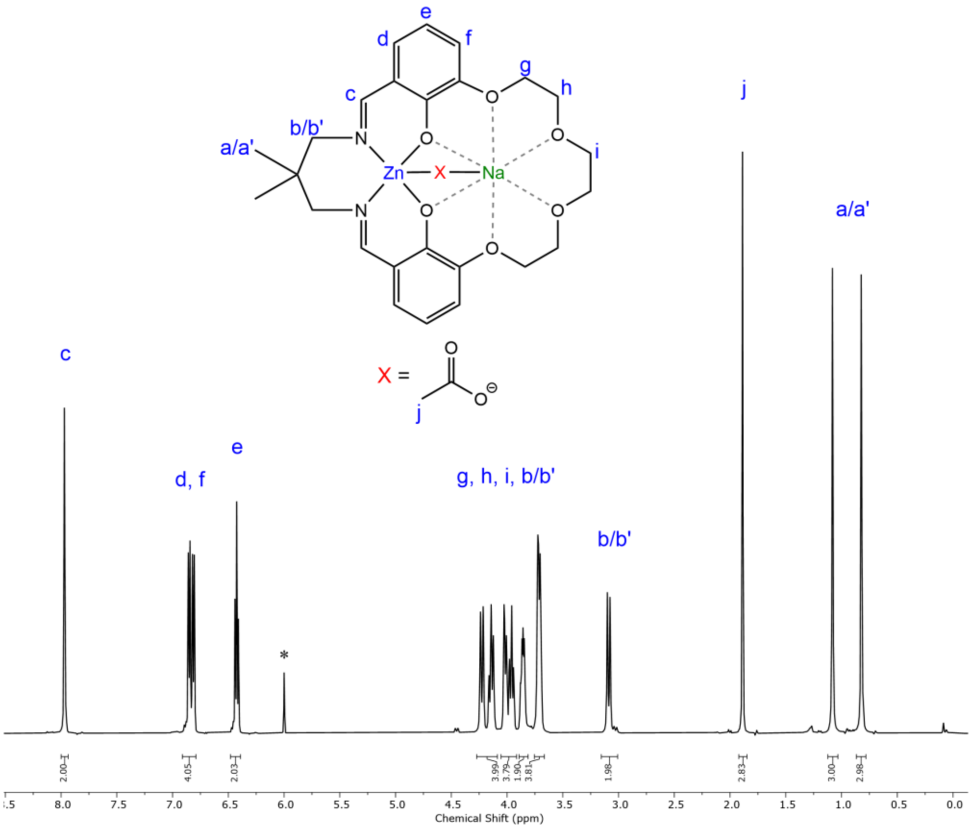

Figure S1. ^{1}H NMR spectrum of **1** (d_2-TCE, 298K), * indicates residual protic NMR solvent.

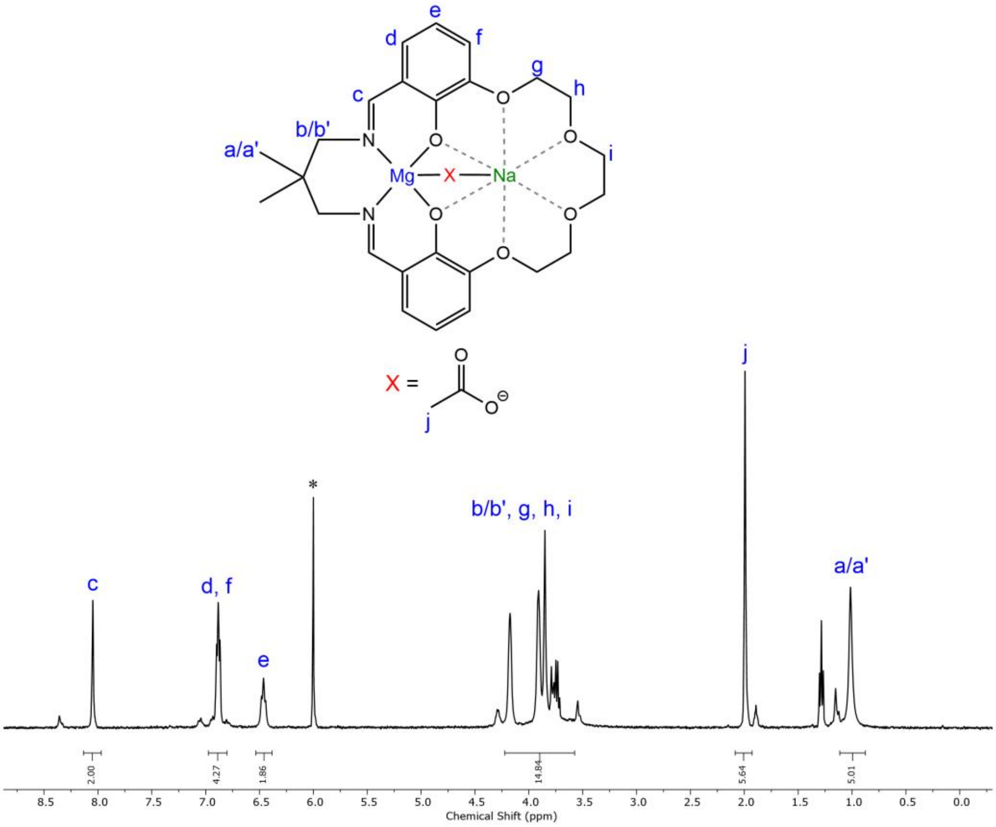

Figure S2. ^{1}H NMR spectrum of **2** (d_2-TCE, 398K), * indicates residual protic NMR solvent.

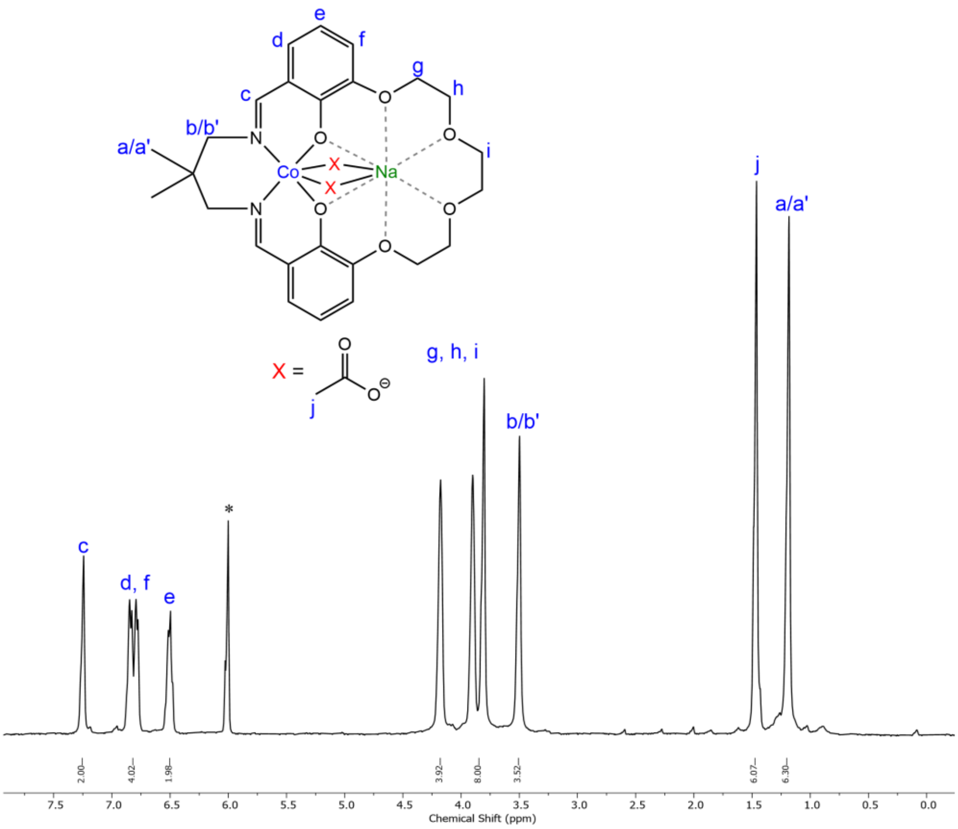

Figure S3. ^{1}H NMR spectrum of **3** (d_2-TCE, 298K), * indicates residual protic NMR solvent.

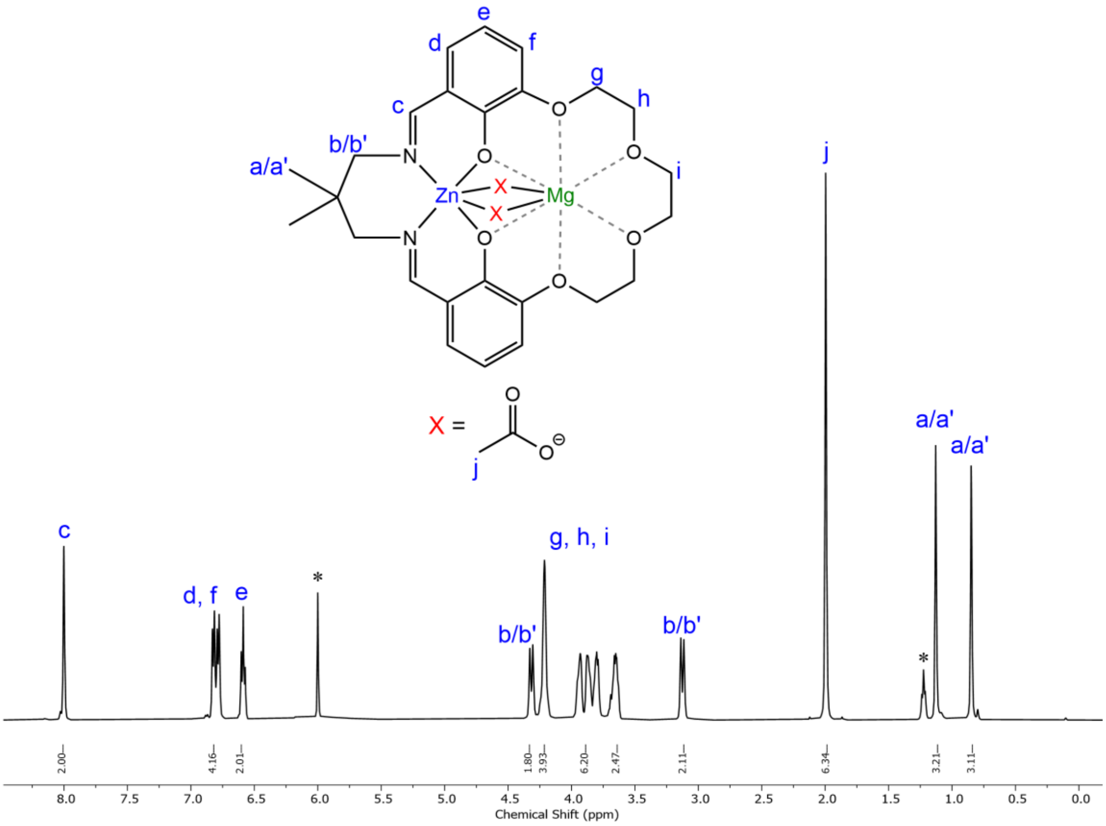

Figure S4. ^{1}H NMR spectrum of **4** (d_2-TCE, 328K), * indicates residual protic NMR solvent and pentane.

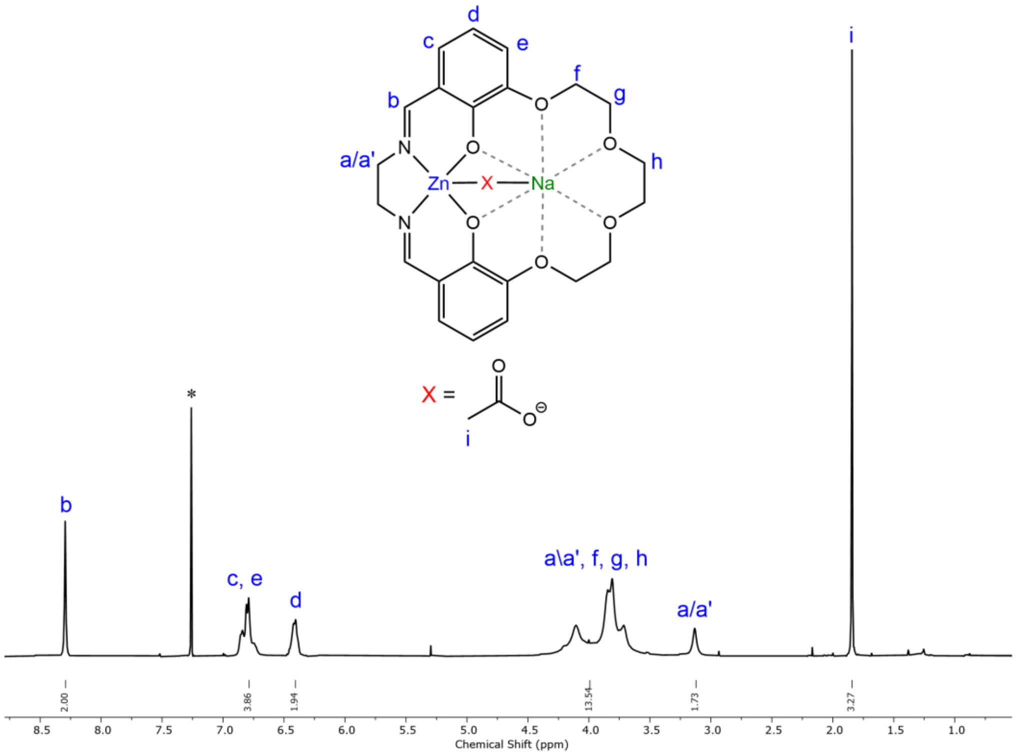

Figure S5. ^{1}H NMR spectrum of **5** ($CDCl_3$, 298K), * indicates residual protic solvent.

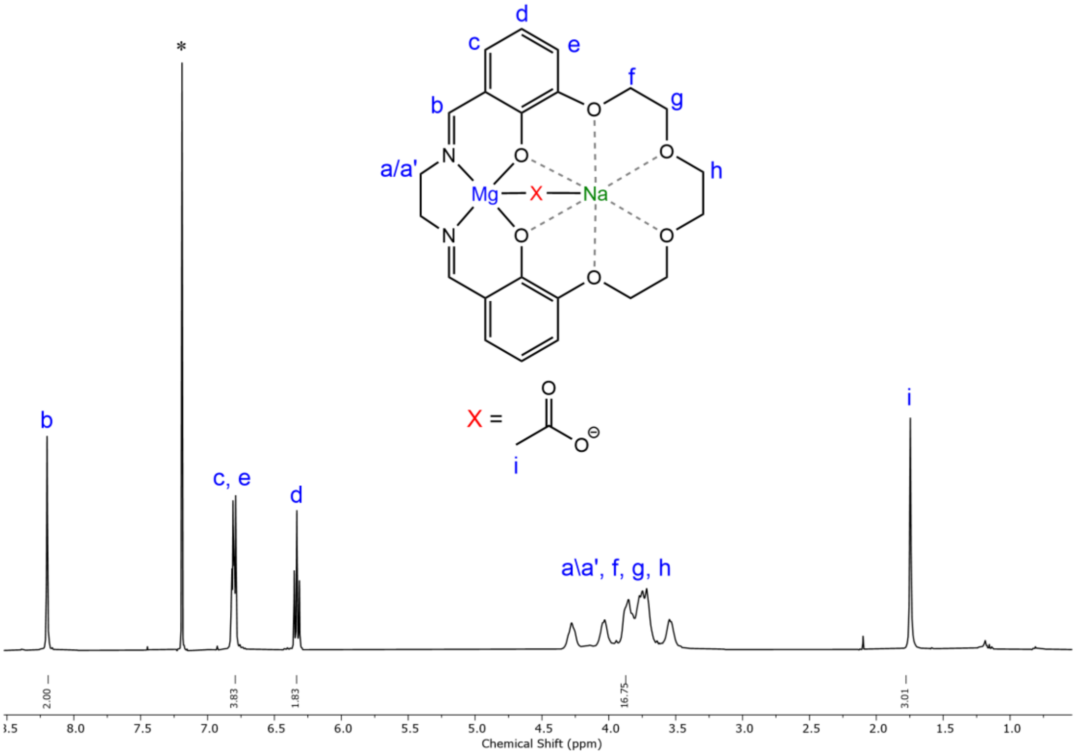

Figure S6. ^{1}H NMR spectrum of **6** ($CDCl_3$, 298K), * indicates residual protic solvent.

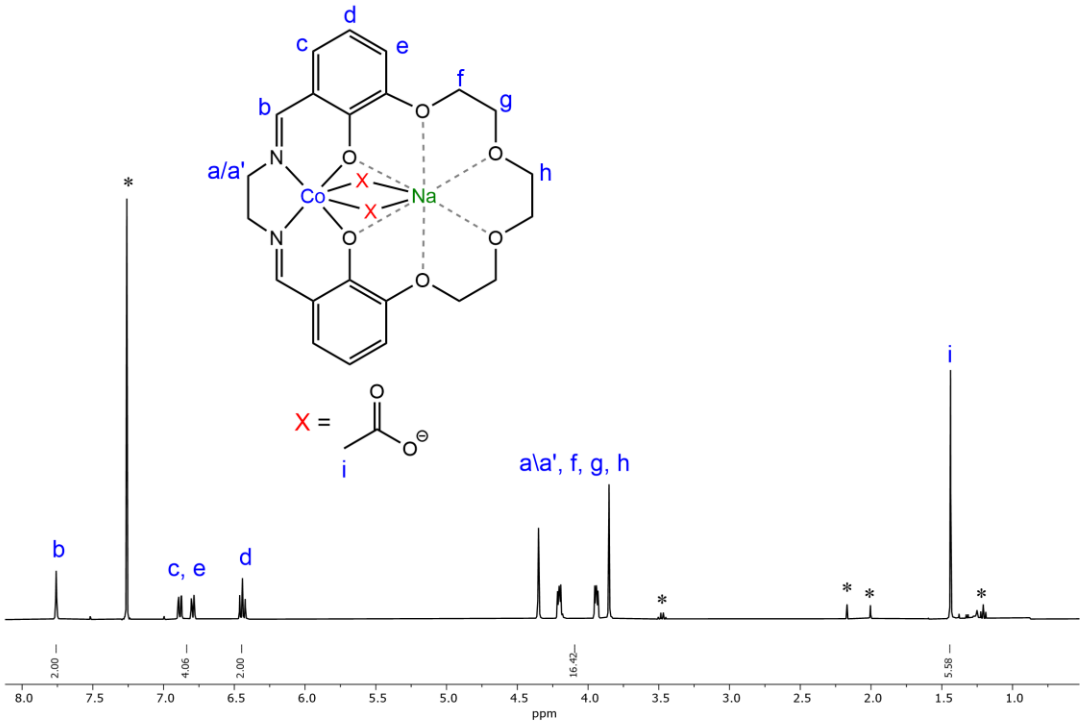

Figure S7. ^{1}H NMR spectrum of **7** ($CDCl_3$, 298K), * indicates residual protic NMR solvent as well as residual ethanol, acetone and acetonitrile not removed under vacuum.

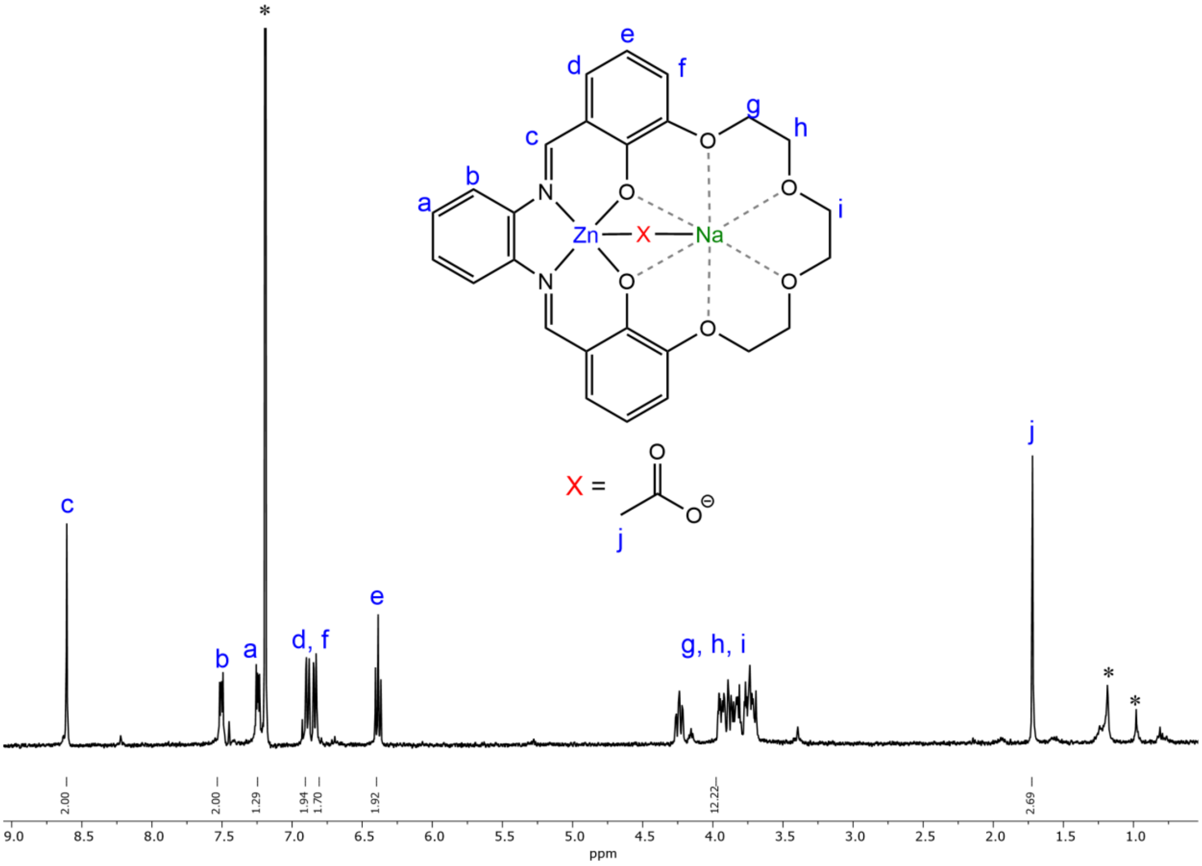

Figure S8. ^{1}H NMR spectrum of **8** ($CDCl_3$, 298K), * indicates residual protic NMR solvent as well as pentane found in the NMR solvent.

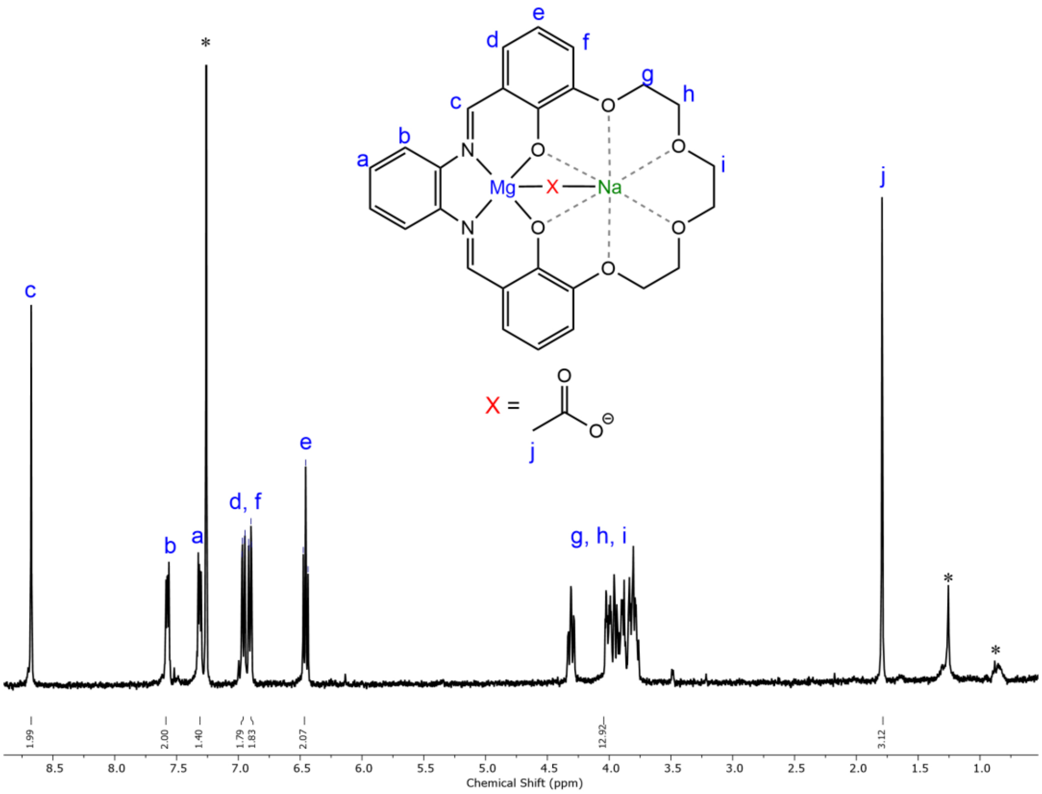

Figure S9. ^{1}H NMR spectrum of **9** ($CDCl_3$, 298K), * indicates residual protic NMR solvent as well as pentane found in the NMR solvent.

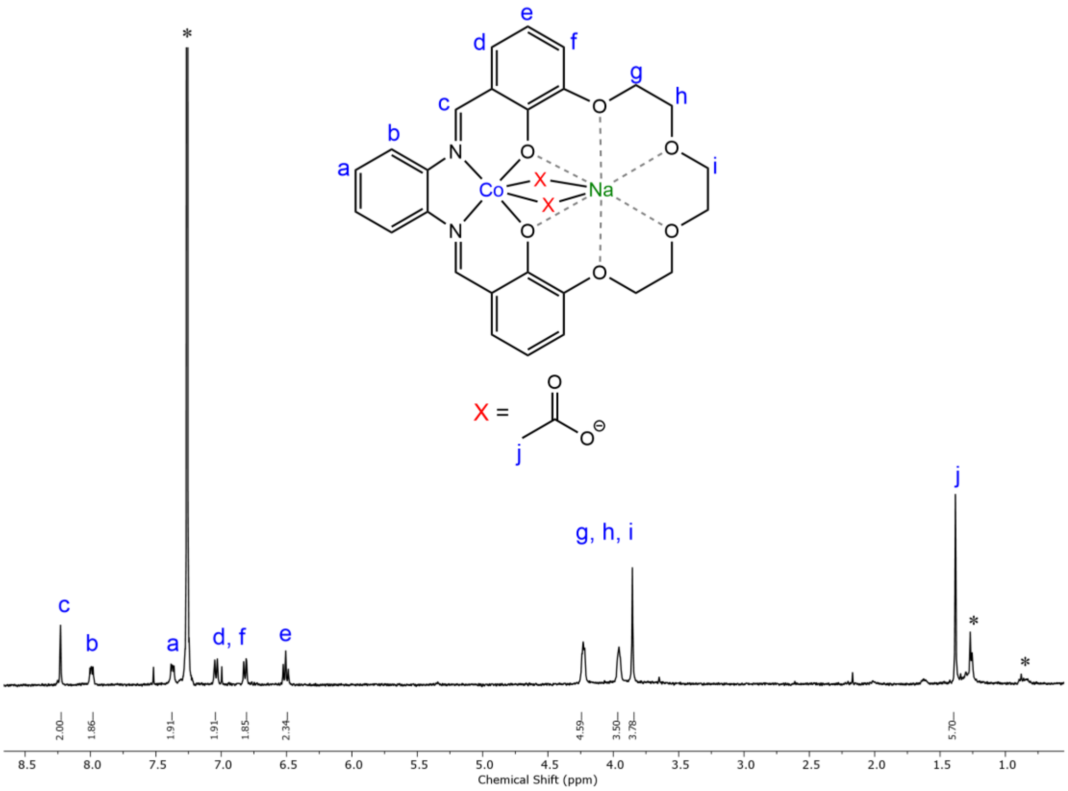

Figure S10. ^{1}H NMR spectrum of **10** ($CDCl_3$, 298K), * indicates residual protic NMR solvent as well as pentane found in the NMR solvent.

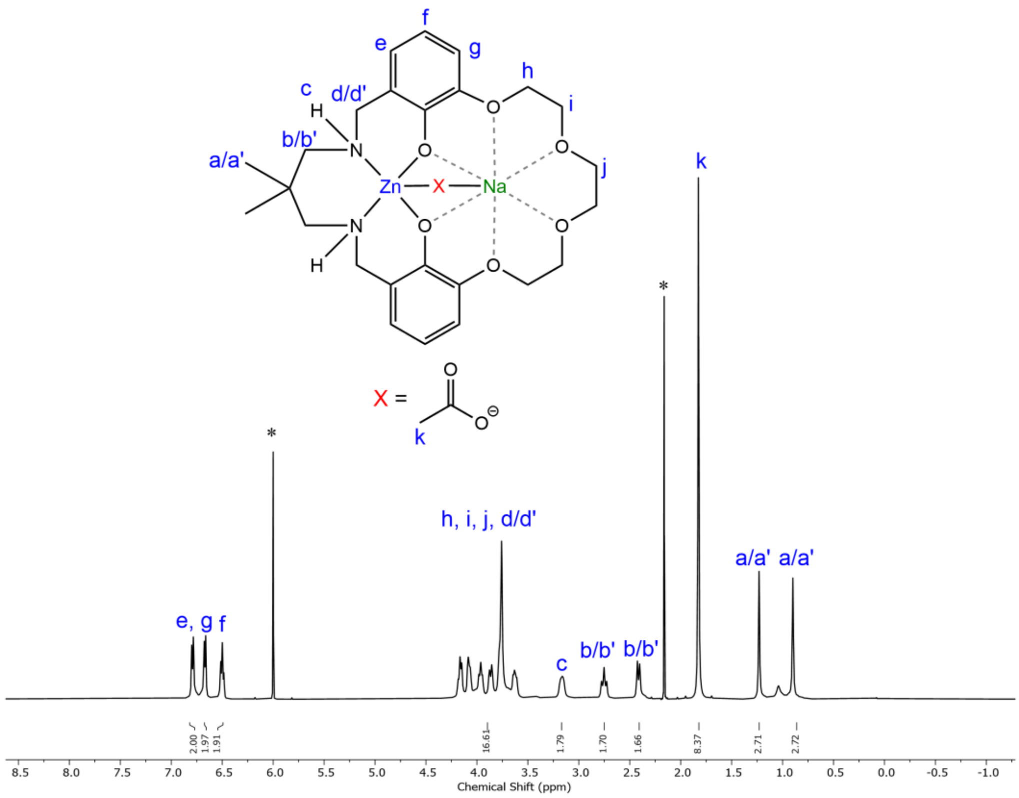

Figure S11. ^{1}H NMR spectrum of **11** (d_2-TCE, 298K), * indicates residual protic NMR solvent as well as acetone found in the NMR tube.

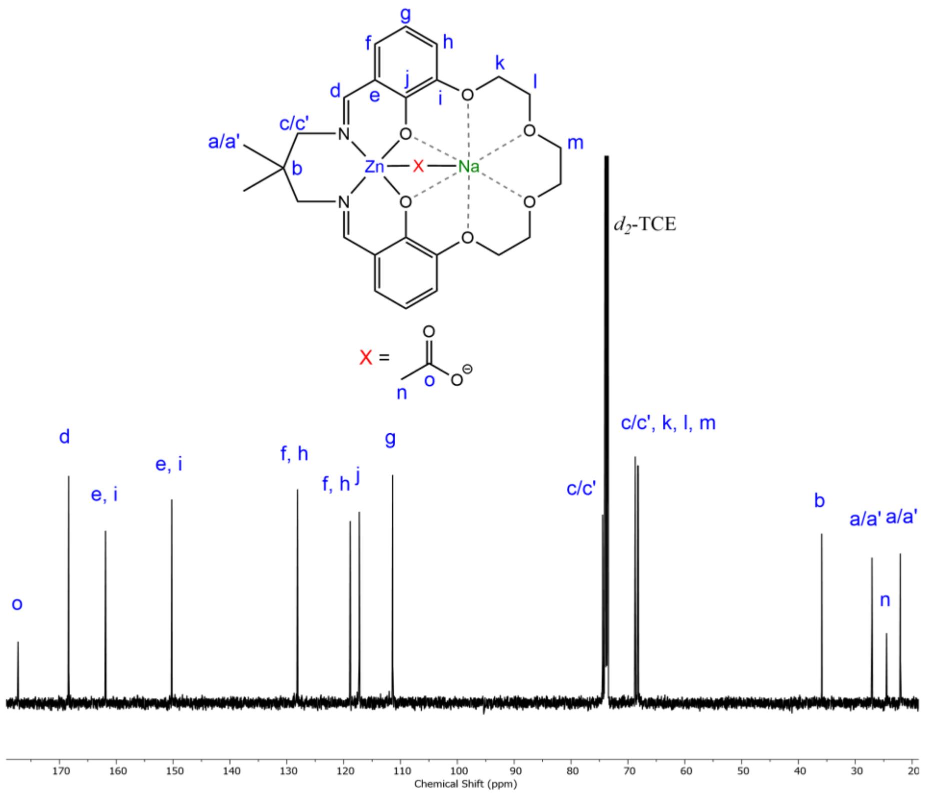

Figure S12. ^{13}C NMR spectrum of **1** (d_2-TCE, 298K).

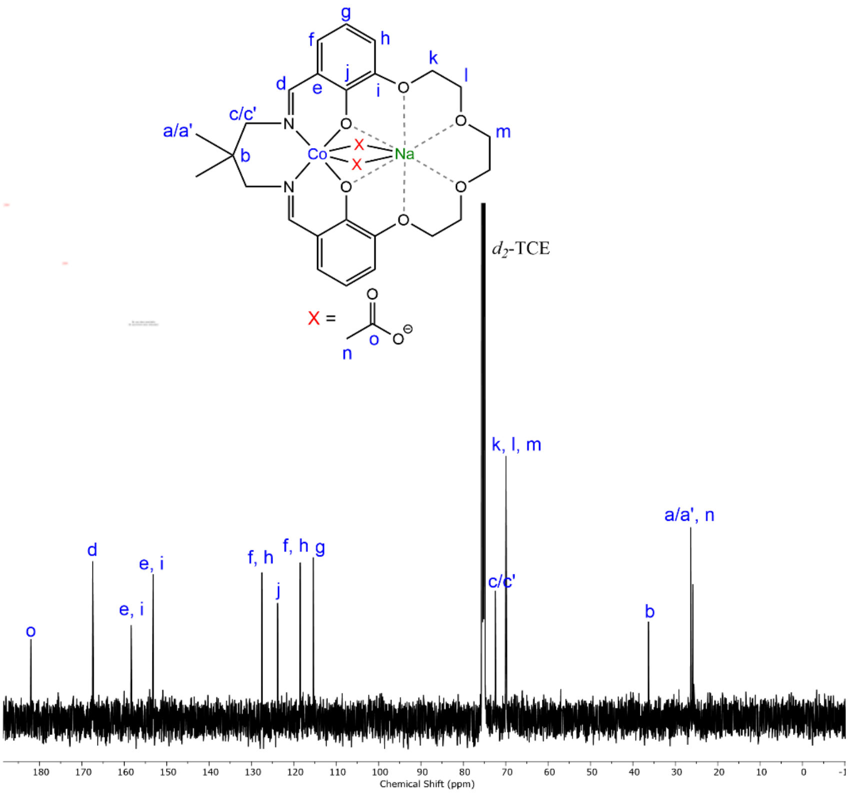

Figure S13. ^{13}C NMR spectrum of **3** (d_2-TCE, 298K).

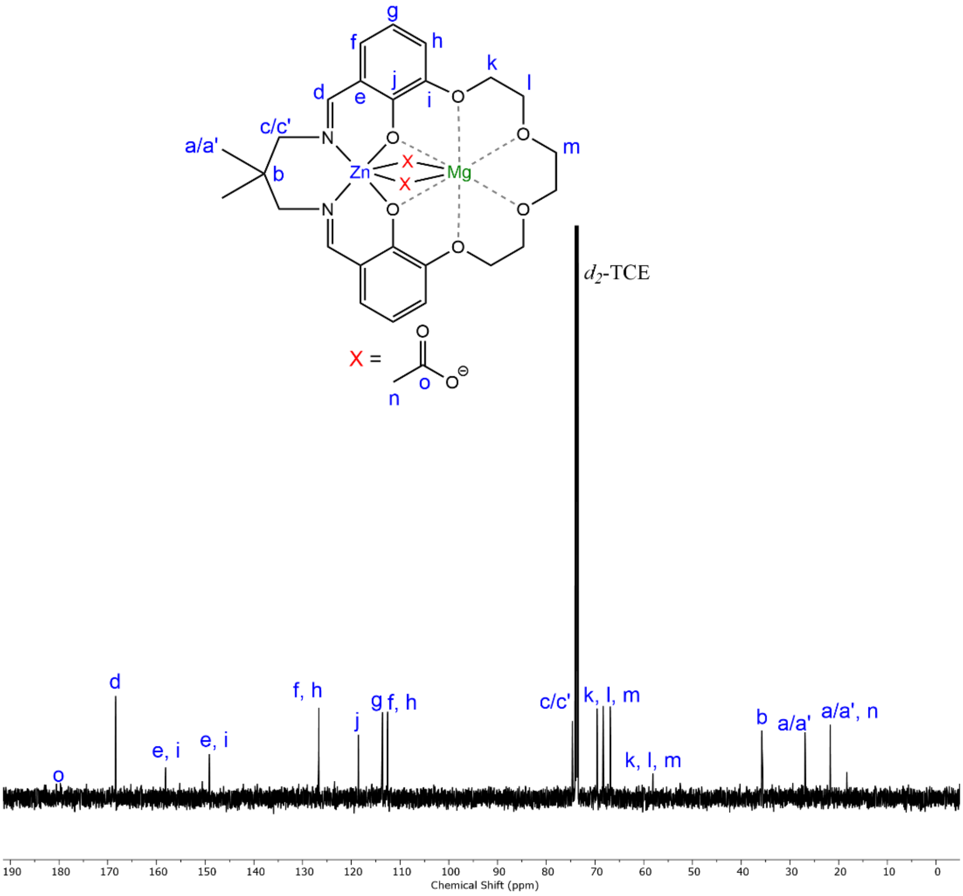

Figure S14. ^{13}C NMR spectrum of **4** (d_2-TCE, 328K).

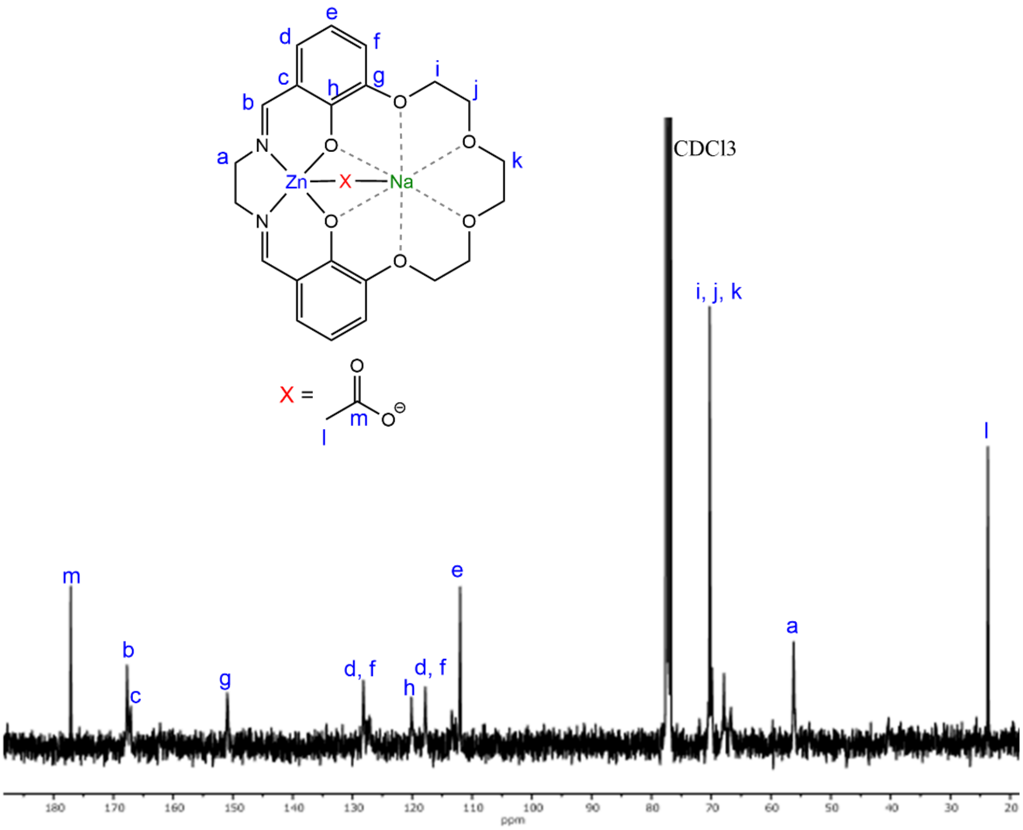

Figure S15. ^{13}C NMR of **5** ($CDCl_3$, 298K).

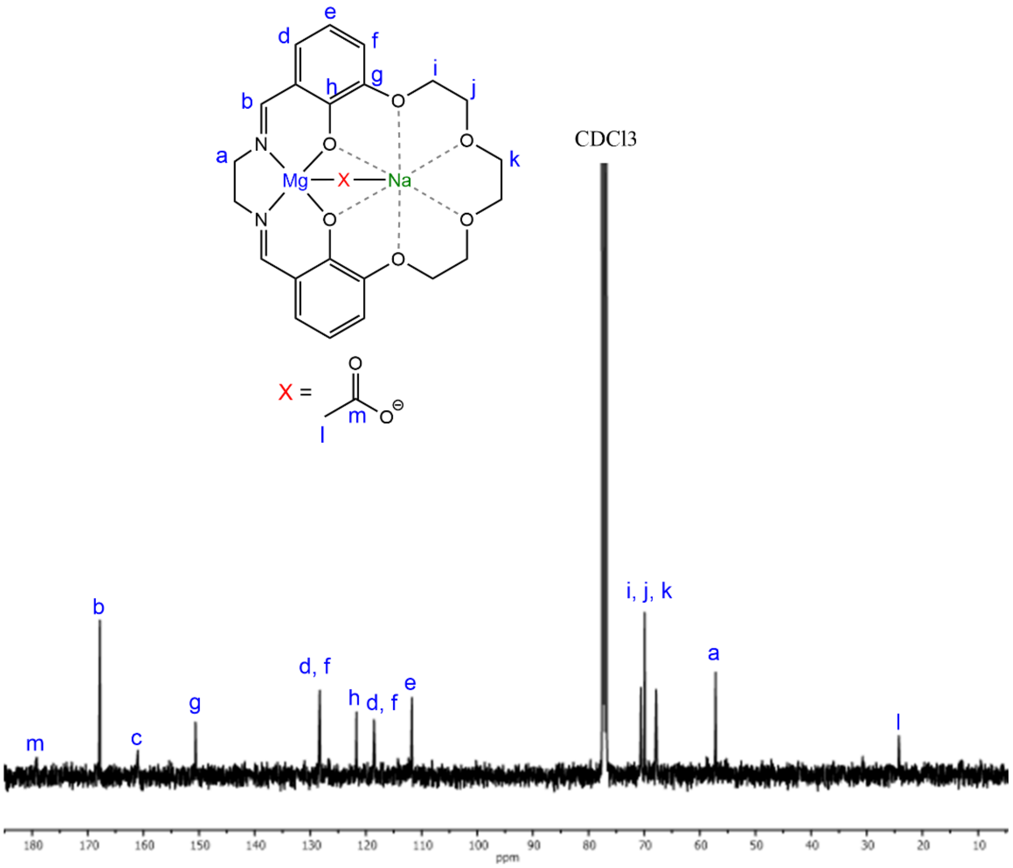

Figure S16. ^{13}C NMR of **6** ($CDCl_3$, 298K).

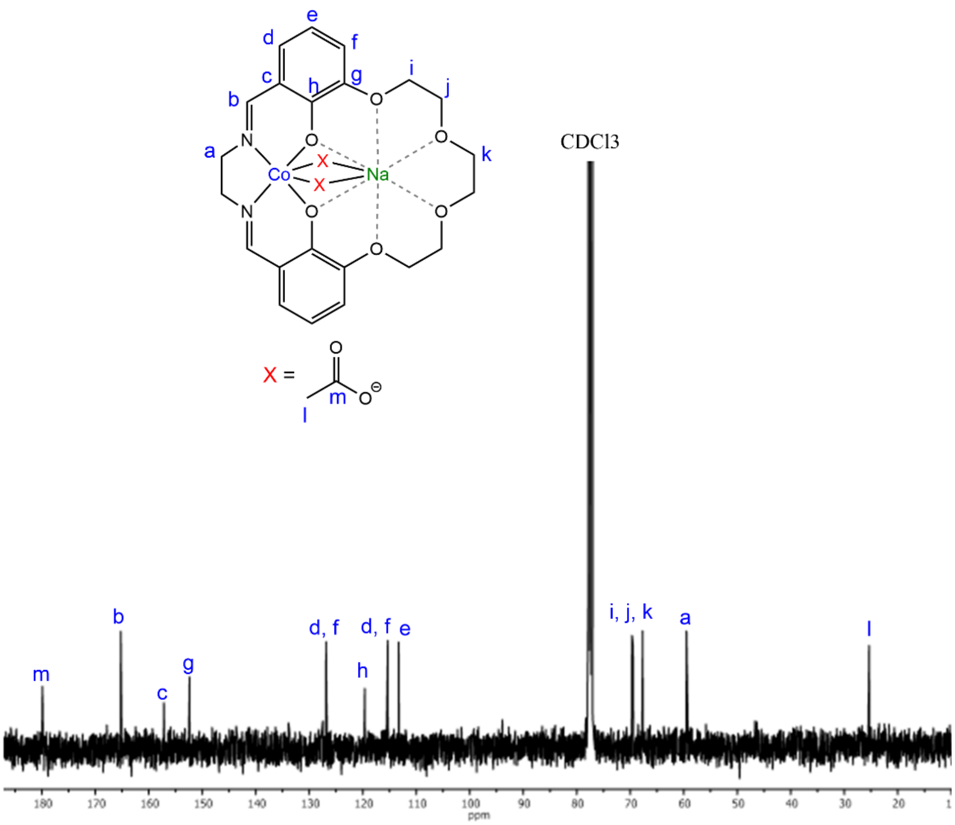

Figure S17. ^{13}C NMR of **7** ($CDCl_3$, 298K).

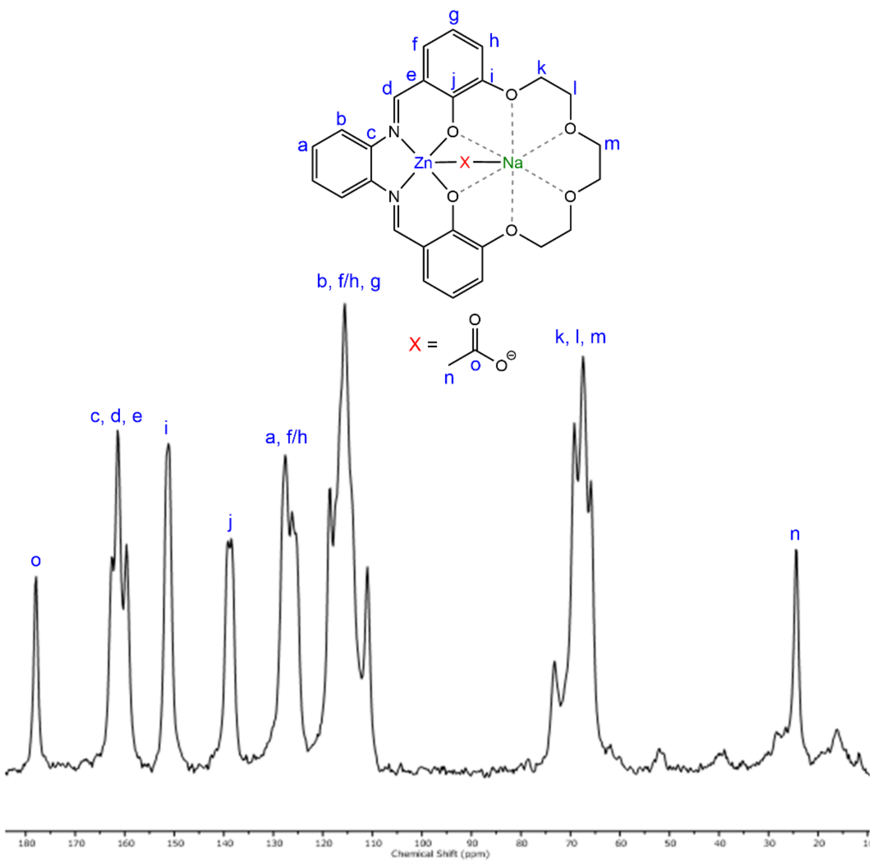

Figure S18. ^{13}C NMR of **8** ($CDCl_3$, 298K) (solid state).

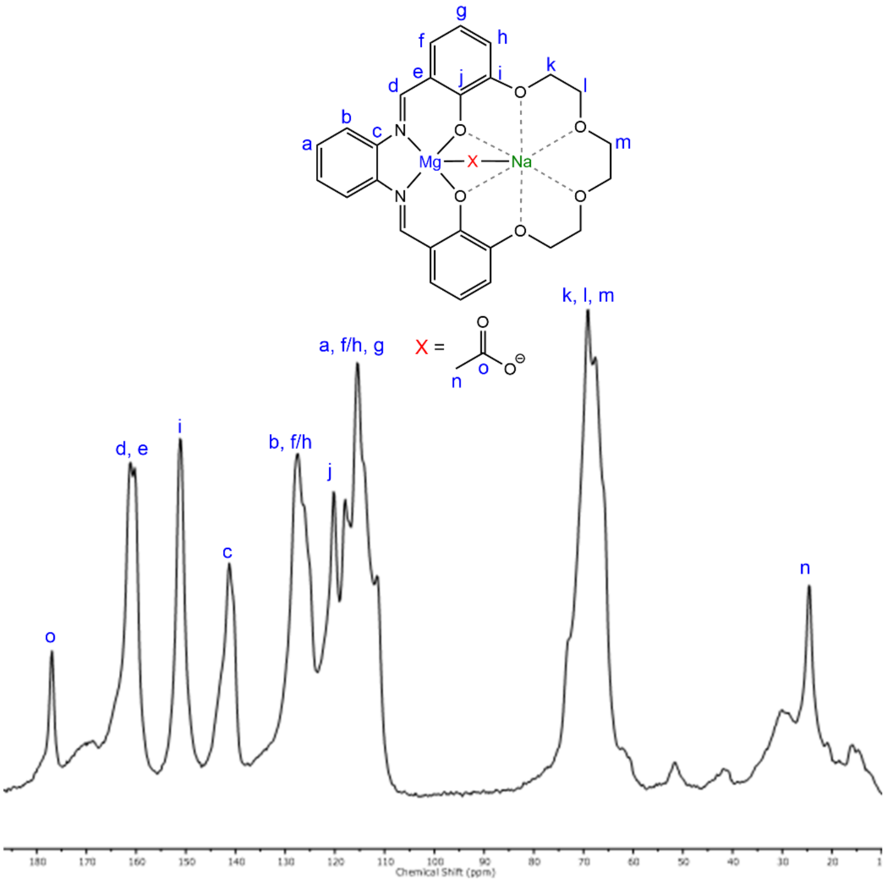

Figure S19. ^{13}C NMR of **9** ($CDCl_3$, 298K) (solid state).

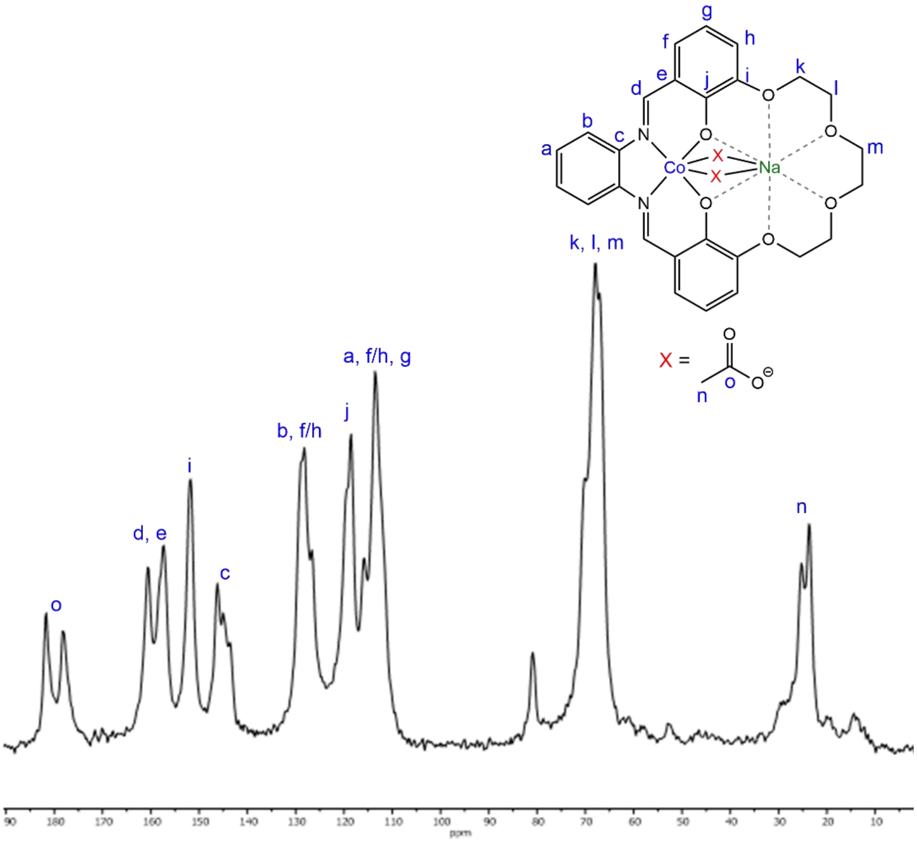

Figure S20. ^{13}C NMR of **10** ($CDCl_3$, 298K) (solid state).

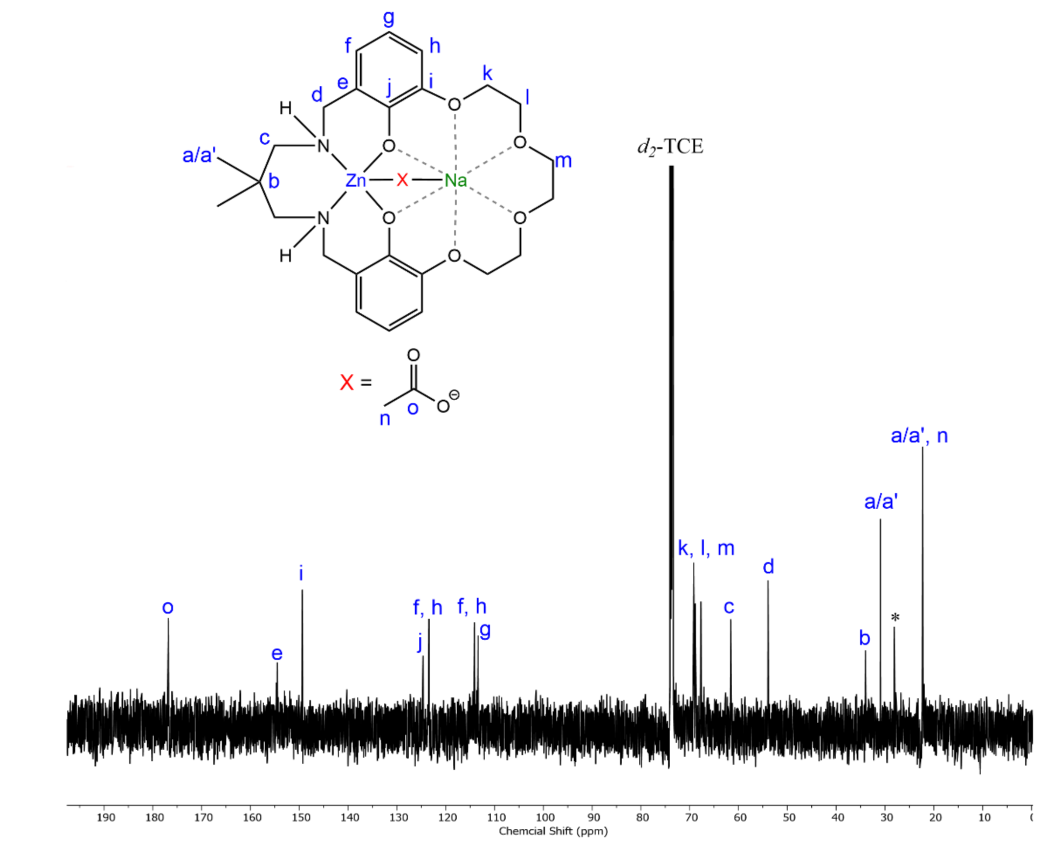

Figure S21. ^{13}C NMR of **11** (d_2-TCE, 298K), * residual acetone in NMR solvent.

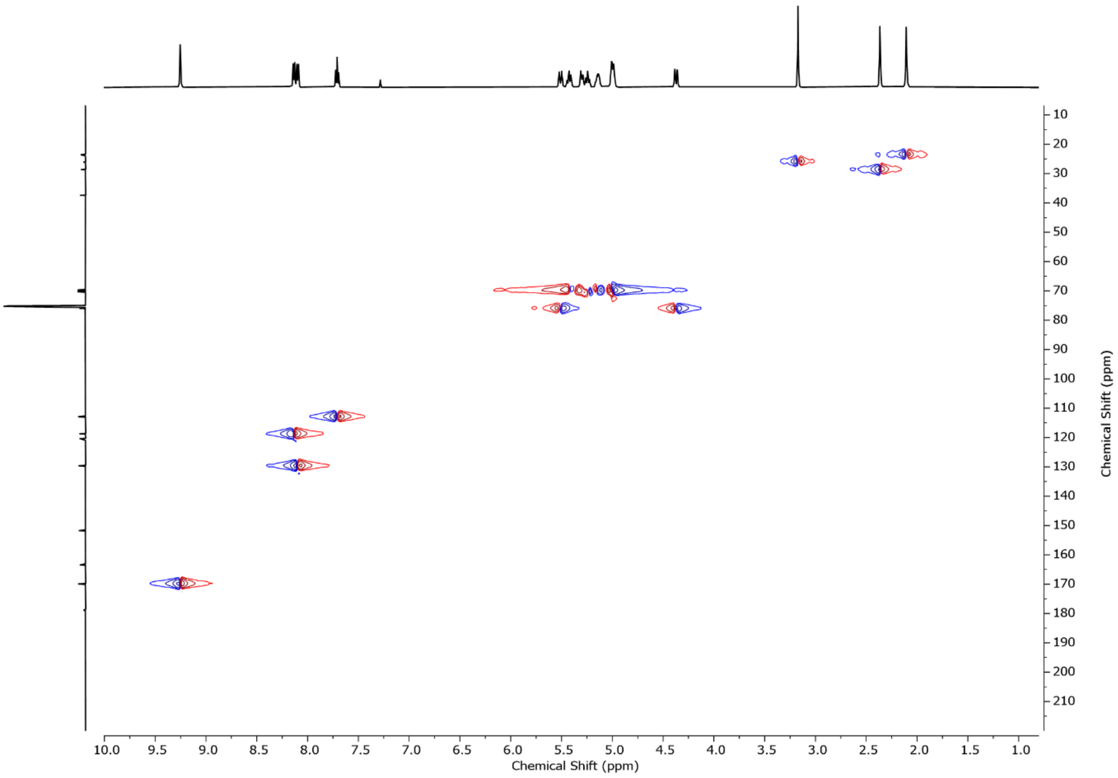

Figure S22. 2D HSQC NMR of **1** (d_2-TCE, 298K).

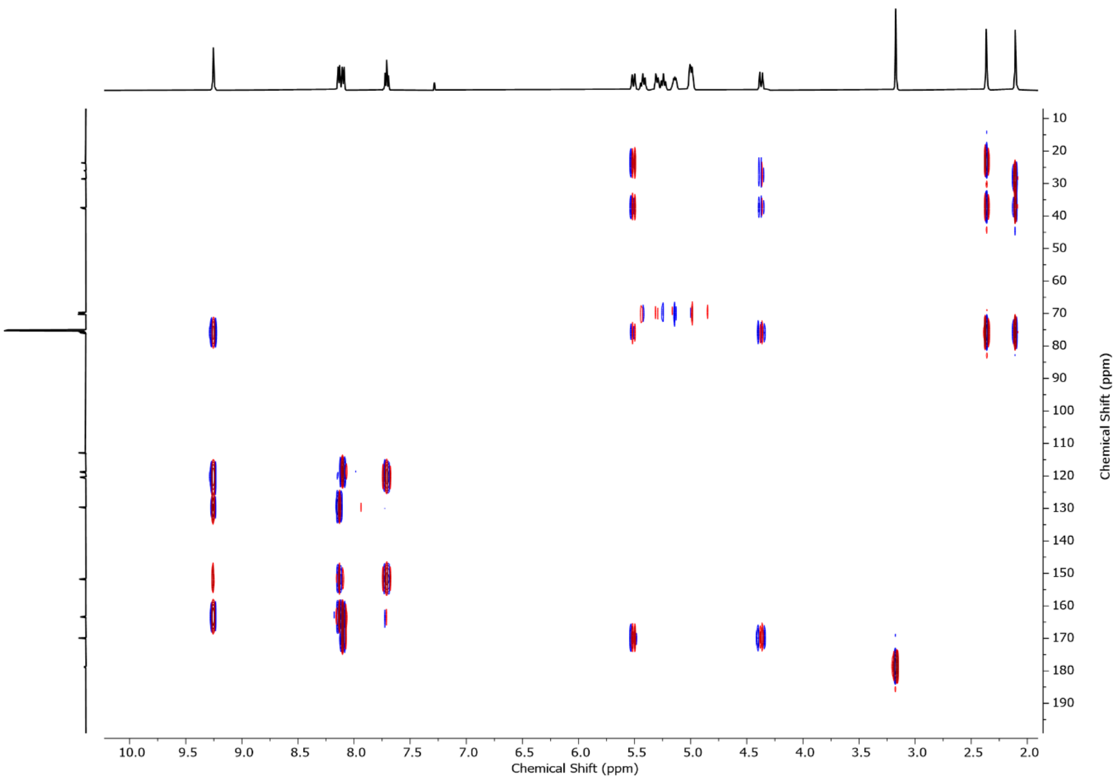

Figure S23. 2D HMBC NMR of **1** (d_2-TCE, 298K).

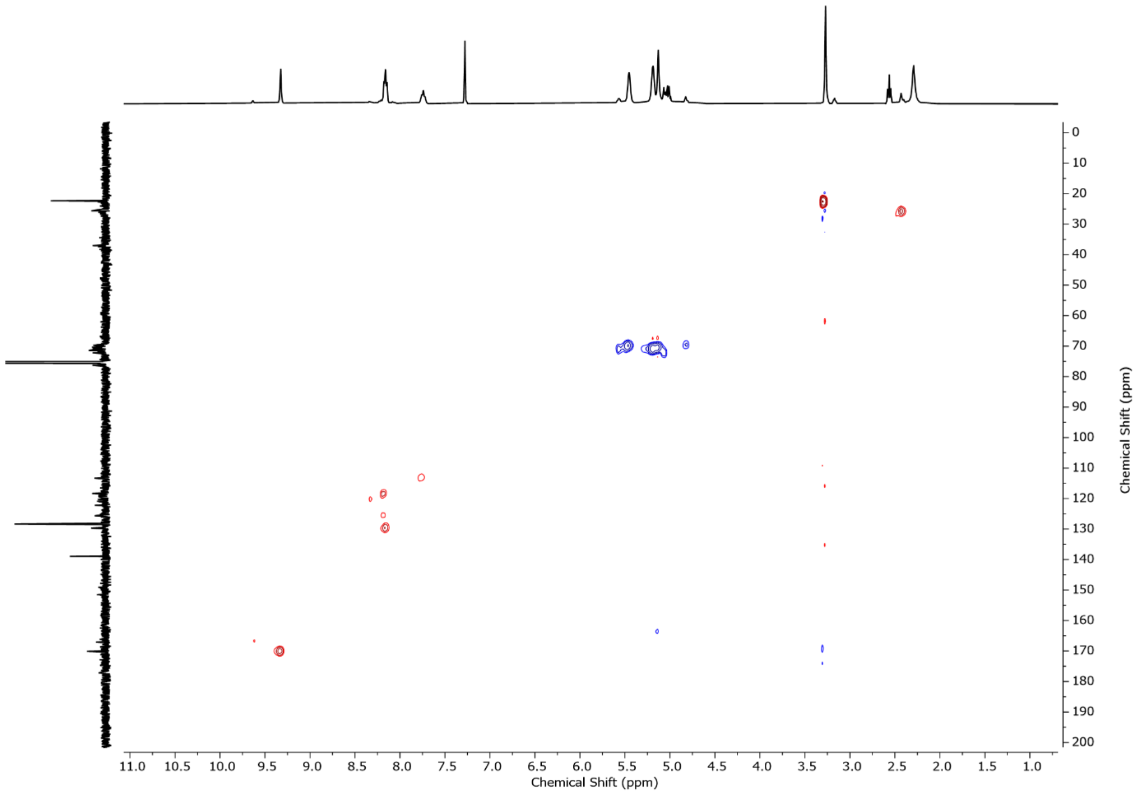

Figure S24. 2D HSQC NMR of **2** (d_2-TCE, 398K).

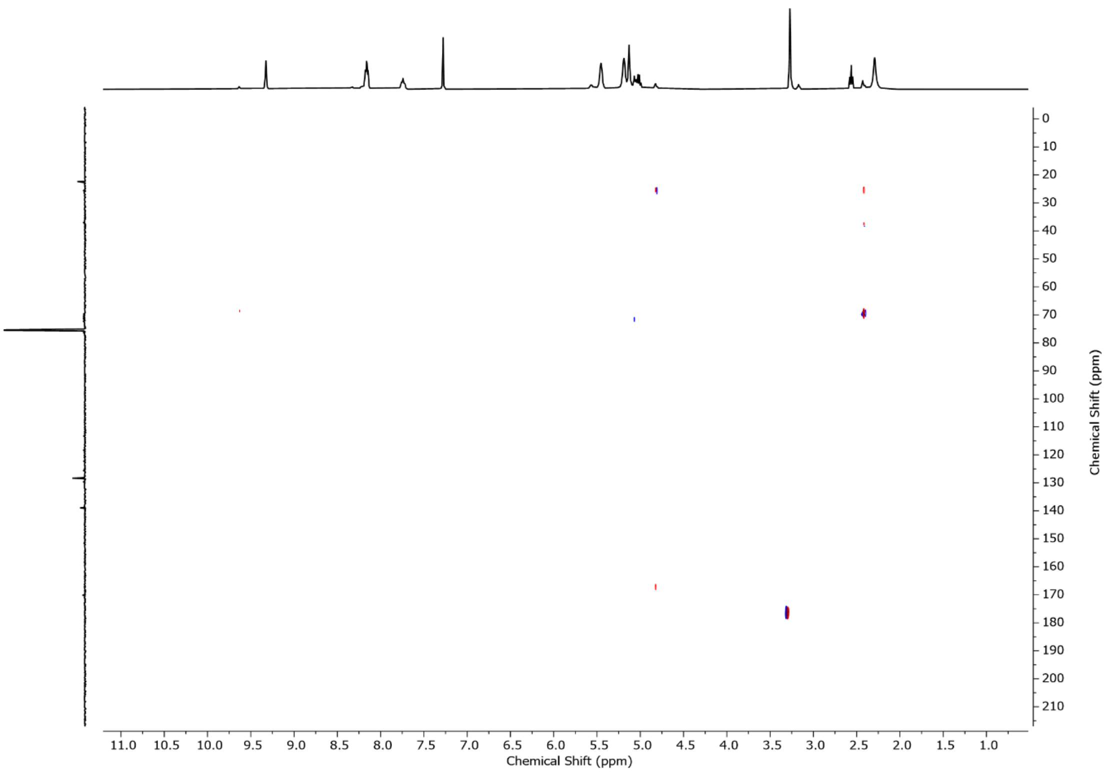

Figure S25. 2D HMBC NMR of **2** (*d*$_2$-TCE, 398K).

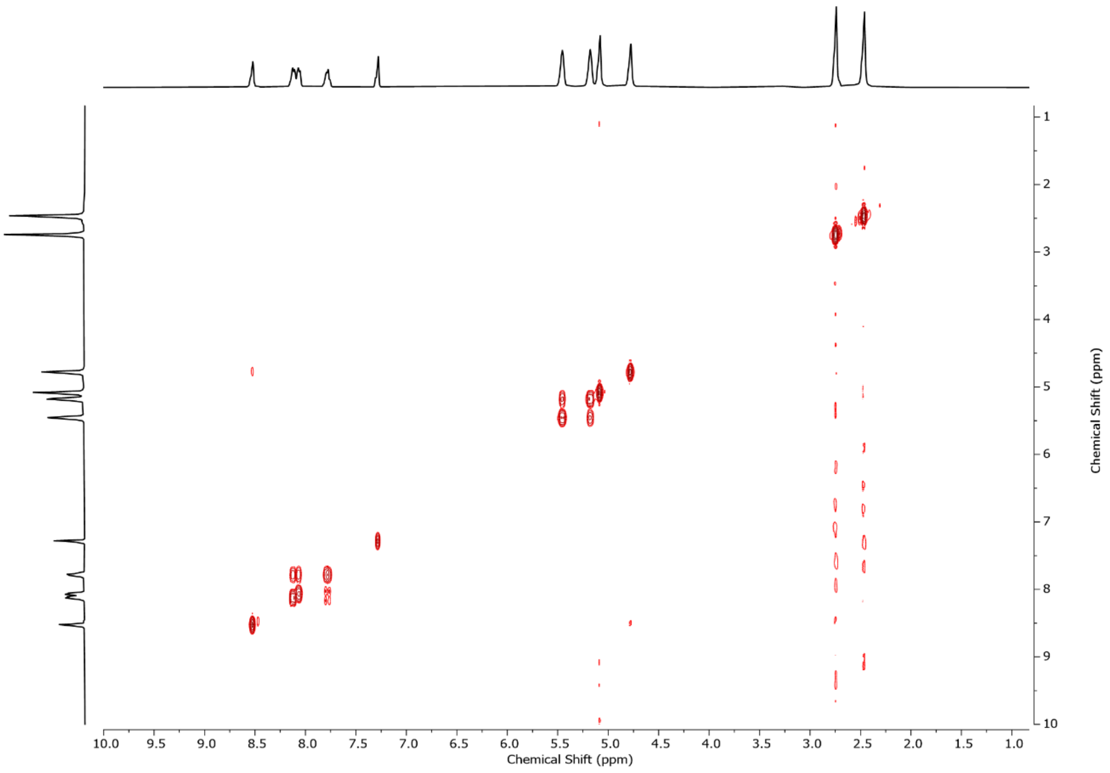

Figure S26. 2D COSY NMR of **3** (*d*$_2$-TCE, 298K).

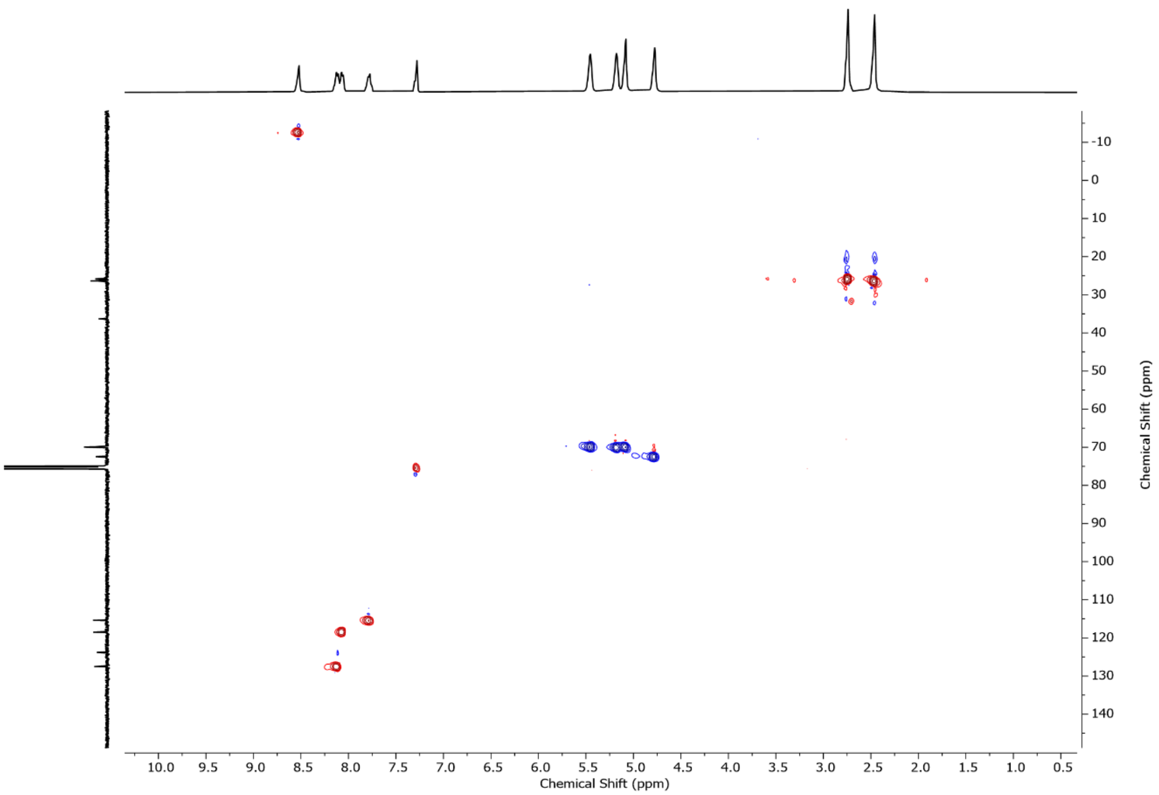

Figure S27. 2D HSQC NMR of **3** (d_2-TCE, 298K).

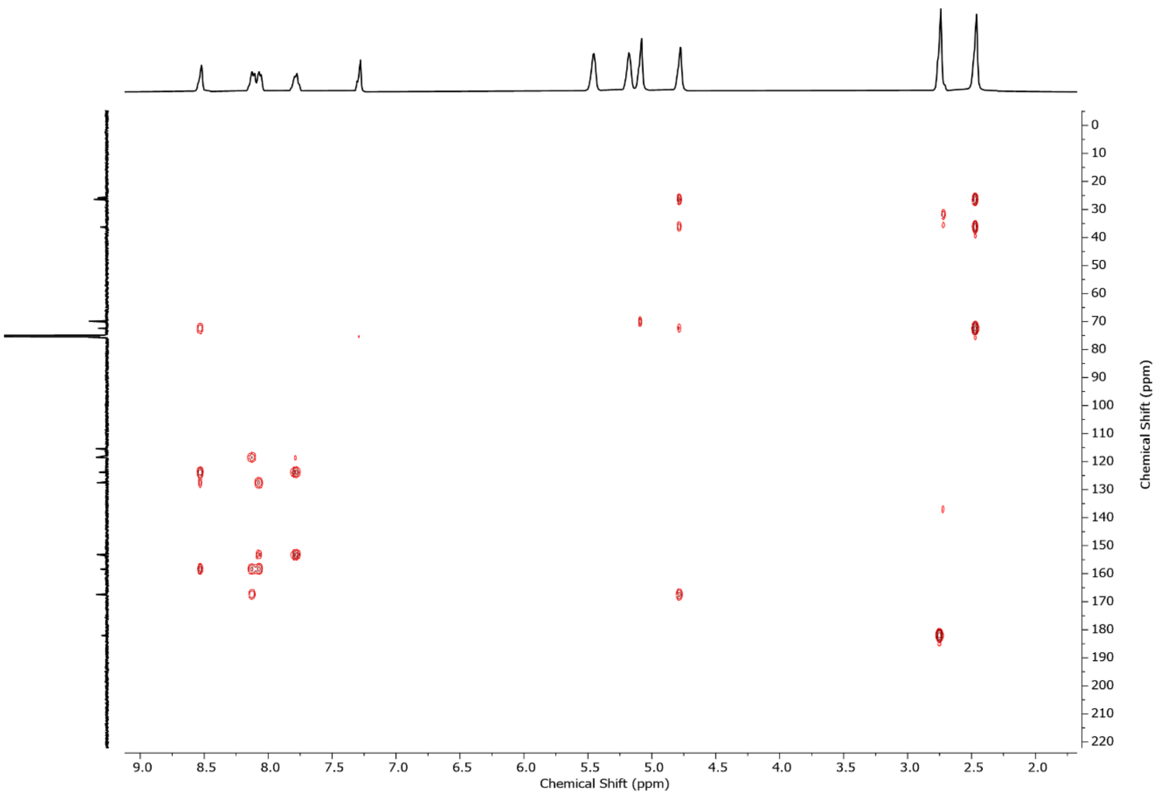

Figure S28. 2D HMBC NMR of **3** (d_2-TCE, 298K).

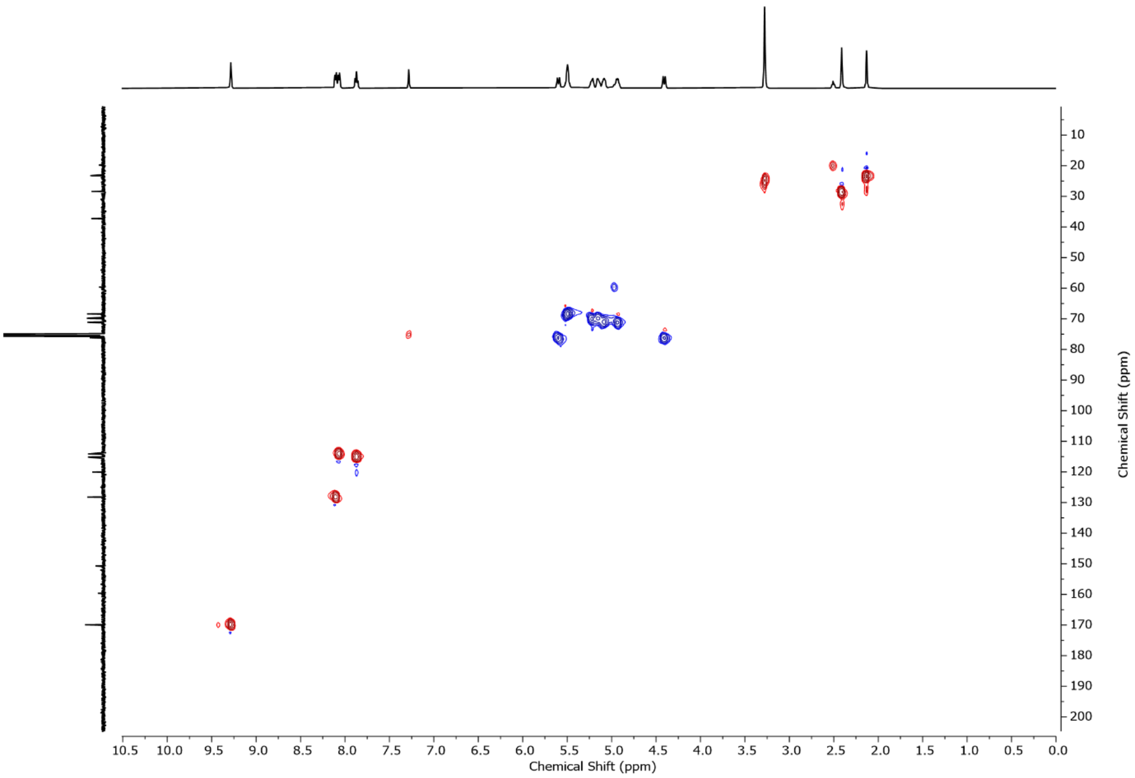

Figure S29. 2D HSQC NMR of **4** (*d_2*-TCE, 328K).

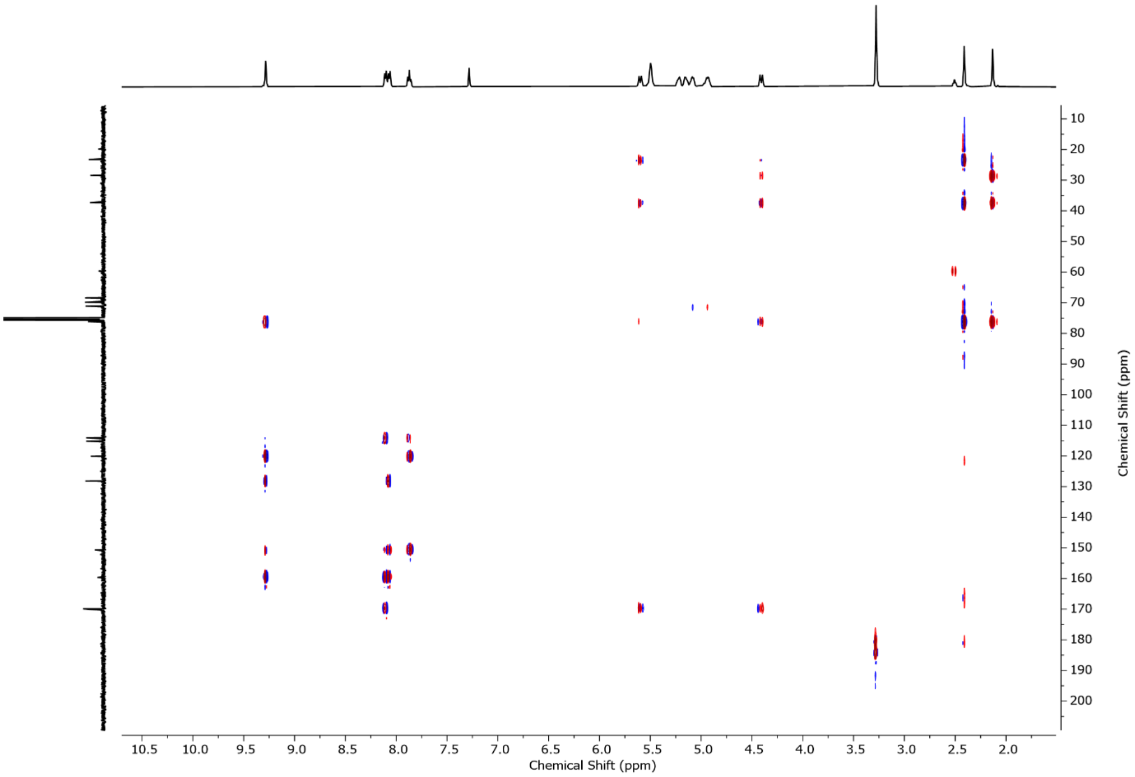

Figure S30. 2D HMBC NMR of **4** (*d_2*-TCE, 328K).

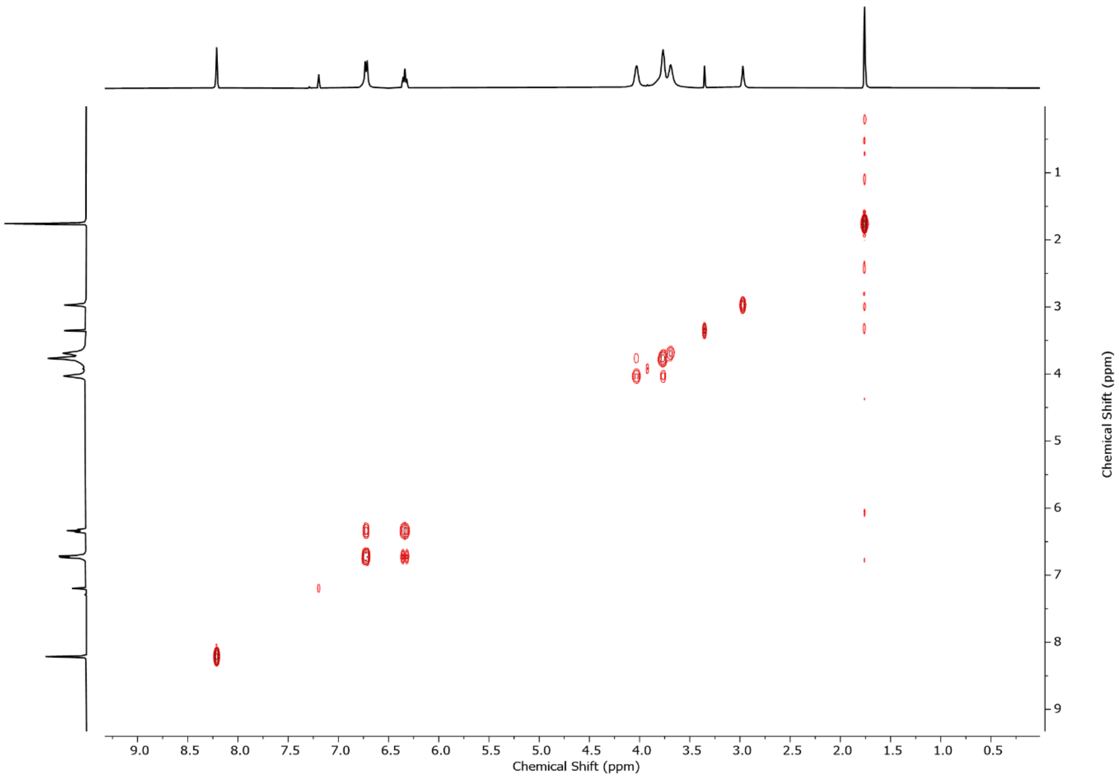

Figure S31. 2D COSY NMR of **5** ($CDCl_3$, 298K).

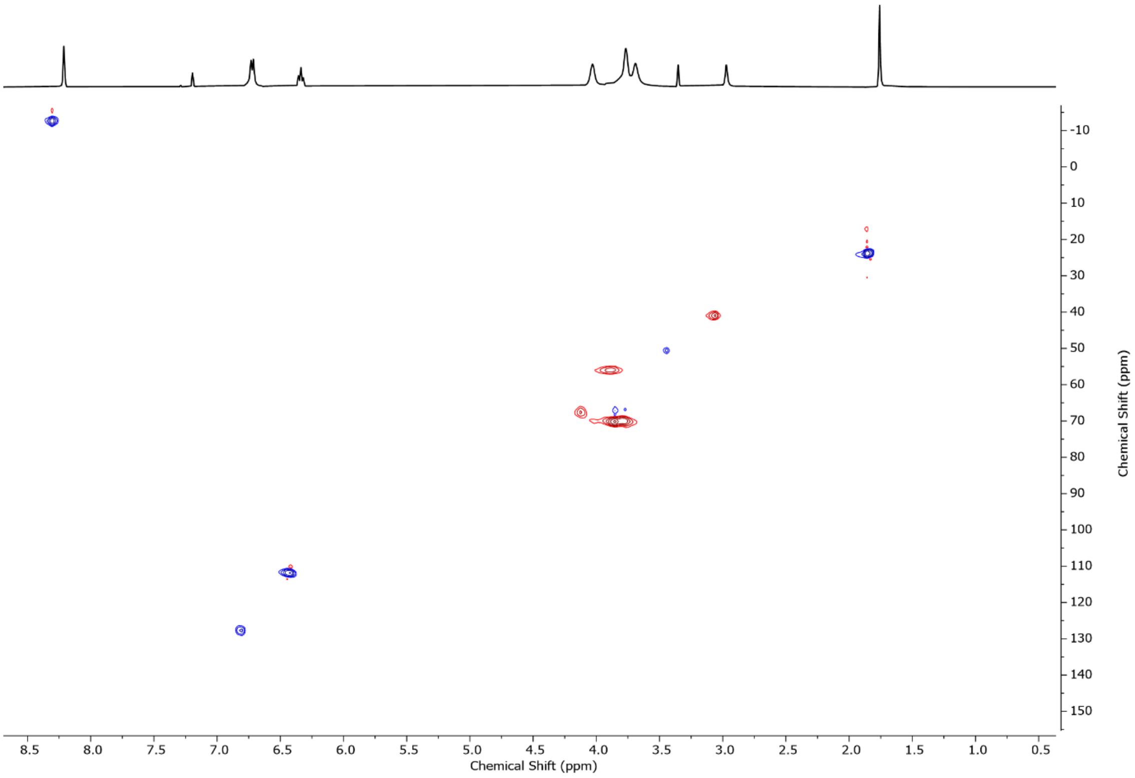

Figure S32. HSQC NMR of **5** ($CDCl_3$, 298K).

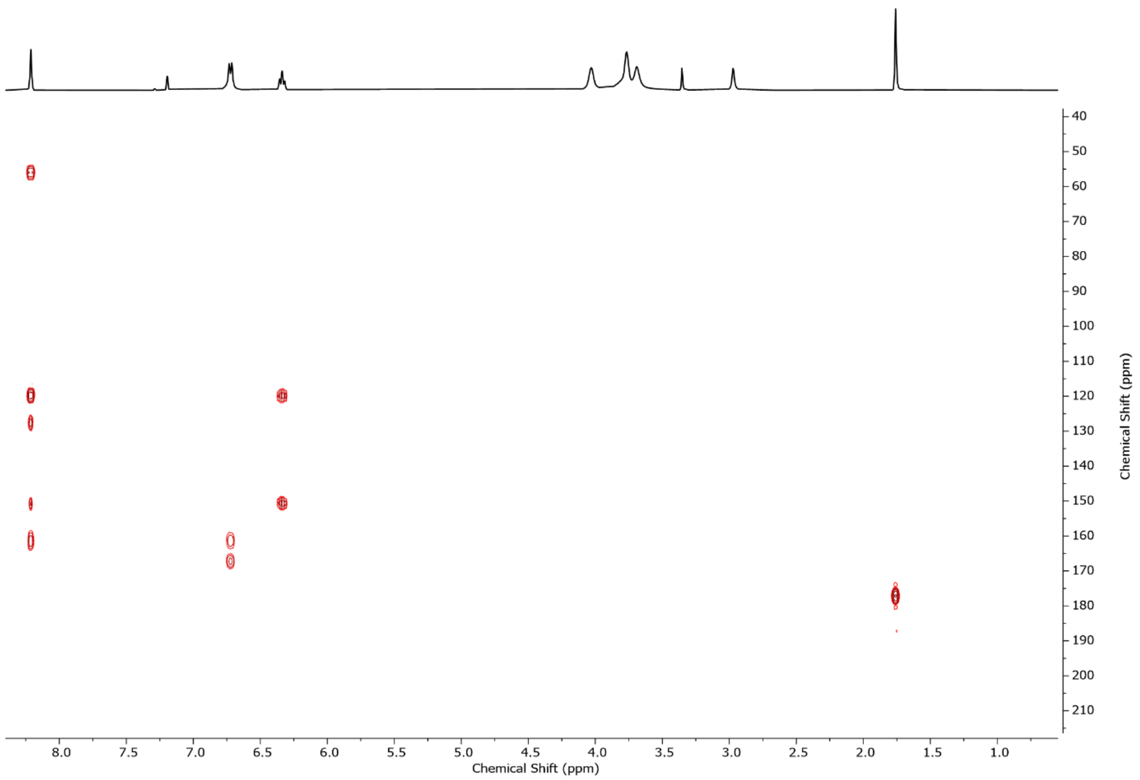

Figure S33. HMBC NMR of **5** ($CDCl_3$, 298K).

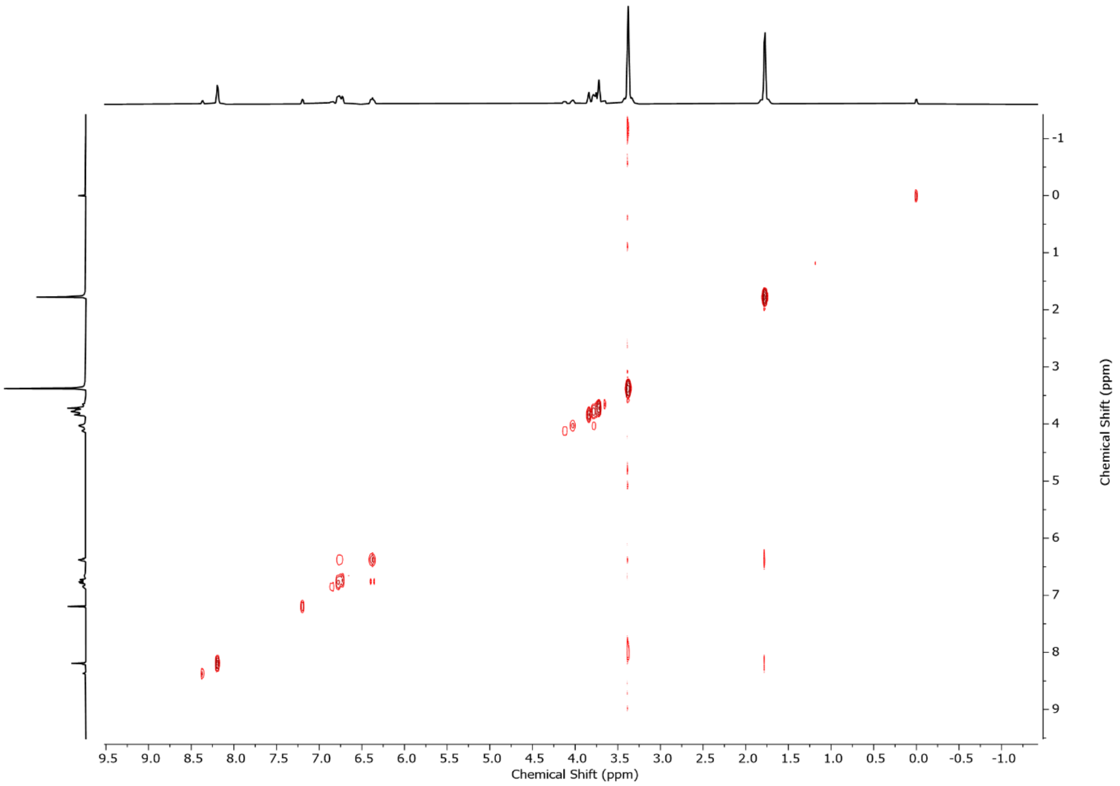

Figure S34. 2D COSY NMR of **6** ($CDCl_3$, 298K).

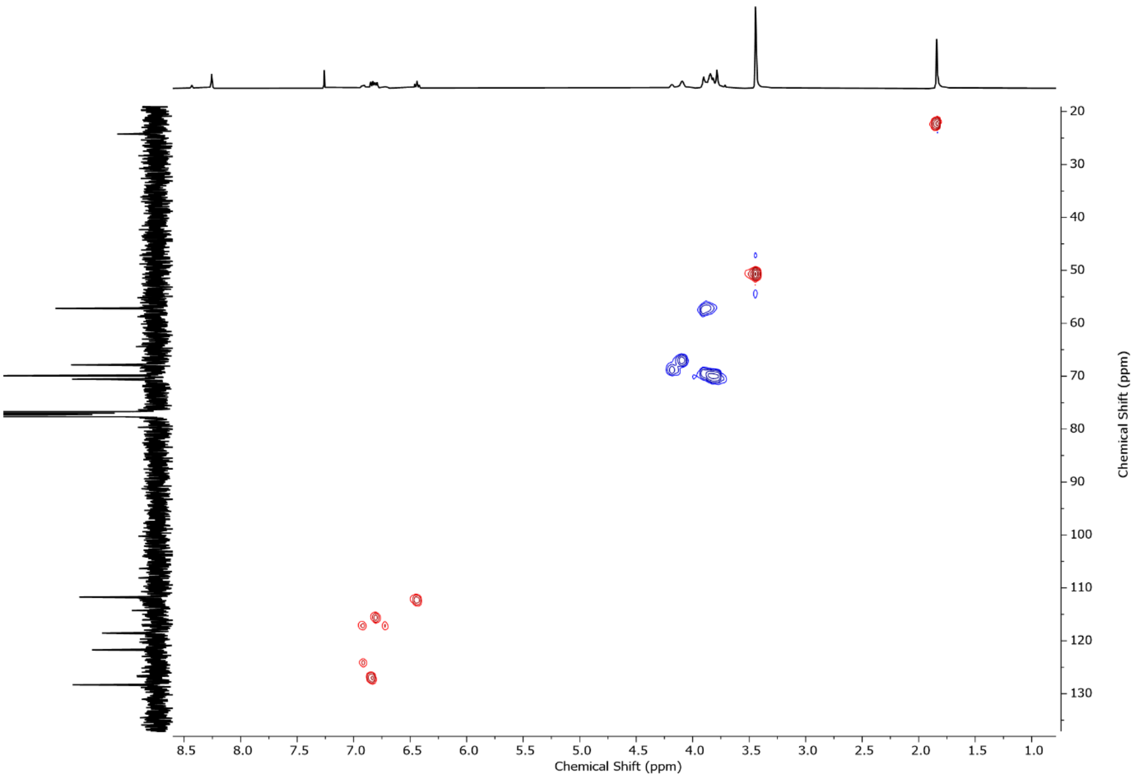

Figure S35. 2D HSQC NMR of **6** ($CDCl_3$, 298K).

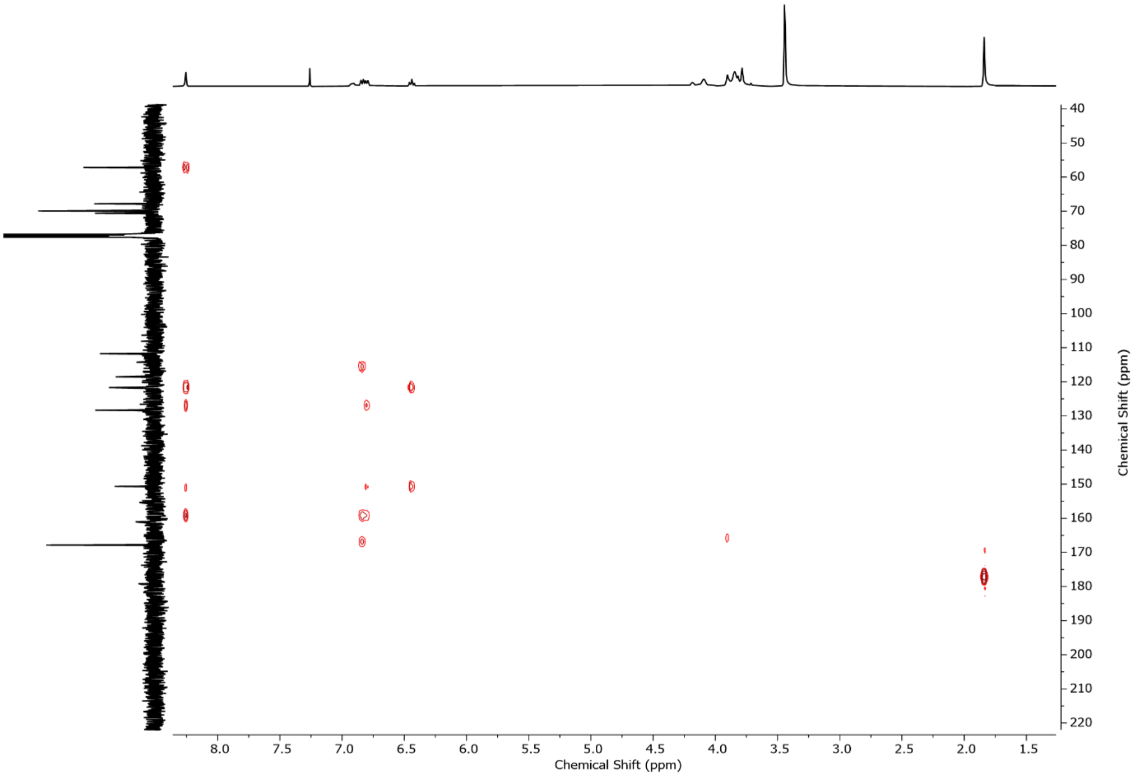

Figure S36. 2D HMBC NMR of **6** ($CDCl_3$, 298K).

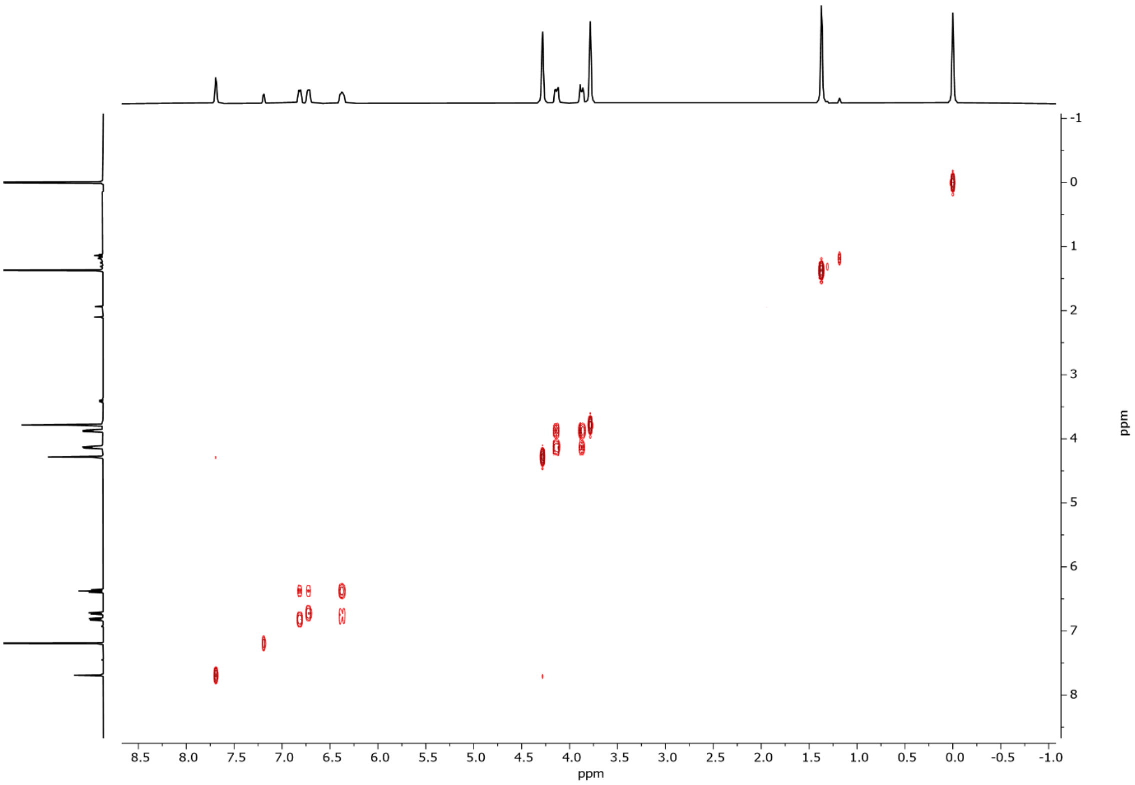

Figure S37. 2D COSY NMR of **7** ($CDCl_3$, 298K).

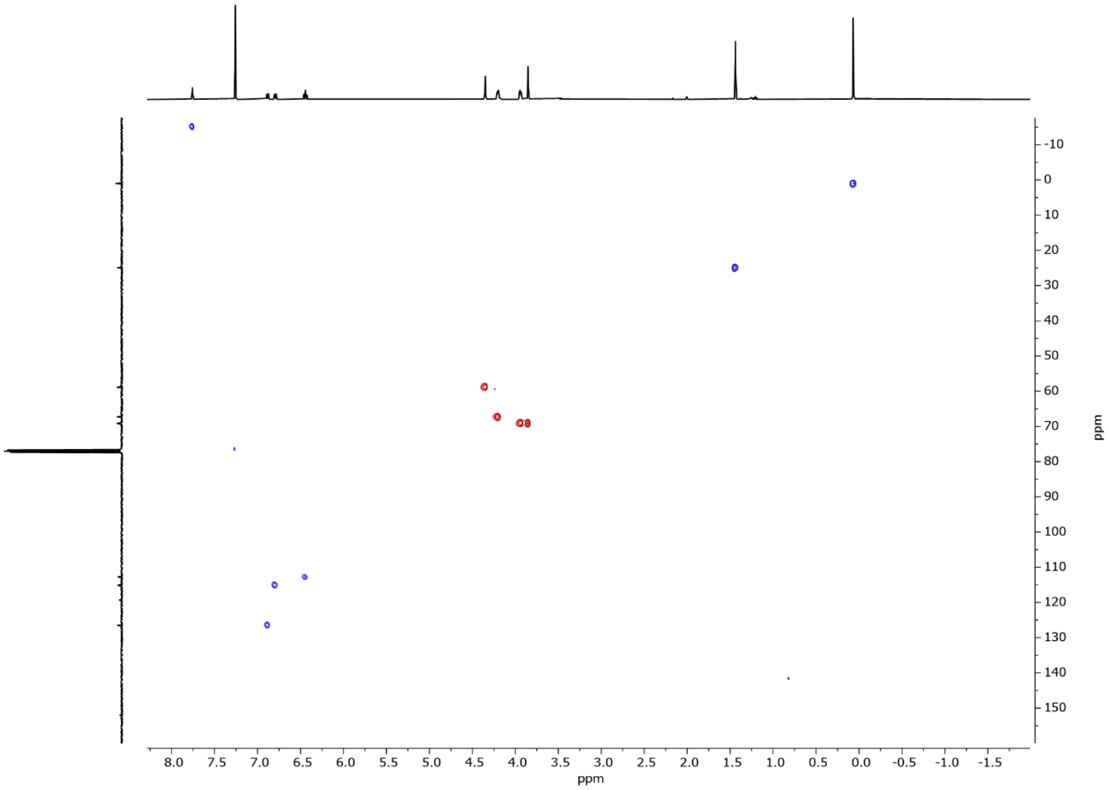

Figure S38. 2D HSQC NMR of **7** ($CDCl_3$, 298K).

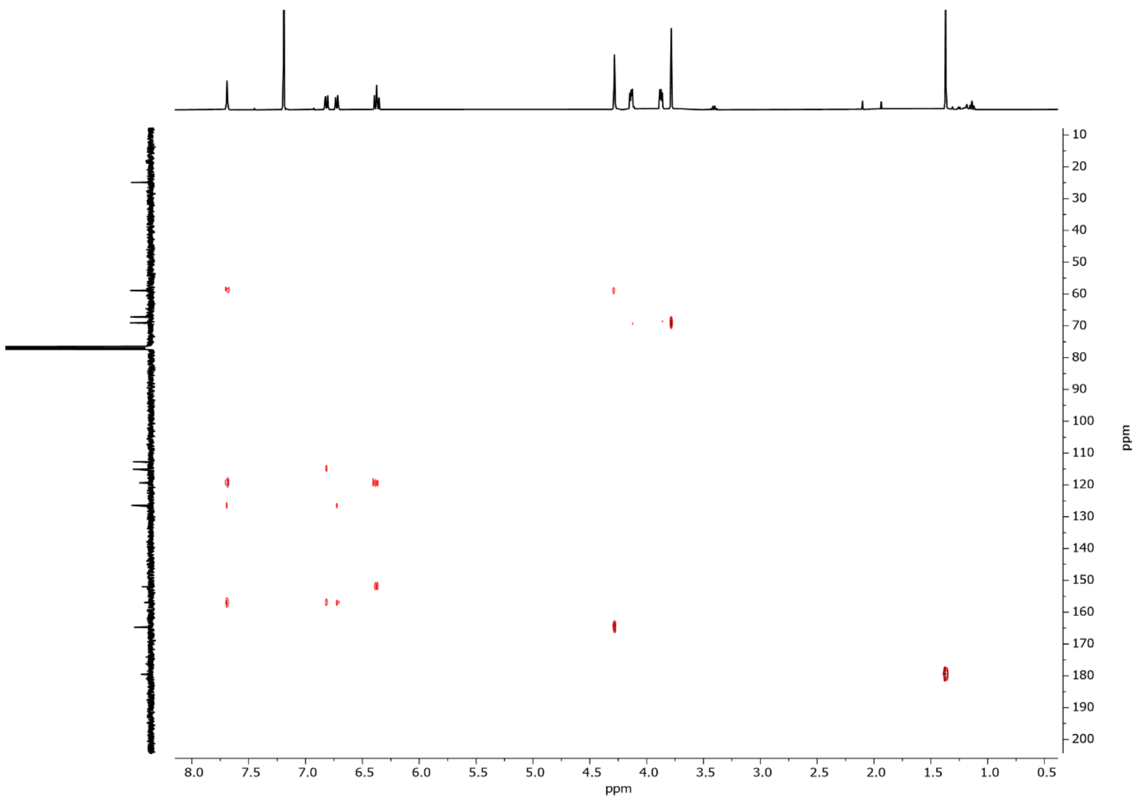

Figure S39. 2D HMBC NMR of **7** ($CDCl_3$, 298K)

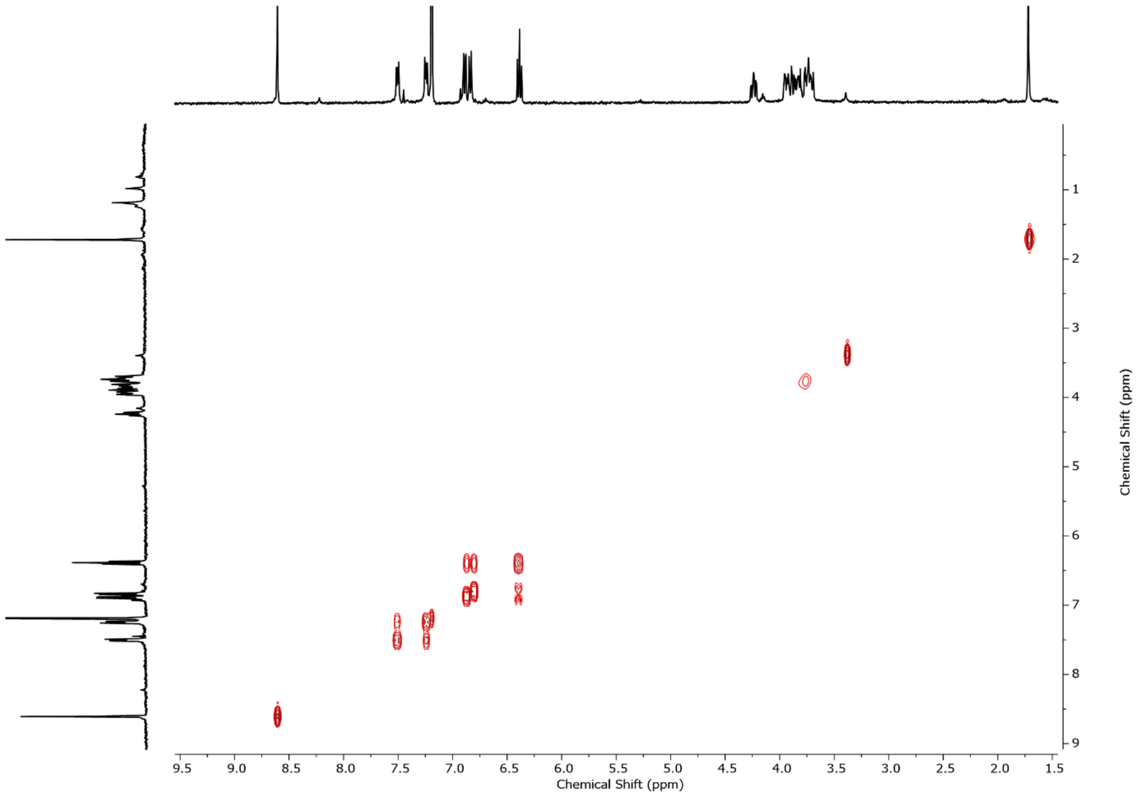

Figure S40. 2D COSY NMR of **8** ($CDCl_3$, 298K).

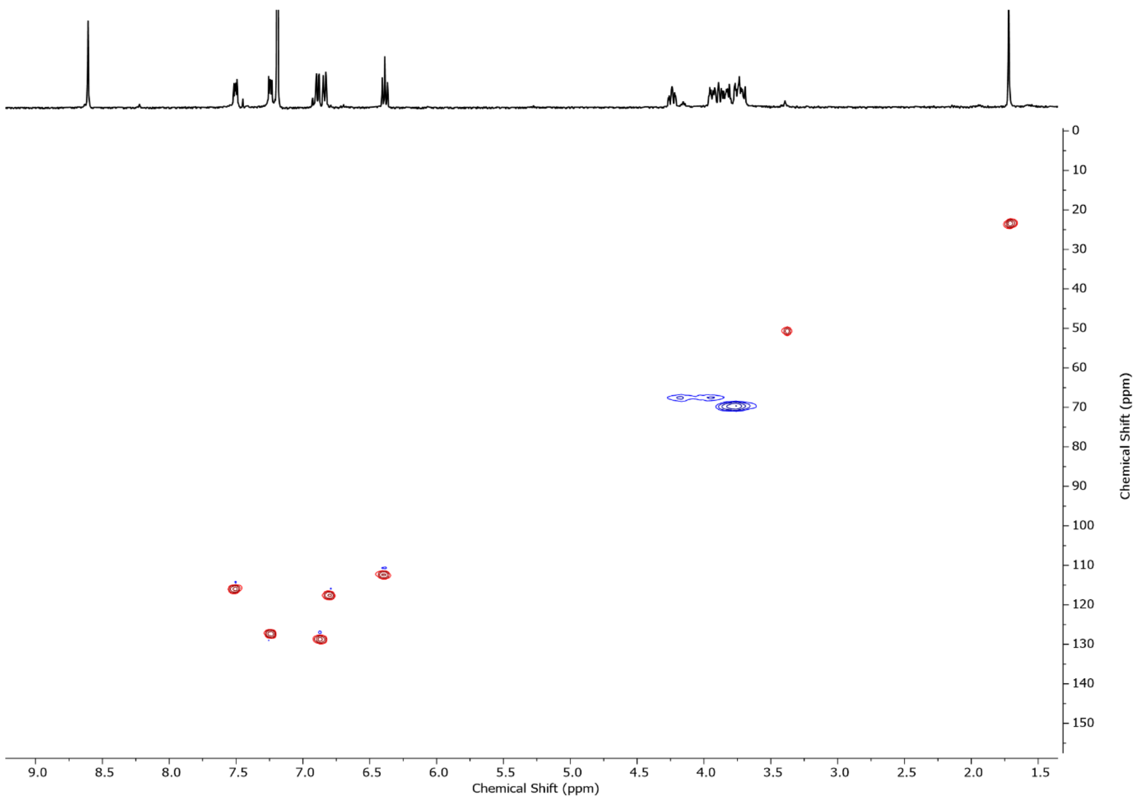

Figure S41. 2D HSQC NMR of **8** ($CDCl_3$, 298K).

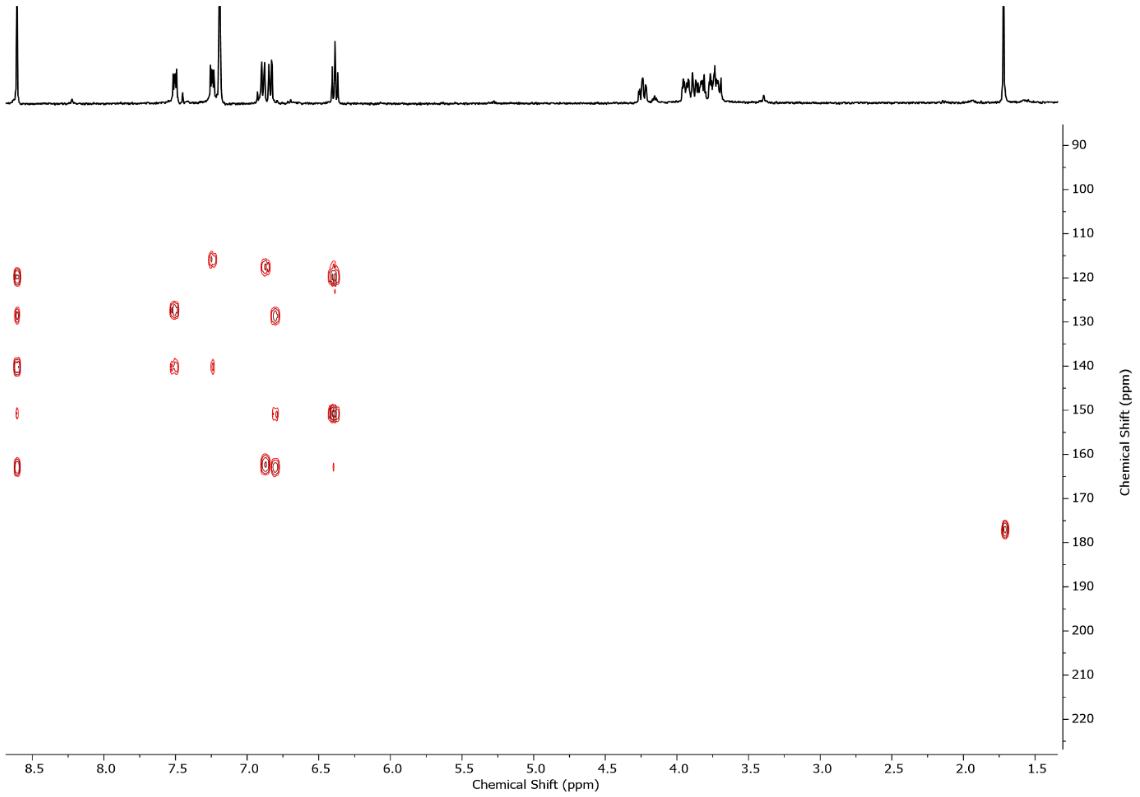

Figure S42. 2D HMBC NMR of **8** ($CDCl_3$, 298K).

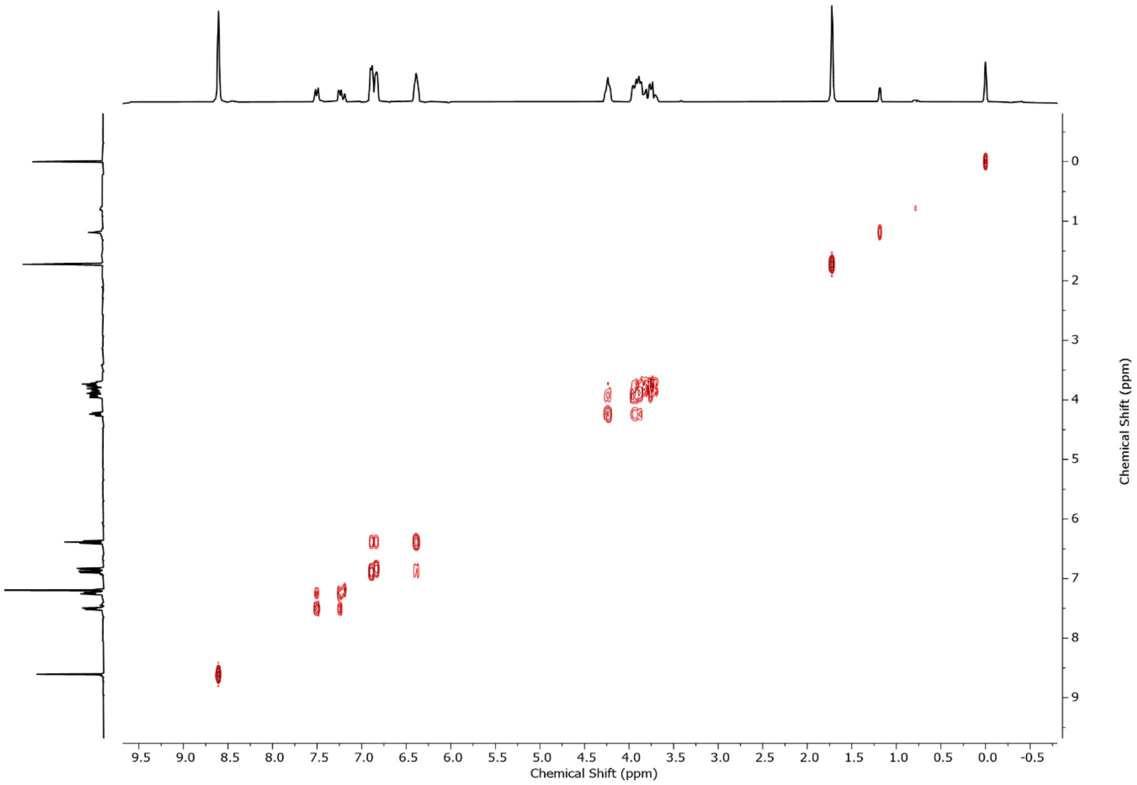

Figure S43. 2D COSY NMR of **9** ($CDCl_3$, 298K).

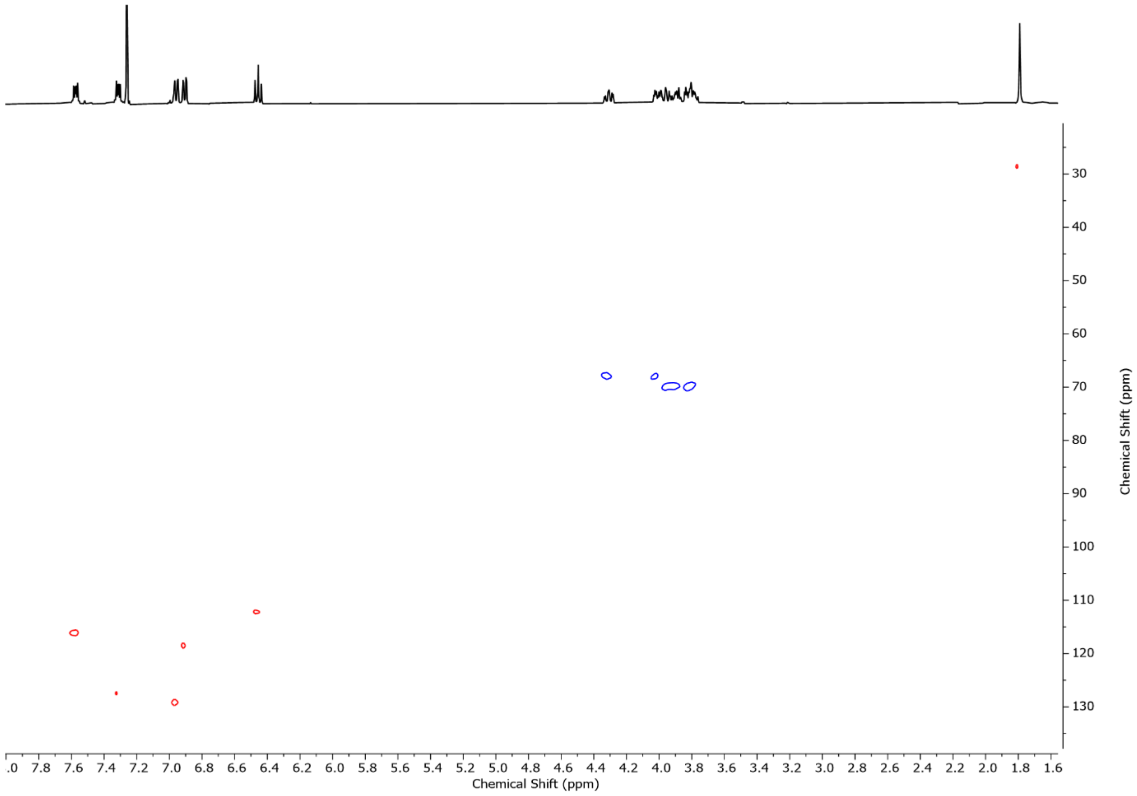

Figure S44. 2D HSQC NMR of **9** ($CDCl_3$, 298K).

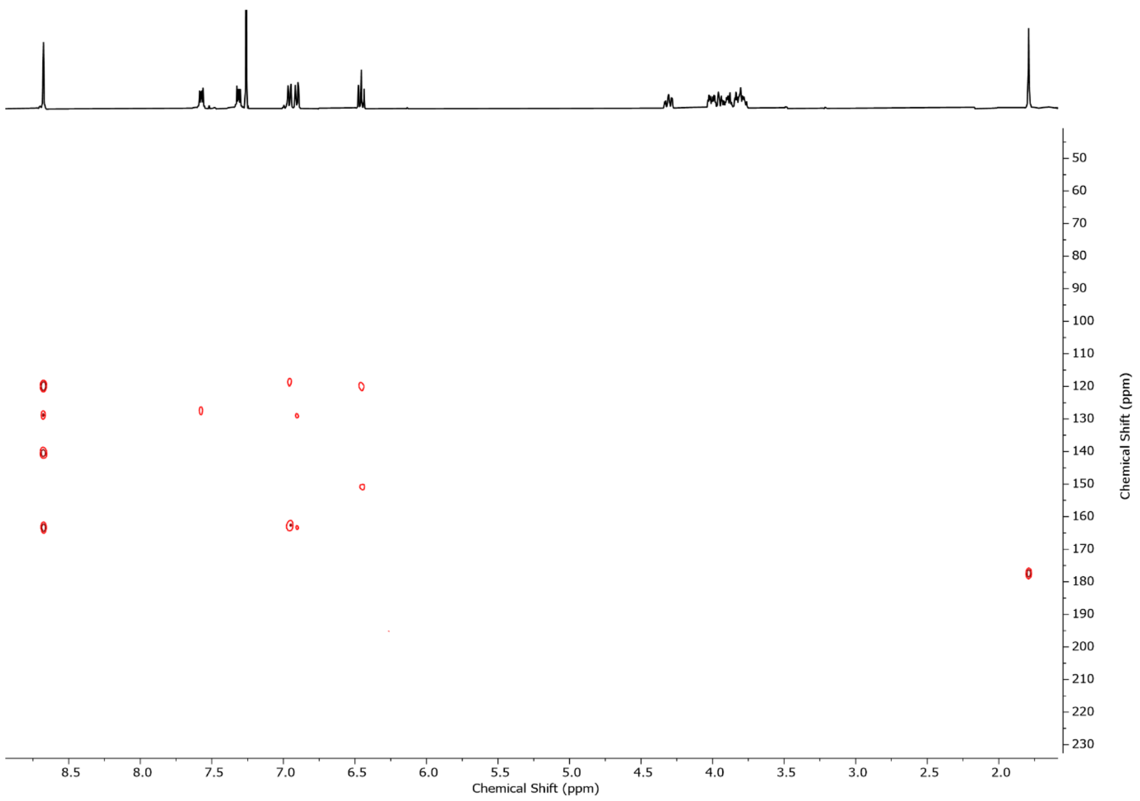

Figure S45. 2D HMBC NMR of **9** ($CDCl_3$, 298K).

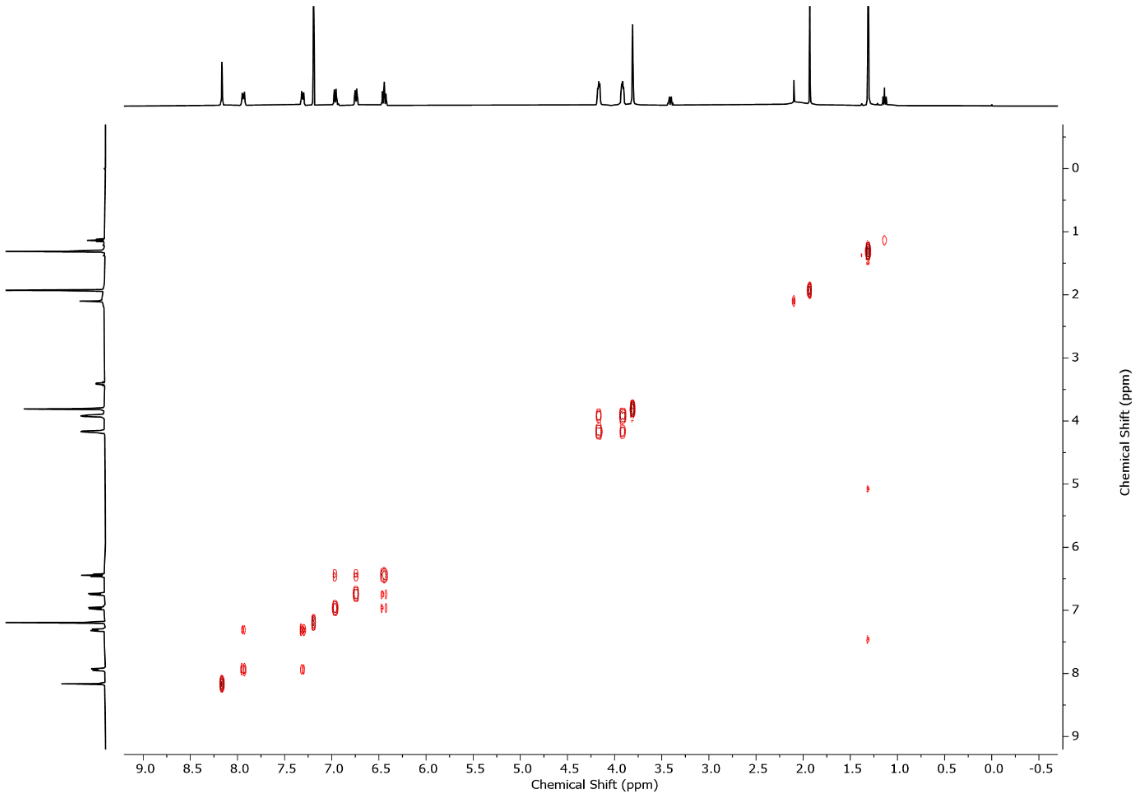

Figure S46. 2D COSY NMR of **10** ($CDCl_3$, 298K).

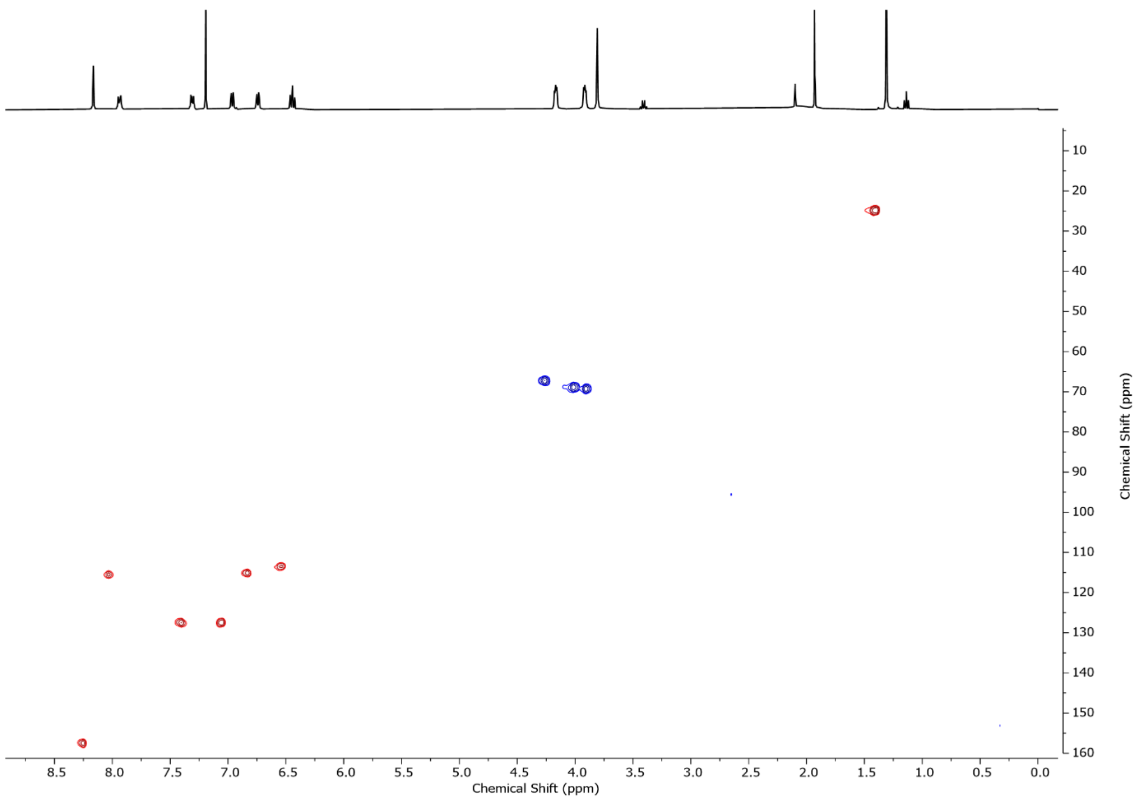

Figure S47. 2D HSQC NMR of **10** ($CDCl_3$, 298K).

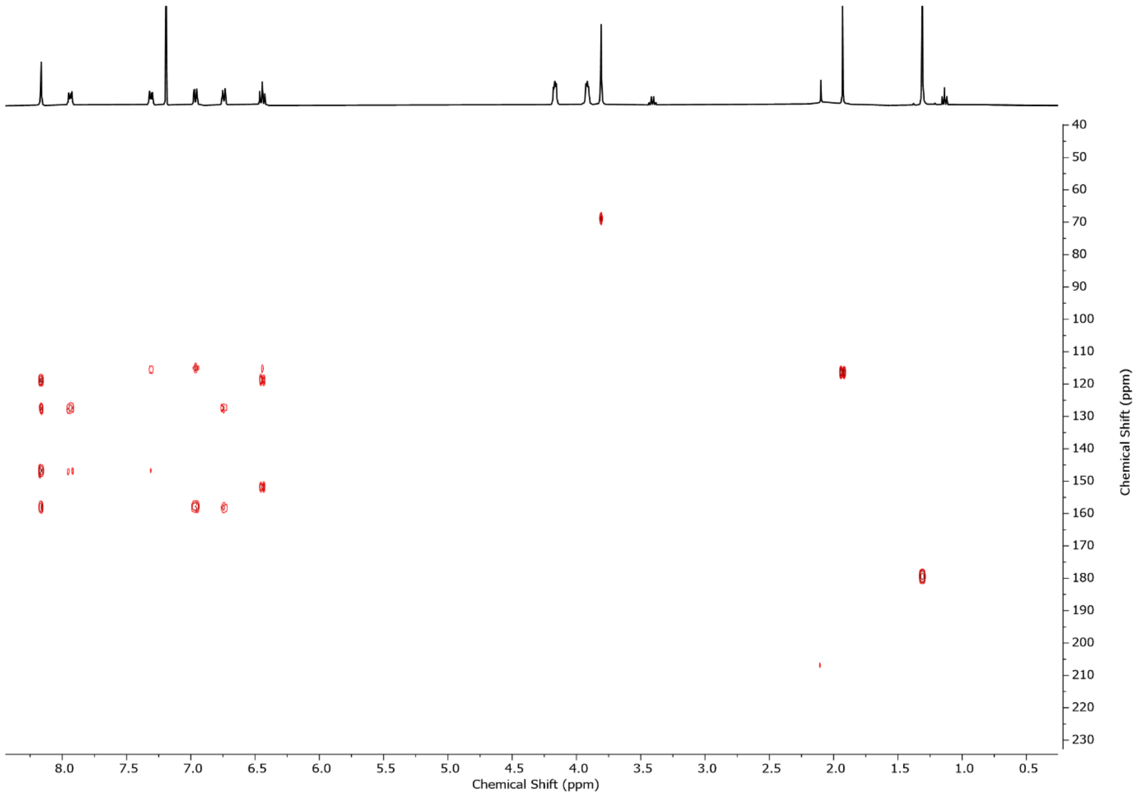

Figure S48. 2D HMBC NMR of **10** ($CDCl_3$, 298K).

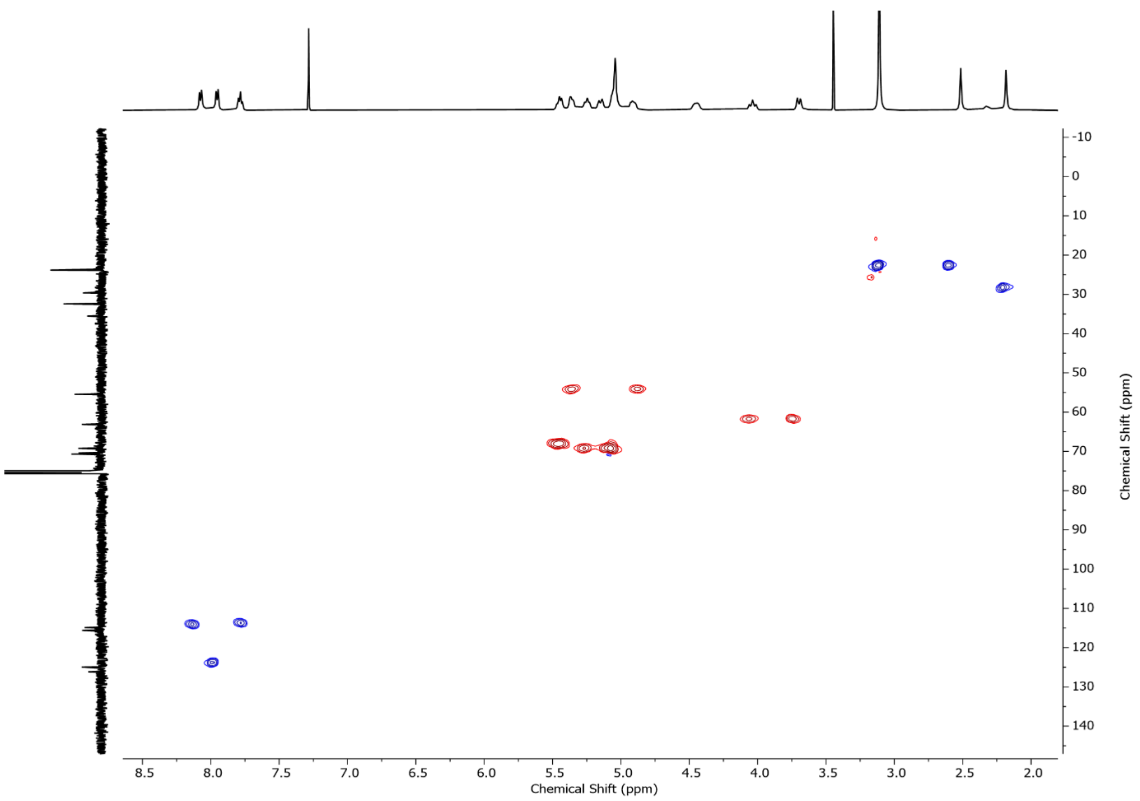

Figure S49. 2D HSQC NMR of **11** (d_2-TCE, 298K).

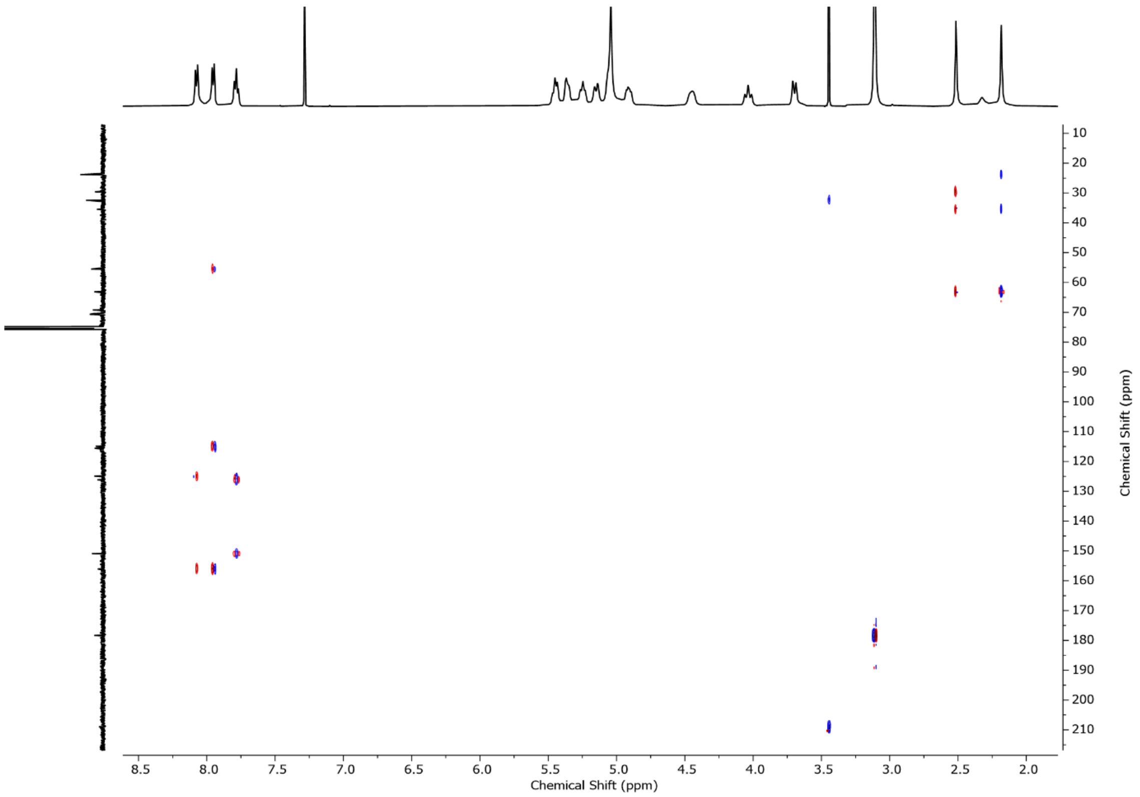

Figure S50. 2D HMBC NMR of **11** (d_2-TCE, 298K).

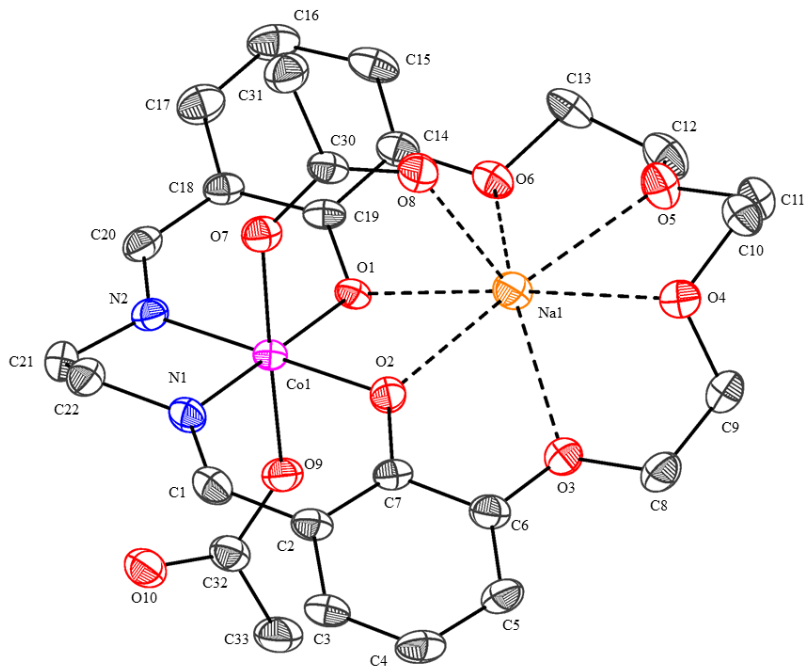

Figure S51. Molecular structures, determined by X-ray diffraction experiments, presented as thermal displacement ellipsoid plots (50% probability) for **7**, fully labeled. H-atoms are removed for clarity and color coding used is M (pink), Na (orange), O (red), N (blue), C (grey).

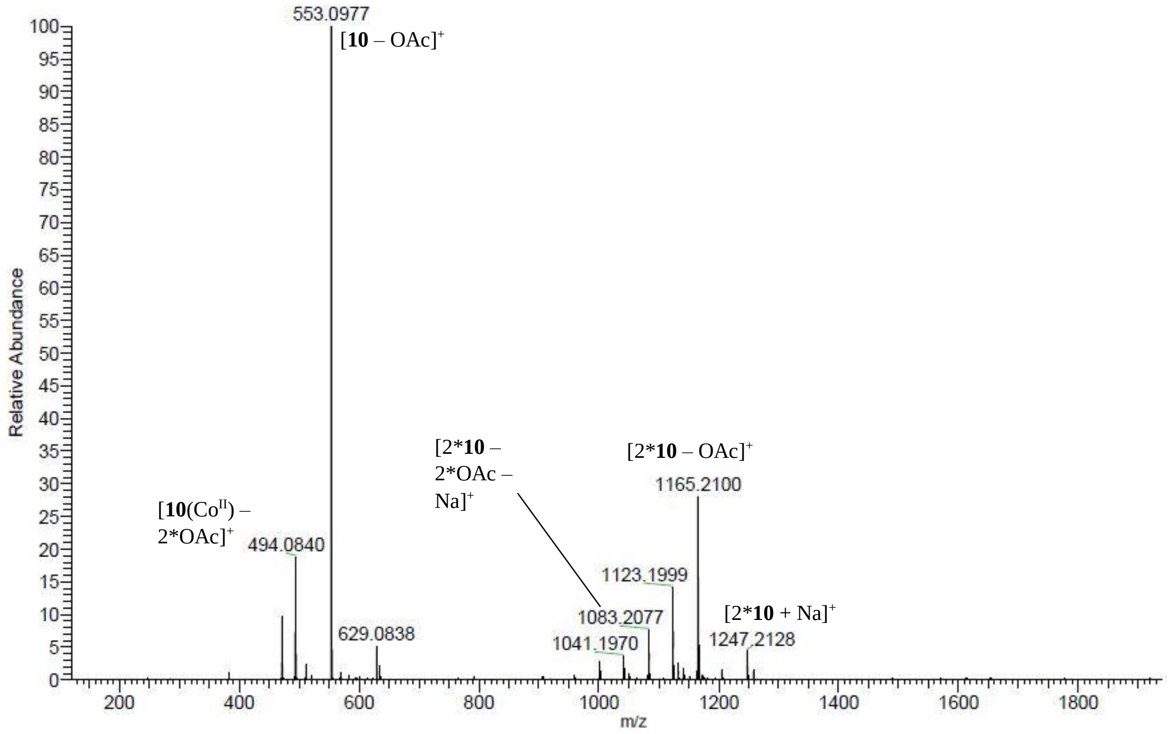

Figure S52. ESI-Q Mass spectrum for **10**, with assignment of principal fragmentation ions.

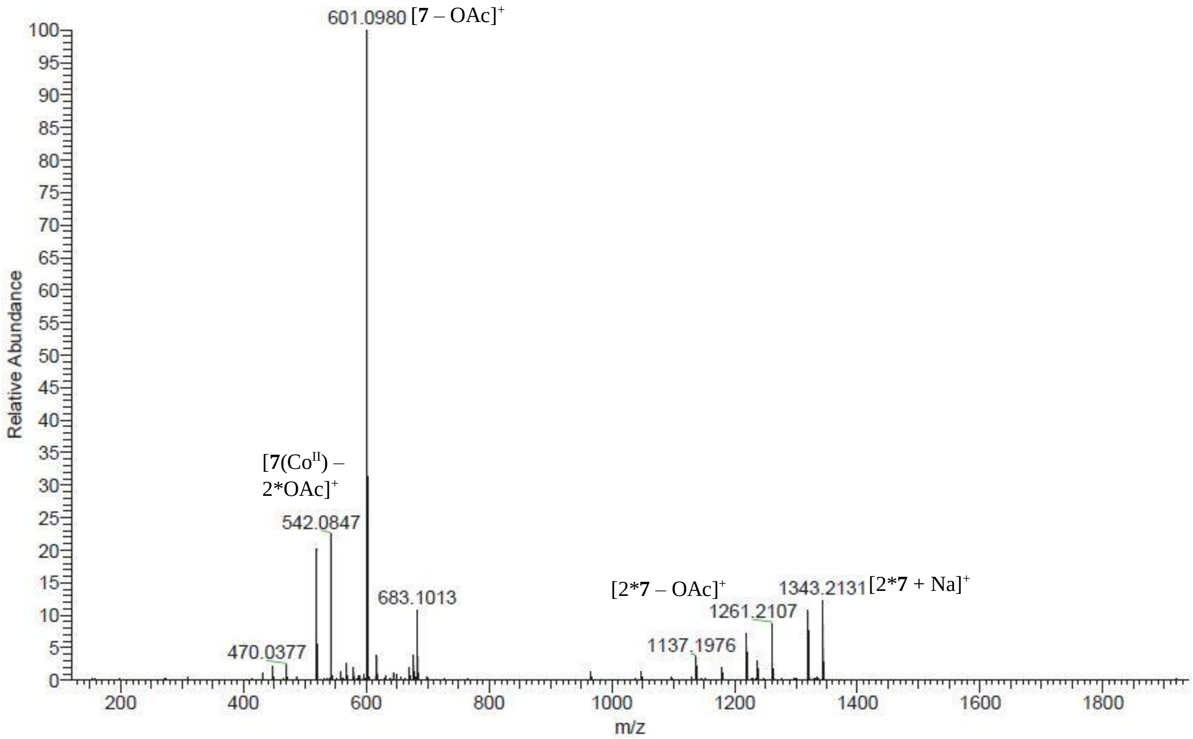

Figure S53. ESI-Q Mass spectrum for **7**, with assignment of principal fragmentation ions.

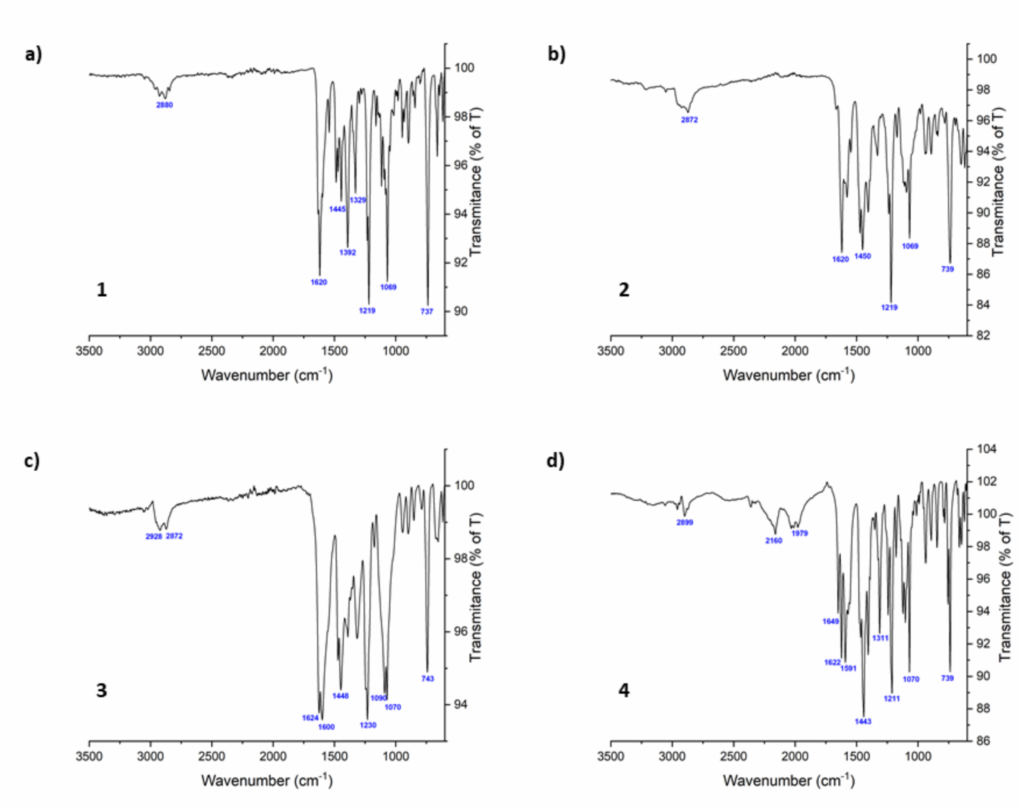

Figure S54. IR spectra of complexes a) **1**, b) **2**, c) **3** and d) **4**.

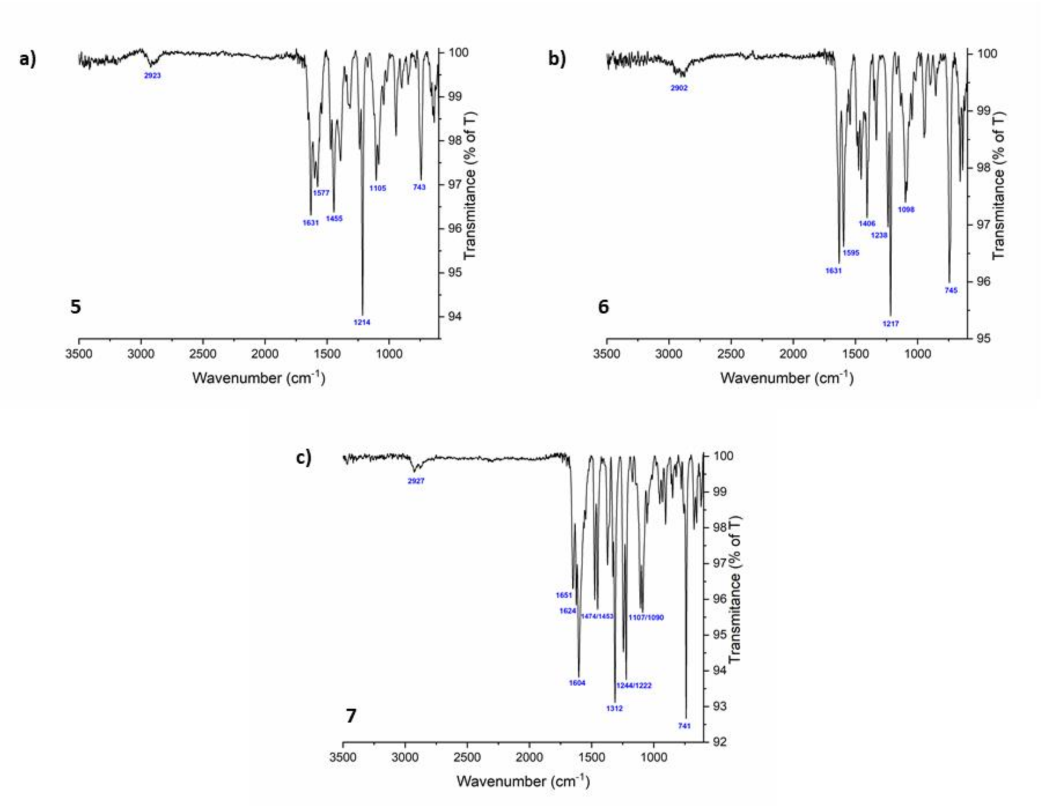

Figure S55. IR spectra of complexes a) **5**, b) **6** and c) **7**.

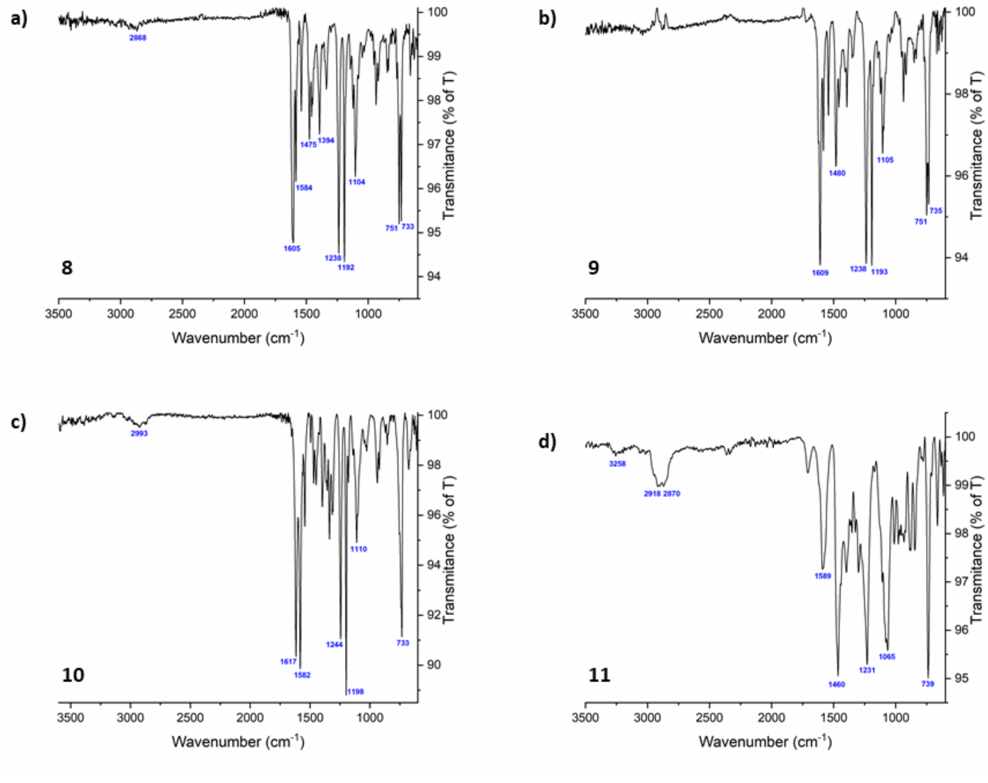

Figure S56. IR spectra of complexes a) **8**, b) **9**, c) **10** and d) **11**.

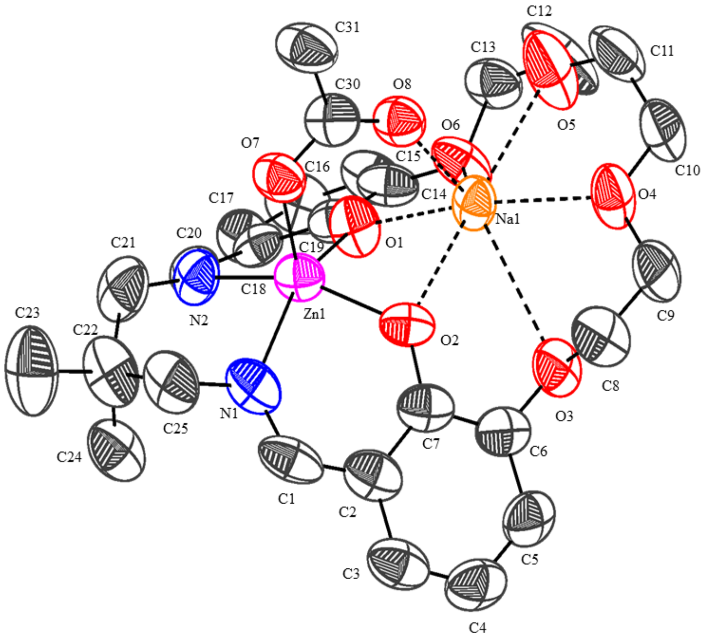

Figure S57. Molecular structures, determined by X-ray diffraction experiments, presented as thermal displacement ellipsoid plots (50% probability) for **1**, fully labeled. H-atoms are removed for clarity and color coding used is M (pink), Na (orange), O (red), N (blue), C (grey).

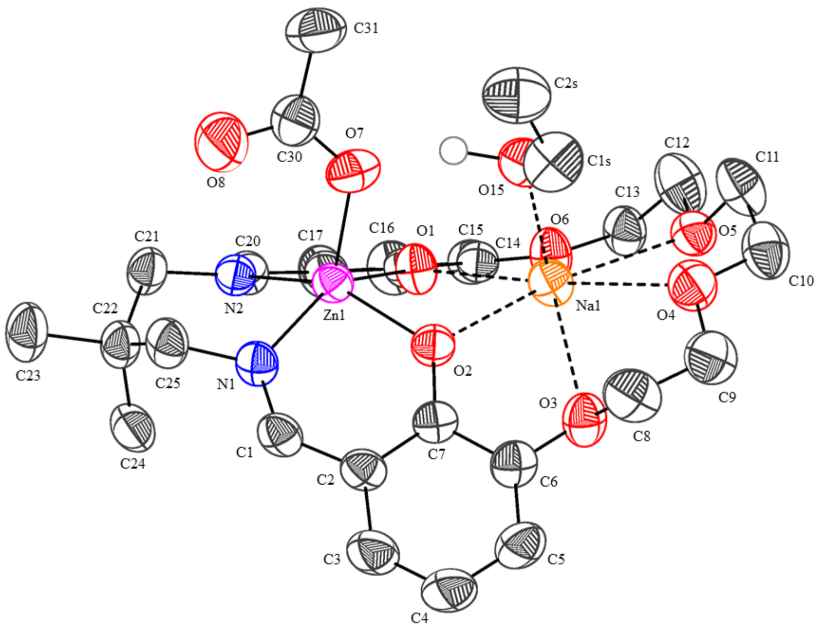

Figure S58. Molecular structures, determined by X-ray diffraction experiments, presented as thermal displacement ellipsoid plots (50% probability) for **1**(EtOH), fully labeled. H-atoms are removed for clarity and color coding used is M (pink), Na (orange), O (red), N (blue), C (grey).

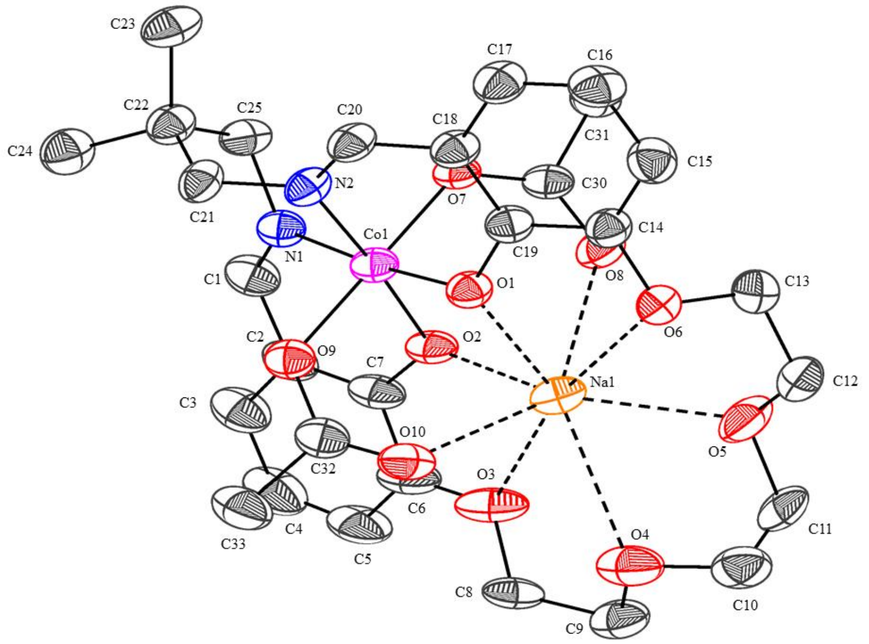

Figure S59. Molecular structures, determined by X-ray diffraction experiments, presented as thermal displacement ellipsoid plots (50% probability) for **3**, fully labeled. H-atoms are removed for clarity and color coding used is M (pink), Na (orange), O (red), N (blue), C (grey).

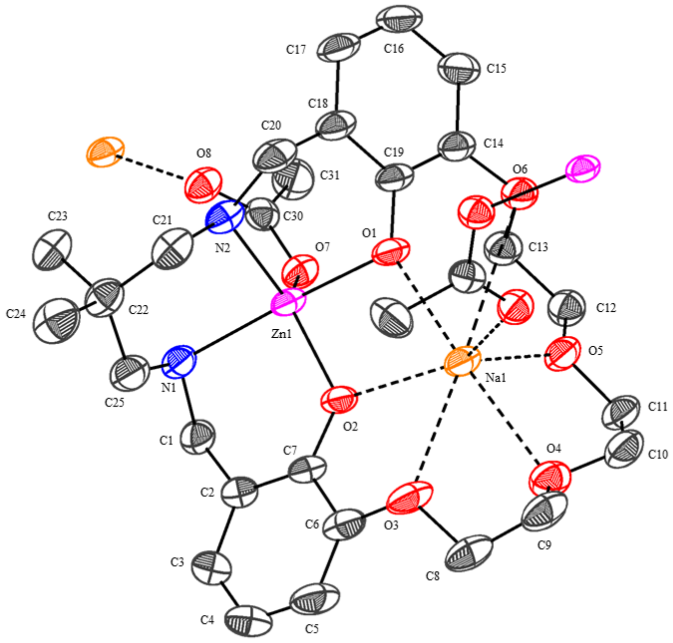

Figure S60. Molecular structures, determined by X-ray diffraction experiments, presented as thermal displacement ellipsoid plots (50% probability) for **11**, fully labeled. H-atoms are removed for clarity and color coding used is M (pink), Na (orange), O (red), N (blue), C (grey).

Table S2. Selected bond lengths from **1**.

Atoms	Distance (Å)	Atoms	Angles (º)
Zn1-O1	2.008(5)	O1-Zn1-O2	84.1(2)
Zn1-O2	2.035(6)	O1-Zn1-O7	106.5(2)
Zn1-O7	1.994(5)	O1-Zn1-N2	87.2(3)
Zn1-N1	2.092(7)	N2-Zn1-N1	87.2(3)
Zn1-N2	2.119(7)	O1-Zn1-N1	149.3(3)
Zn1-Na1	3.249(3)	N1-Zn1-O2	86.9(3)
Na1-O1	2.300(7)	O1-Na1-O8	83.9(2)
Na1-O2	2.431(6)	O1-Na1-O2	69.8(2)
Na1-O3	2.926(7)	Zn1-O1-Na1	92.9(2)
Na1-O4	2.478(6)	Zn1-O2-Na1	97.7(2)
Na1-O5	2.580(9)	O7-C30-O8	126.3(6)
Na1-O6	2.686(7)		
Na1-O8	2.387(6)		

Table S3. Selected bond lengths from **1**(EtOH).

Atoms	Distance (Å)	Atoms	Angles (º)
Zn1-O1	2.017(2)	O1-Zn1-O2	81.01(9)
Zn1-O2	2.062(2)	O1-Zn1-O7	95.91(11)
Zn1-O7	1.997(3)	O1-Zn1-N2	88.41(9)
Zn1-N1	2.093(2)	N2-Zn1-N1	88.59(10)
Zn1-N2	2.118(3)	N1-Zn1-O2	86.11(9)
Zn1-Na1	3.498(2)	O1-Na1-O15	91.35(10)
Na1-O1	2.419(2)	O1-Na1-O2	65.77(8)
Na1-O2	2.461(2)	Zn1-O1-Na1	103.69(9)
Na1-O3	2.556(3)	Zn1-O2-Na1	100.93(9)
Na1-O4	2.642(3)	O7-C30-O8	122.1(4)
Na1-O5	2.676(4)		
Na1-O6	2.663(3)		
Na1-O15	2.366(3)		

Table S4. Selected bond lengths from **3**.

Atoms	Distance (Å)	Atoms	Angles (º)
Co1-O1	1.909(3)	O1-Co1-O2	92.88(13)
Co1-O2	1.914(3)	O1-Co1-O7	92.44(13)
Co1-O7	1.927(3)	O1-Co1-O9	89.71(13)
Co1-O9	1.915(3)	O1-Co1-N2	89.24(14)
Co1-Na1	3.194(2)	N2-Co1-N1	87.38(15)
Na1-O1	2.330(3)	N1-Co1-O2	90.75(15)
Na1-O2	2.334(4)	O1-Na1-O8	70.85(13)
Na1-O3	2.731(4)	O1-Na1-O2	72.88(11)
Na1-O4	2.781(4)	Co1-O1-Na1	97.25(13)
Na1-O5	2.576(5)	Co1-O2-Na1	96.98(13)
Na1-O6	2.725(4)	O7-C30-O8	128.6(5)
Na1-O8	2.450(4)	O9-C32-O10	128.3(4)
Na1-O10	2.527(4)		

Table S5. Selected bond lengths from **7**.

Atoms	Distance (Å)	Atoms	Angles (º)
Co-O1	1.902(3)	O1-Co1-O2	86.55(13)
Co-O2	1.888(3)	O1-Co1-O7	91.44(14)
Co-O9	1.935(3)	O1-Co1-O9	86.43(14)
Co-O7	1.917(3)	O1-Co1-N2	94.74(15)
Co-Na	3.388(2)	N2-Co1-N1	84.86(17)
Na-O1	2.521(3)	N1-Co1-O2	93.85(15)
Na-O2	2.354(4)	O1-Na1-O8	75.99(12)
Na-O8	2.274(4)	O1-Na1-O2	64.29(11)
Na-O3	2.683(4)	Co1-O1-Na1	99.06(13)
Na-O4	2.792(4)	Co1-O2-Na1	105.47(15)
Na-O5	2.543(4)	O7-C30-O8	127.9(5)
Na-O6	2.687(4)	O9-C32-O10	126.9(5)

Table S6. Selected bond lengths from **11**.

Atoms	Distance (Å)	Atoms	Angles (º)
Zn1-O1	2.061(1)	O1-Zn1-O2	84.49(4)
Zn1-O2	1.960(1)	O1-Zn1-O7	95.36(5)
Zn1-O7	1.995(1)	O1-Zn1-N2	90.17(5)
Zn1-Na1	3.327(1)	N2-Zn1-N1	86.87(5)
Na1-O1	2.287(1)	O1-Zn1-N1	90.71(5)
Na1-O2	2.289(1)	N1-Zn1-O2	169.90(5)
Na1-O3	2.741(2)	O1-Na1-O8	91.34(4)
Na1-O4	2.533(1)	O2-Na1-O8	132.35(5)
Na1-O5	2.385(1)	O1-Na1-O2	72.44(4)
Na1-O6	3.527(2)	Zn1-O1-Na1	99.65(4)
Na1-O8	2.314(1)	Zn1-O2-Na1	102.85(5)
		O7-C30-O8	125.17(14)

Table S7. Selected Crystallographic details.

Compound	**1(EtOH)** (*108ckw18*)	**1** (*115ckw18*)	**3** (*122ckw18*)	**11** (*126ckw18*)
Chemical formula	$C_{29}H_{39}N_2NaO_9Zn$	$C_{27}H_{33}N_2NaO_8Zn$	$C_{29}H_{36}CoN_2NaO_{10}$	$C_{57}H_{80}N_4Na_2O_{17}Zn_2$
Formula weight	647.98	601.91	654.52	1269.97
Collection Temperature (K)	150(2)	150(2)	150(2)	150(2)
Space group	Orthorhombic, P*bca*	Hexagonal, P63	Triclinic, P-1	Monoclinic, I 2/*a*
a (Å)	21.0390(5)	22.3411(2)	8.1892(9)	18.6135(5)
b (Å)	11.4575(2)	22.3411(2)	9.7679(12)	13.6907(3)
c (Å)	24.7143(5)	9.9684(1)	19.7756(18)	23.9305(4)
α (º)	90	90	79.710(9)	90
β (º)	90	90	78.414(9)	92.553(2)
γ (º)	90	120	69.342(11)	90
V ($Å^3$)	5957.5(2)	4308.89(9)	1439.9(3)	6092.2(2)
Z	8	6	2	4
D_{calc} (Mg/m^3)	1.445	1.392	1.510	1.385
Crystal size (mm)	0.20 X 0.08 X 0.02	0.30 X 0.05 X 0.05	0.20 X 0.08 X 0.08	0.25 X 0.15 X 0.08
Theta range for data collection (º)	3.577 to 76.135	3.957 to 76.207	2.297 to 76.500	3.698 to 76.191
Source, μ (mm^{-1})	(Cu, Kα) 1.765	(Cu, Kα) 1.762	(Cu, Kα) 5.355	(Cu, Kα) 1.700
Reflections collected	37376	49588	11032	23591
Unique reflections, R_{int}	6202 [R_{int} = 0.0481]	5962 [R_{int} = 0.0365]	5909 [R_{int} = 0.0704]	6322 [R_{int} = 0.0214]
Data Completeness to [θ]	100.0% [67.684]	100.0% [67.684]	99.2% [67.684]	100.0% [67.684]
Data/restraints/parameters	6202 / 9 / 397	5962 / 1 / 356	5909 / 60 / 430	6322 / 0 / 402
R1 (all data)	0.0521 (0.0688)	0.0491 (0.0499)	0.0660 (0.1058)	0.0293 (0.0330)
wR2 (all data)	0.1464 (0.1616)	0.1358 (0.1370)	0.1526 (0.1817)	0.0784 (0.0814)
Goodness-of-fit	1.030	1.035	1.016	1.044
Largest diff. peak and hole (e $Å^{-3}$)	0.830 and -0.462	0.531 and -0.340	0.788 and -0.406	0.969 and -0.284

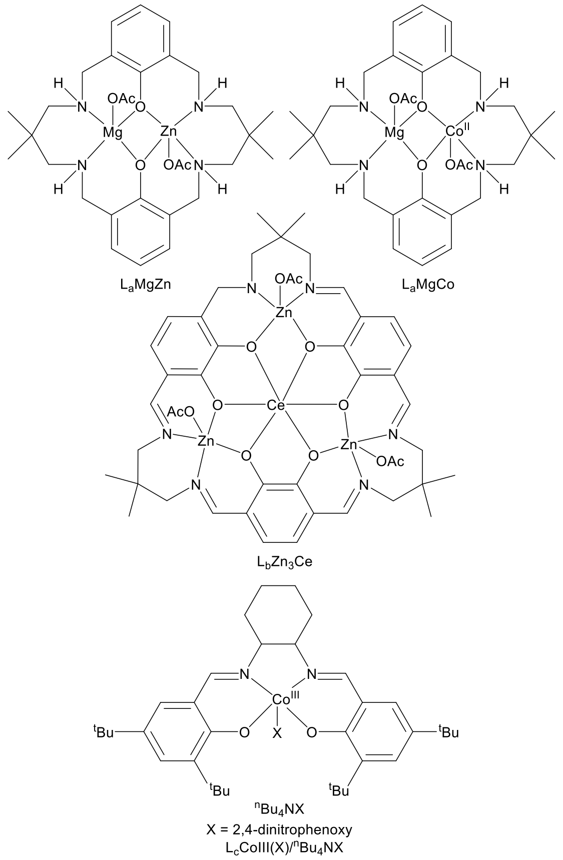

Figure S61. Chemical structure of literature catalysts $L_{\mathbf{a}}MgZn$, $L_{\mathbf{a}}MgCo$, $L_{\mathbf{b}}Zn_3Ce$ and $L_{\mathbf{c}}Co^{III}(X)/^{n}Bu_4NX$.[6]

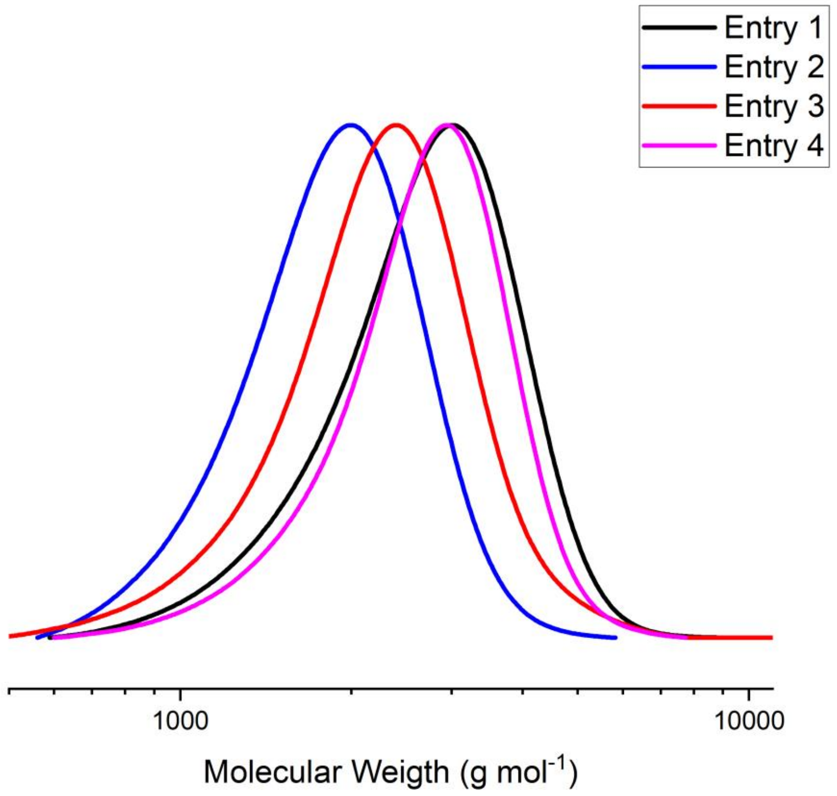

Figure S62. GPC data for entries 1 – 4 of Table 1.

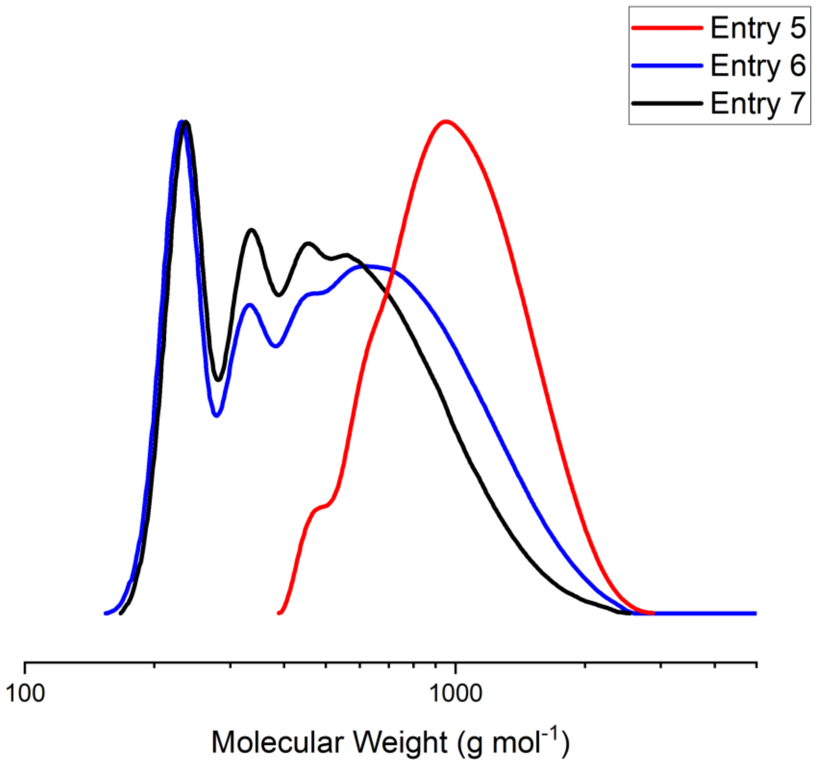

Figure S63. GPC data for entries 6 – 7 of Table 1.

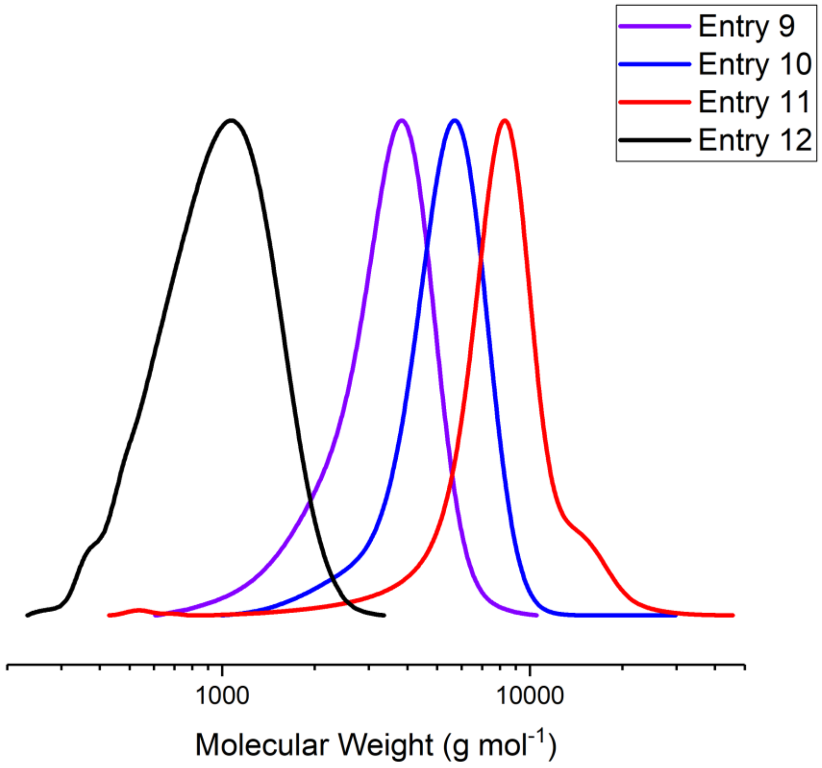

Figure S64. GPC data for entries 9 – 12 of Table 1.

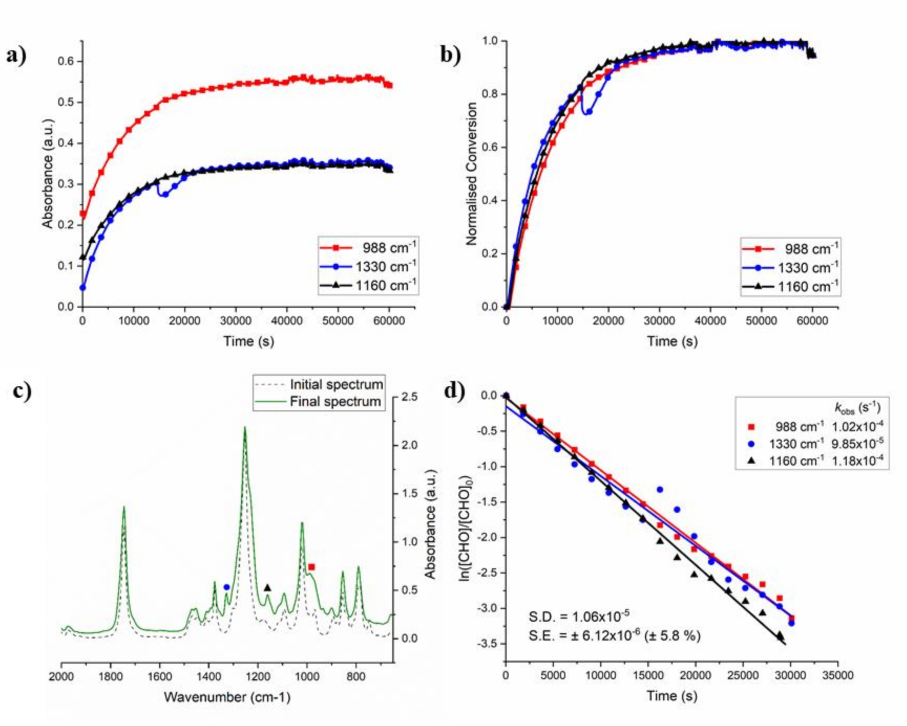

Figure S65. Copolymerization kinetic data (a) Absorbance vs. time plot for the three PCHC wavenumbers (every 30th data point displayed). (b) Normalised conversion vs. time plot for the three PCHC wavenumbers (every 30th data point displayed). (c) ReactIR spectra recorded at the start and finish of the copolymerization. Three peaks associated with PCHC were analysed: 988, 1160 and 1330 cm^{-1}. (d) Logarithm of epoxide concentration vs. time for the three PCHC wavenumbers. k_{obs} (s^{-1}) values are reported and the average value used in subsequent analysis.

Table S8. Variable Temperature Polymerisation Data for Selected Catalysts.

Entry	Catalyst	Cat:CHD:CHO	Temp. (°C)	Time (h)	CO_2 selectivity (%)^a	Polymer Selectivity (%)^b	Conversion (%)^c	TOF (h^{-1})^d	$M_{n, exp.}$ (g mol^{-1}) [$Đ$]^e
1	**8**	1:10:1000	80	4	>99	>99	5	13	600 (1.15)
2	**8**	1:10:1000	100	4	>99	95	14	35	700 (1.22)
3	**8**	1:10:1000	120	4	>99	71	16	40	1000 (1.32)
4	**5**	1:10:1000	80	8	>99	98	22	27	1200 (1.18)
5	**5**	1:10:1000	100	8	>99	94	24	29	1700 (1.13)
6	**5**	1:10:1000	120	8	>99	76	30	37	1800 (1.20)
7	**7**	1:10:5000	80	2	>99	>99	41	1035	14000 (1.05)<br>6000 (1.08)
8	**7**	1:10:5000	100	2	>99	>99	46	1162	16200 (1.04)<br>6900 (1.10)

9	**7**	1:10:5000	120	2	>99	99	41	1036	13900(1.05)<br>6000 (1.08)

a) Expressed as a percentage of CO_2 uptake vs the theoretical maximum (100 %), determined by comparison of the relative integrals of the ^{1}H NMR resonances due to polycarbonate (δ 4.65 ppm), *cis*-cyclic carbonate (4.68 ppm) and *trans*-cyclic carbonate (4.00 ppm) and polyether (δ 3.45 ppm); *b)* Expressed as a percentage of polymer formation vs. the theoretical maximum (100 %), determined by comparison of the relative integrals of the ^{1}H NMR proton resonances due to polymer (4.65 ppm), *cis*-cyclic carbonate (4.68 ppm), *trans*-cyclic carbonate (4.00 ppm) and polyether (δ 3.45 ppm); *c)* Cyclohexene oxide consumed as percentage of total starting amount, determined by ^{1}H NMR spectroscopy; *d)* Turnover frequency (TOF) = moles of CHO consumed/(time x moles of catalyst), error of 5% assumed; *e)* Determined by SEC in THF eluent (RI detector), calibrated against narrow M_n polystyrene standards; polydispersity given in square brackets. GPC traces Figure S66-S68.

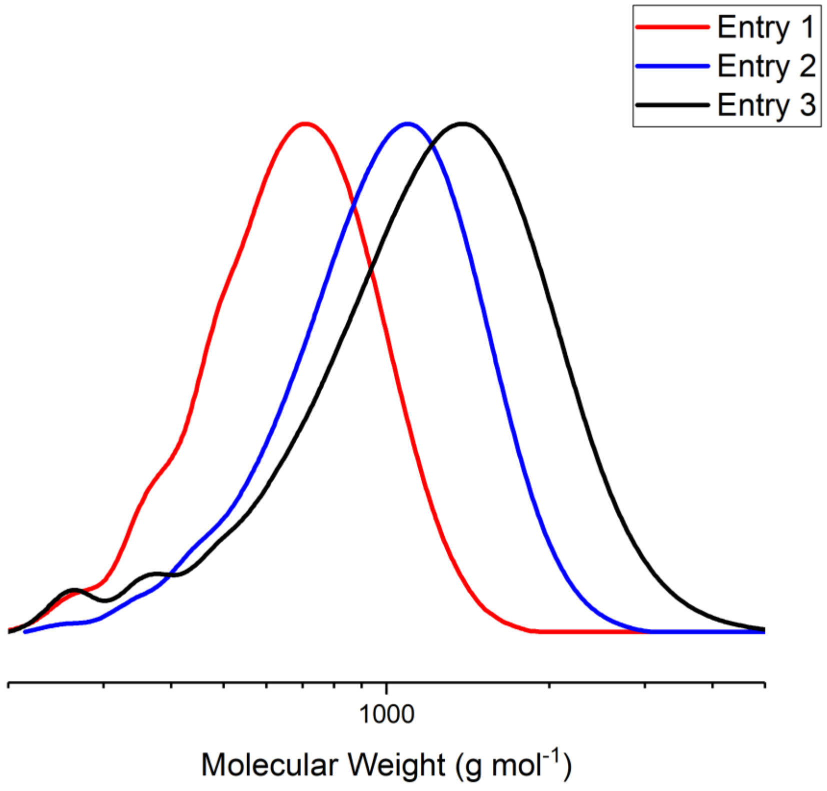

Figure S66. GPC data for entries 1 – 3 of Table S8.

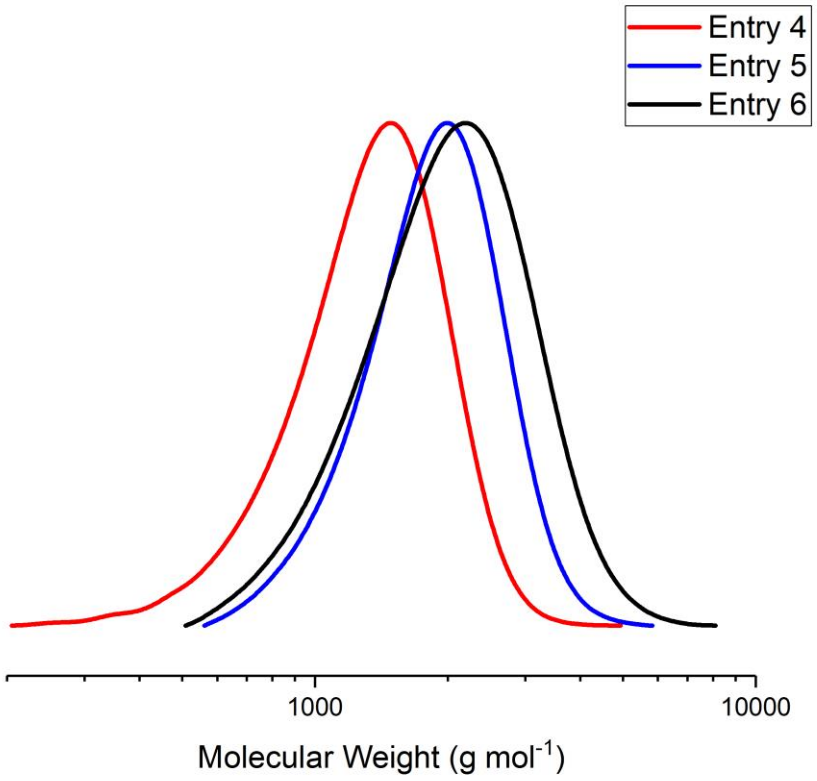

Figure S67. GPC data for entries 4 – 6 of Table S8.

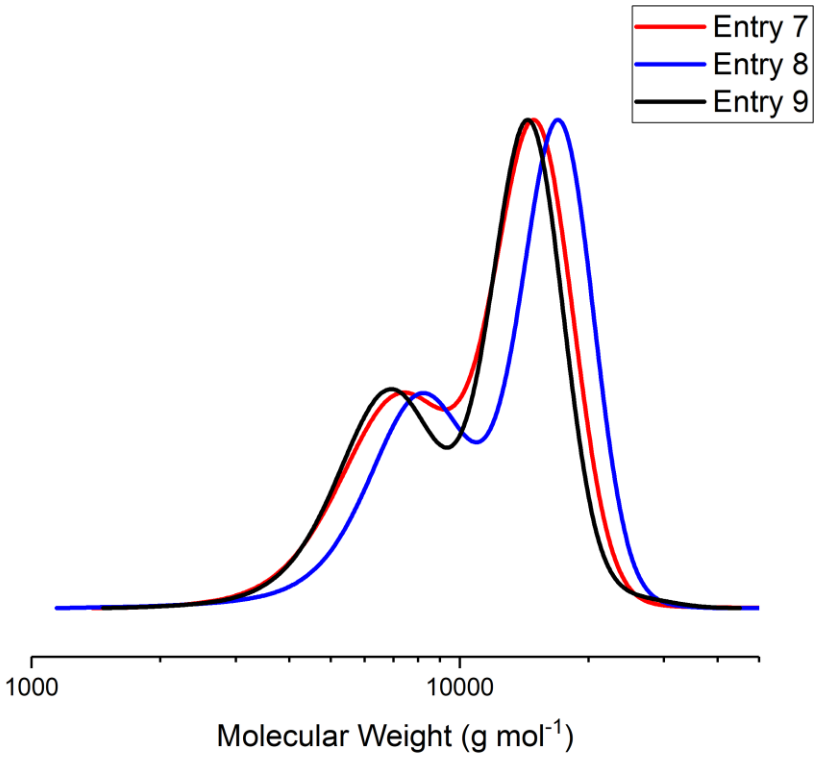

Figure S68. GPC data for entries 7– 9 of Table S.

References

[1] N. Yuntawattana, T. M. McGuire, C. B. Durr, A. Buchard, C. K. Williams, *Catalysis Science & Technology* **2020**, *10*, 7226-7239.

[2] A. T. U. Ltd.

[3] G. M. Sheldrick, *Acta Crystallogr. A* **2008**, *64*, 112-122.

[4] L. J. Farrugia, *J. Appl. Crystallogr.* **2012**, *45*, 849-854.

[5] a) A. L. Spek, *Acta Crystallogr. C* **2015**, *71*, 9-18; b) A. L. Spek, *J. Appl. Crystallogr.* **2003**, 7–13.

[6] a) W.-M. M. Ren, X. Zhang, Y. Liu, J.-F. F. Li, H. Wang, X.-B. B. Lu, *Macromolecules* **2010**, *43*, 1396-1402; b) G. Trott, J. A. Garden, C. K. Williams, *Chem. Sci.* **2019**; c) A. C. Deacy, A. F. R. Kilpatrick, A. Regoutz, C. K. Williams, *Nat. Chem.* **2020**, *12*; d) H. Nagae, R. Aoki, S. N. Akutagawa, J. Kleemann, R. Tagawa, T. Schindler, G. Choi, T. P. Spaniol, H. Tsurugi, J. Okuda, K. Mashima, *Angew. Chem. Int. Ed.* **2018**, *57*, 2492-2496.
